# Supplementary material for: Interventions to minimize blood loss in very preterm infants—A systematic review and meta-analysis
Source: PLoS One. 2021 Feb 8;16(2):e0246353. doi: 10.1371/journal.pone.0246353 (PMC7870155; doi:10.1371/journal.pone.0246353)
Supplement: S1 File — (DOCX) [file pone.0246353.s002.docx]

# **S1 File– Search Strategy**

# Search strategy

# Searches were performed in PubMed, Embase (Elsevier), Cinahl Complete (Ebsco), Cochrane CENTRAL with database-specific subject terms, LILAC (Spanish/Portuguese studies), and in addition clinicaltrials.gov, ITCRP (ongoing or prospective registered trials)

- No language or publication date restriction were used.
- Title/Abstract or Title/Abstract/Keyword restriction for search terms that are not subject terms
- Some of the search strategy components are reused and modified search strategies from previous Cochrane reviews.
- After initial combination search, the search strategy was divided into a multi-stranded search strategy, where the intervention search blocks were searched separately in combination with terms for premature infants and RCT studies. The change of the strategy was undertaken to minimize the risk of unintended exclusion of relevant studies.
- Date of search for all databases: 11 February 2020

## **PubMed**

*Micromethods and Reintroducing blood*

(((((((Infant, very low birth weight[Mesh] OR Infant, premature[Mesh] OR premature[Title/Abstract] OR prematurity[Title/Abstract] OR preterm[Title/Abstract] OR "pre term”[Title/Abstract] OR "low birth weight” [Title/Abstract] OR "low birthweight” [Title/Abstract] OR “very low birth weight” OR VLBW[Title/Abstract] OR LBW[Title/Abstract] OR “extremely low birth weight” OR neonat*[Title/Abstract])) AND ((randomized controlled trial [pt] OR controlled clinical trial [pt] OR randomized [tiab] OR randomised [tiab] OR randomly [tiab] OR trial [tiab] OR groups [tiab]) NOT (animals [mh] NOT humans [mh])))) AND ((Blood specimen collection[MeSH Terms]) OR Monitoring, physiologic[Mesh] OR Point-of-care systems[Mesh] OR Phlebotomy[Mesh]) OR ("blood specimen collection"[Title/Abstract] OR "blood sampling"[Title/Abstract] OR "blood conservation"[Title/Abstract] OR “blood testing” OR "re-introducing blood"[Title/Abstract] OR “reintroducing blood”[Title/Abstract] OR "dried blood spot"[Title/Abstract]) OR phlebotomy[Title/Abstract] OR (“point-of-care”[Title/Abstract] OR “point of care”[Title/Abstract] OR POC[Title/Abstract]) AND (technology[Title/Abstract] OR device[Title/Abstract] OR monitor*[Title/Abstract] OR measur*[Title/Abstract]))))))))) AND ((((((reduc*[Title/Abstract] OR decreas*[Title/Abstract] OR minimiz*[Title/Abstract])) AND (blood loss[Title/Abstract] OR transfusion[Title/Abstract] OR anemia[Title/Abstract])))

*Devices to monitor CO2 levels transcutaneous vs blood sampling*

((((((((((((((((Infant, very low birth weight[Mesh] OR Infant, premature[Mesh] OR premature[Title/Abstract] OR prematurity[Title/Abstract] OR preterm[Title/Abstract] OR "pre term”[Title/Abstract] OR "low birth weight” [Title/Abstract] OR "low birthweight” [Title/Abstract] OR “very low birth weight” OR VLBW[Title/Abstract] OR LBW[Title/Abstract] OR “extremely low birth weight” OR neonat*[Title/Abstract])) AND ((randomized controlled trial [pt] OR controlled clinical trial [pt] OR randomized [tiab] OR randomised [tiab] OR randomly [tiab] OR trial [tiab] OR groups [tiab]) NOT (animals [mh] NOT humans [mh])))))))) AND (Blood specimen collection[MeSH Terms] OR blood gas analysis[MeSH Terms] OR Blood Gas Monitoring, Transcutaneous[MeSH Terms] OR oximetry[MeSH Terms] OR Blood chemical analysis[Mesh] OR "blood specimen collection"[Title/Abstract] OR "blood sampling"[Title/Abstract] OR monitoring, physiologic[MeSH Terms] OR transcutaneous[Title/Abstract] OR monitor*[Title/Abstract] OR ((“point-of-care”[Title/Abstract] OR “point of care”[Title/Abstract] OR POC[Title/Abstract]) AND (technology[Title/Abstract] OR device[Title/Abstract] OR monitor*[Title/Abstract] OR measur*[Title/Abstract]))) AND (Carbon dixoxide/blood[Mesh] OR Carbon dioxide[Mesh] OR "carbon dioxide"[Title/Abstract]))))))

*Devices to monitor O2 levels transcutaneous or intra-arterial vs blood sampling*

(((((((((((((((((((Infant, very low birth weight[Mesh] OR Infant, premature[Mesh] OR premature[Title/Abstract] OR prematurity[Title/Abstract] OR preterm[Title/Abstract] OR "pre term”[Title/Abstract] OR "low birth weight” [Title/Abstract] OR "low birthweight” [Title/Abstract] OR “very low birth weight” OR VLBW[Title/Abstract] OR LBW[Title/Abstract] OR “extremely low birth weight” OR neonat*[Title/Abstract])) AND ((randomized controlled trial [pt] OR controlled clinical trial [pt] OR randomized [tiab] OR randomised [tiab] OR randomly [tiab] OR trial [tiab] OR groups [tiab]) NOT (animals [mh] NOT humans [mh])))))))) AND (Blood specimen collection[MeSH Terms] OR blood gas analysis[MeSH Terms] OR Blood Gas Monitoring, Transcutaneous[MeSH Terms] OR oximetry[MeSH Terms] OR Blood chemical analysis[Mesh] OR "blood specimen collection"[Title/Abstract] OR "blood sampling"[Title/Abstract] OR monitoring, physiologic[MeSH Terms] OR transcutaneous[Title/Abstract] OR monitor*[Title/Abstract] OR Intra-arterial[Title/Abstract] OR intraarterial OR” intra arterial”[Title/Abstract] OR “arterial line”[Title/Abstract] OR “in-line”[Title/Abstract] OR “end-tidal” OR “end tidal” OR ((“point-of-care”[Title/Abstract] OR “point of care”[Title/Abstract] OR POC[Title/Abstract]) AND (technology[Title/Abstract] OR device[Title/Abstract] OR monitor*[Title/Abstract] OR measur*[Title/Abstract])) AND (Oxygen/blood[Mesh] OR oxygen[Title/Abstract])))))))))

*Devices to monitor glucose levels subcutaneous vs blood sampling*

(((((((((((Infant, very low birth weight[Mesh] OR Infant, premature[Mesh] OR premature[Title/Abstract] OR prematurity[Title/Abstract] OR preterm[Title/Abstract] OR "pre term”[Title/Abstract] OR "low birth weight” [Title/Abstract] OR "low birthweight” [Title/Abstract] OR “very low birth weight” OR VLBW[Title/Abstract] OR LBW[Title/Abstract] OR “extremely low birth weight” OR neonat*[Title/Abstract])) AND ((randomized controlled trial [pt] OR controlled clinical trial [pt] OR randomized [tiab] OR randomised [tiab] OR randomly [tiab] OR trial [tiab] OR groups [tiab]) NOT (animals [mh] NOT humans [mh])))) AND ((blood glucose[MeSH Terms]) AND Monitoring, Physiologic”[Mesh])) OR blood specimen collection[MeSH Terms] OR "blood specimen collection" [Title/Abstract] OR "blood sample"[Title/Abstract]) OR (continuous AND glucose AND monitor*) OR CGM[tiab] OR “glucose control”)) AND (((hyperglycemia[MeSH Terms]) OR hypoglycemia[MeSH Terms]) OR glycemia[Title/Abstract] OR glycaemia[Title/Abstract] OR “glycemic levels”[Title/Abstract] OR Hypoglyc* OR Hyperglyc*[Title/Abstract]))))))))))

*Delayed cord clamping or cord milking vs immediate clamping*

(((((((Infant, very low birth weight[Mesh] OR Infant, premature[Mesh] OR premature[Title/Abstract] OR prematurity[Title/Abstract] OR preterm[Title/Abstract] OR "pre term”[Title/Abstract] OR "low birth weight” [Title/Abstract] OR "low birthweight” [Title/Abstract] OR “very low birth weight” OR VLBW[Title/Abstract] OR LBW[Title/Abstract] OR “extremely low birth weight” OR neonat*[Title/Abstract])) AND ((randomized controlled trial [pt] OR controlled clinical trial [pt] OR randomized [tiab] OR randomised [tiab] OR randomly [tiab] OR trial [tiab] OR groups [tiab]) NOT (animals [mh] NOT humans [mh])))) ((((Delay*[Title/Abstract] OR defer*[Title/Abstract] OR late[Title/Abstract] OR early[Title/Abstract] OR immediate[Title/Abstract]))) AND ((umbilical cord[MeSH Terms]) OR (cord[Title/Abstract] OR "umbilical cord"[Title/Abstract]))) AND ((clamping[Title/Abstract] OR milking[Title/Abstract] OR stripping[Title/Abstract] OR “cord milking”[Title/Abstract] OR “cord clamping”[Title/Abstract] OR “cord stripping”[Title/Abstract] OR “blood sampling”[Title/abstract] OR “blood sample”[Title/abstract] OR “blood specimen collection”[Title/abstract] OR Blood specimen collection[Mesh]))

*Placenta or cord sample vs blood sample from preterm infant, search 20200211*

(((((Infant, very low birth weight[Mesh] OR Infant, premature[Mesh] OR premature[Title/Abstract] OR prematurity[Title/Abstract] OR preterm[Title/Abstract] OR "pre term”[Title/Abstract] OR "low birth weight” [Title/Abstract] OR "low birthweight” [Title/Abstract] OR “very low birth weight” OR VLBW[Title/Abstract] OR LBW[Title/Abstract] OR “extremely low birth weight” OR neonat*[Title/Abstract]) AND ((randomized controlled trial [pt] OR controlled clinical trial [pt] OR randomized [tiab] OR randomised [tiab] OR randomly [tiab] OR trial [tiab] OR groups [tiab]) NOT (animals [mh] NOT humans [mh]))) AND ((“blood sampling”[Title/abstract] OR “blood sample”[Title/abstract] OR “blood specimen collection”[Title/abstract] OR Blood specimen collection[Mesh]) OR ((umbilical cord[MeSH Terms] OR cord[Title/Abstract] OR "umbilical cord"[Title/Abstract]) AND (“blood sampling”[Title/abstract] OR “blood sample”[Title/abstract] OR “blood specimen collection”[Title/abstract] OR Blood specimen collection[Mesh]) OR (Placenta[Mesh] AND Blood[Mesh]) OR “placental serum”[Title/abstract] OR “placental blood”[Title/abstract] OR “placenta specimen”[Title/abstract])))))

## **Embase (Elsevier)**

*Micromethods and Reintroducing blood*

'very low birth weight'/exp OR 'prematurity'/exp OR premature:ab,ti OR prematurity:ab,ti OR preterm:ab,ti OR 'pre term':ab,ti OR 'low birth weight':ab,ti OR 'low birthweight':ab,ti OR 'very low birth weight':ab,ti OR vlbw:ab,ti OR lbw:ab,ti OR 'extremely low birth weight':ab,ti OR neonat*:ab,ti AND 'randomized controlled trial'/exp OR 'randomized controlled trial' OR (randomized AND controlled AND ('trial'/exp OR trial)) OR 'controlled clinical trial'/exp OR 'controlled clinical trial' OR (controlled AND ('clinical'/exp OR clinical) AND ('trial'/exp OR trial)) OR randomized OR randoised OR 'clinical trials as topic'/exp OR 'clinical trials as topic' OR (('clinical'/exp OR clinical) AND trials AND as AND topic) OR randomly OR 'trial'/exp OR trial OR 'clinical trial'/exp OR 'clinical trial' OR (('clinical'/exp OR clinical) AND ('trial'/exp OR trial))) NOT (('human'/exp OR human) NOT ('animal'/exp OR animal)) AND 'blood sampling'/exp OR 'blood sampling device'/exp OR 'point of care system'/exp OR 'phlebotomy'/exp OR 'blood specimen collection':ab,ti OR 'blood sampling':ab,ti OR 'blood conservation':ab,ti OR 'blood testing':ab,ti OR 're-introducing blood':ab,ti OR 'reintroducing blood':ab,ti OR 'dried blood spot':ab,ti OR phlebotomy:ab,ti OR (('point-of-care':ab,ti OR 'point of care':ab,ti OR poc:ab,ti) AND (technology:ab,ti OR device:ab,ti OR monitor*:ab,ti OR measur*:ab,ti))

*Devices to monitor CO2 levels transcutaneous vs blood sampling*

'very low birth weight'/exp OR 'prematurity'/exp OR premature:ab,ti OR prematurity:ab,ti OR preterm:ab,ti OR 'pre term':ab,ti OR 'low birth weight':ab,ti OR 'low birthweight':ab,ti OR 'very low birth weight':ab,ti OR vlbw:ab,ti OR lbw:ab,ti OR 'extremely low birth weight':ab,ti OR neonat*:ab,ti AND 'randomized controlled trial'/exp OR 'randomized controlled trial' OR (randomized AND controlled AND ('trial'/exp OR trial)) OR 'controlled clinical trial'/exp OR 'controlled clinical trial' OR (controlled AND ('clinical'/exp OR clinical) AND ('trial'/exp OR trial)) OR randomized OR randoised OR 'clinical trials as topic'/exp OR 'clinical trials as topic' OR (('clinical'/exp OR clinical) AND trials AND as AND topic) OR randomly OR 'trial'/exp OR trial OR 'clinical trial'/exp OR 'clinical trial' OR (('clinical'/exp OR clinical) AND ('trial'/exp OR trial))) NOT (('human'/exp OR human) NOT ('animal'/exp OR animal)) AND 'blood sampling'/exp OR 'blood gas analysis'/exp OR 'transcutaneous oxygen monitoring'/exp OR 'oximetry'/exp OR 'blood analysis'/exp OR (('blood specimen collection':ab,ti OR 'blood sampling':ab,ti OR transcutaneous:ab,ti OR monitor*or:ab,ti) AND ('point-of-care':ab,ti OR 'point of care':ab,ti OR poc:ab,ti) AND (technology:ab,ti OR device:ab,ti OR monitor*:ab,ti OR measur*:ab,ti))) AND ('carbon dioxide blood level'/exp OR 'carbon dioxide'/exp)

*Devices to monitor O2 levels transcutaneous or intraarterial vs blood sampling*

'very low birth weight'/exp OR 'prematurity'/exp OR premature:ab,ti OR prematurity:ab,ti OR preterm:ab,ti OR 'pre term':ab,ti OR 'low birth weight':ab,ti OR 'low birthweight':ab,ti OR 'very low birth weight':ab,ti OR vlbw:ab,ti OR lbw:ab,ti OR 'extremely low birth weight':ab,ti OR neonat*:ab,ti AND 'randomized controlled trial'/exp OR 'randomized controlled trial' OR (randomized AND controlled AND ('trial'/exp OR trial)) OR 'controlled clinical trial'/exp OR 'controlled clinical trial' OR (controlled AND ('clinical'/exp OR clinical) AND ('trial'/exp OR trial)) OR randomized OR randoised OR 'clinical trials as topic'/exp OR 'clinical trials as topic' OR (('clinical'/exp OR clinical) AND trials AND as AND topic) OR randomly OR 'trial'/exp OR trial OR 'clinical trial'/exp OR 'clinical trial' OR (('clinical'/exp OR clinical) AND ('trial'/exp OR trial))) NOT (('human'/exp OR human) NOT ('animal'/exp OR animal)) AND ('blood sampling'/exp OR 'blood gas analysis'/exp OR 'transcutaneous oxygen monitoring'/exp OR 'oximetry'/exp OR 'blood analysis'/exp OR (('blood specimen collection':ab,ti OR 'blood sampling':ab,ti OR transcutaneous:ab,ti OR monitor*or:ab,ti) AND ('point-of-care':ab,ti OR 'point of care':ab,ti OR poc:ab,ti) AND (technology:ab,ti OR device:ab,ti OR monitor*:ab,ti OR measur*:ab,ti)) OR 'intra arterial':ab,ti OR 'intraarterial or intra arterial':ab,ti OR 'arterial line':ab,ti OR 'in-line':ab,ti OR 'end-tidal':ab,ti OR 'end tidal':ab,ti) AND ('oxygen in blood'/exp OR 'oxygen'/exp)
*Devices to monitor glucose levels subcutaneous vs blood sampling*

'very low birth weight'/exp OR 'prematurity'/exp OR premature:ab,ti OR prematurity:ab,ti OR preterm:ab,ti OR 'pre term':ab,ti OR 'low birth weight':ab,ti OR 'low birthweight':ab,ti OR 'very low birth weight':ab,ti OR vlbw:ab,ti OR lbw:ab,ti OR 'extremely low birth weight':ab,ti OR neonat*:ab,ti AND 'randomized controlled trial'/exp OR 'randomized controlled trial' OR (randomized AND controlled AND ('trial'/exp OR trial)) OR 'controlled clinical trial'/exp OR 'controlled clinical trial' OR (controlled AND ('clinical'/exp OR clinical) AND ('trial'/exp OR trial)) OR randomized OR randoised OR 'clinical trials as topic'/exp OR 'clinical trials as topic' OR (('clinical'/exp OR clinical) AND trials AND as AND topic) OR randomly OR 'trial'/exp OR trial OR 'clinical trial'/exp OR 'clinical trial' OR (('clinical'/exp OR clinical) AND ('trial'/exp OR trial))) NOT (('human'/exp OR human) NOT ('animal'/exp OR animal)) AND 'blood glucose monitoring'/exp OR 'blood sampling'/exp OR (continuous:ab,ti AND glucose:ab,ti AND monitor*:ab,ti) OR cgm:ab,ti OR 'glucose control':ab,ti AND 'hypoglycemia'/exp OR 'hyperglycemia'/exp OR glycemia:ab,ti OR glycaemia:ab,ti OR 'glycemic levels':ab,ti OR hypoglyc*:ab,ti OR hyperglyc*:ab,ti

*Delayed cord clamping or cord milking vs immediate clamping, rev 200210*
('cord clamping'/exp OR 'umbilical cord'/exp OR 'umbilical cord':ab,ti OR cord:ab,ti) AND ((clamping:ab,ti OR milking:ab,ti OR stripping:ab,ti OR 'cord clamping':ab,ti OR 'cord milking':ab,ti OR 'cord stripping':ab,ti) AND (delay*:ab,ti OR deferr*:ab,ti OR late:ab,ti OR early:ab,ti OR immediate:ab,ti) OR 'blood sampling'/exp OR 'blood sample':ab,ti OR 'blood sampling':ab,ti OR 'blood specimen collection':ab,ti) AND ('randomized controlled trial'/exp OR 'randomized controlled trial' OR (randomized AND controlled AND ('trial'/exp OR trial)) OR 'controlled clinical trial'/exp OR 'controlled clinical trial' OR (controlled AND ('clinical'/exp OR clinical) AND ('trial'/exp OR trial)) OR randomized OR randoised OR 'clinical trials as topic'/exp OR 'clinical trials as topic' OR (('clinical'/exp OR clinical) AND trials AND as AND topic) OR randomly OR 'trial'/exp OR trial OR 'clinical trial'/exp OR 'clinical trial' OR (('clinical'/exp OR clinical) AND ('trial'/exp OR trial))) NOT (('human'/exp OR human) NOT ('animal'/exp OR animal))

*Placenta or cord sample vs blood sample from preterm infant, search 20200211*

('very low birth weight'/exp OR 'very low birth weight' OR 'prematurity'/exp OR 'prematurity' OR premature:ab,ti OR prematurity:ab,ti OR preterm:ab,ti OR 'pre term':ab,ti OR 'low birth weight':ab,ti OR 'low birthweight':ab,ti OR 'very low birth weight':ab,ti OR vlbw:ab,ti OR lbw:ab,ti OR 'extremely low birth weight':ab,ti OR neonat*:ab,ti) AND ('randomized controlled trial'/exp OR 'randomized controlled trial' OR (randomized AND controlled AND ('trial' OR 'trial'/exp OR trial)) OR 'controlled clinical trial'/exp OR 'controlled clinical trial' OR (controlled AND ('clinical' OR 'clinical'/exp OR clinical) AND ('trial' OR 'trial'/exp OR trial)) OR randomized OR randomised OR 'clinical trials as topic'/exp OR 'clinical trials as topic' OR (('clinical' OR 'clinical'/exp OR clinical) AND trials AND as AND topic) OR randomly OR 'trial' OR 'trial'/exp OR trial OR 'clinical trial'/exp OR 'clinical trial' OR (('clinical' OR 'clinical'/exp OR clinical) AND ('trial' OR 'trial'/exp OR trial))) NOT (('human' OR 'human'/exp OR human) NOT ('animal' OR 'animal'/exp OR animal)) AND (('blood sample':ab,ti OR 'blood sampling':ab,ti OR 'blood specimen collection':ab,ti OR 'blood sampling'/exp OR 'blood sampling') OR (('umbilical cord':ab,ti OR cord:ab,ti OR 'umbilical cord'/exp OR 'umbilical cord') AND ('blood sample':ab,ti OR 'blood sampling':ab,ti OR 'blood specimen collection':ab,ti OR 'blood sampling'/exp OR 'blood sampling') OR 'placental serum':ab,ti OR 'placental blood':ab,ti OR 'placenta specimen':ab,ti))

## **CINAHL Complete (Ebsco)**

*Micromethods and Reintroducing blood*

(Infant, premature [MH] OR Infant, very low birth weight [MH] OR premature[TIAB] OR prematurity[TIAB] OR preterm[TIAB] OR "pre term"[TIAB] OR "low birth weight"[TIAB] OR "low birthweight"[TIAB] OR “extremely low birth weight” OR “very low birth weight”[TIAB] OR VLBW[TIAB] OR LBW[TIAB] OR infan*[TIAB] OR neonat*[TIAB]) AND (randomized controlled trial OR controlled clinical trial OR randomized OR randomized OR clinical trials as topic OR randomly OR trial OR PT clinical trial) AND (MH Blood specimen collection OR MH phlebotomy OR TI ( "blood specimen collection" OR "blood sampling" OR "blood conservation" OR “blood testing” OR "re-introducing blood" OR “reintroducing blood” OR "dried blood spot" OR phlebotomy OR “point-of-care”OR “point of care”OR POC AND ((technology OR device OR monitor* OR measur*)) ) OR AB ( "blood specimen collection" OR "blood sampling" OR "blood conservation" OR “blood testing” OR "re-introducing blood" OR “reintroducing blood” OR "dried blood spot" OR phlebotomy OR “point-of-care”OR “point of care”OR POC AND ((technology OR device OR monitor* OR measur*)))

*Devices to monitor CO2 levels transcutaneous vs blood sampling*

(Infant, premature [MH] OR Infant, very low birth weight [MH] OR premature[TIAB] OR prematurity[TIAB] OR preterm[TIAB] OR "pre term"[TIAB] OR "low birth weight"[TIAB] OR "low birthweight"[TIAB] OR “extremely low birth weight” OR “very low birth weight”[TIAB] OR VLBW[TIAB] OR LBW[TIAB] OR infan*[TIAB] OR neonat*[TIAB]) AND (randomized controlled trial OR controlled clinical trial OR randomized OR randomized OR clinical trials as topic OR randomly OR trial OR PT clinical trial) AND (TI "blood specimen collection" OR "blood sampling" OR transcutaneous OR monitor* OR (“point-of-care” OR “point of care” OR POC AND (technology OR device OR monitor* OR measur*) OR AB "blood specimen collection" OR "blood sampling" OR transcutaneous OR monitor* OR (“point-of-care” OR “point of care” OR POC AND (technology OR device OR monitor* OR measur*) OR MH blood specimen collection OR MH blood gas analysis OR MH blood gas monitoring, transcutaneous OR MH oximetry OR MH blood chemistry) AND (MH carbon dioxide)

*Devices to monitor O2 levels transcutaneous or intra-arterial vs blood sampling*

(Infant, premature [MH] OR Infant, very low birth weight [MH] OR premature[TIAB] OR prematurity[TIAB] OR preterm[TIAB] OR "pre term"[TIAB] OR "low birth weight"[TIAB] OR "low birthweight"[TIAB] OR “extremely low birth weight” OR “very low birth weight”[TIAB] OR VLBW[TIAB] OR LBW[TIAB] OR infan*[TIAB] OR neonat*[TIAB]) AND (randomized controlled trial OR controlled clinical trial OR randomized OR randomized OR clinical trials as topic OR randomly OR trial OR PT clinical trial) AND (TI "blood specimen collection" OR "blood sampling" OR transcutaneous OR monitor* OR Intra-arterial OR intraarterial OR ” intra arterial” OR “arterial line” OR “in-line” OR “end-tidal” OR “end tidal” OR (“point-of-care” OR “point of care” OR POC AND (technology OR device OR monitor* OR measur*) OR AB "blood specimen collection" OR "blood sampling" OR transcutaneous OR monitor* OR Intra-arterial OR intraarterial OR ” intra arterial” OR “arterial line” OR “in-line” OR “end-tidal” OR “end tidal” OR (“point-of-care” OR “point of care” OR POC AND (technology OR device OR monitor* OR measur*) OR MH blood specimen collection OR MH blood gas analysis OR MH blood gas monitoring, transcutaneous OR MH oximetry OR MH blood chemistry) AND (MH oxygen)

*Devices to monitor glucose levels subcutaneous vs blood sampling*

(Infant, premature [MH] OR Infant, very low birth weight [MH] OR premature[TIAB] OR prematurity[TIAB] OR preterm[TIAB] OR "pre term"[TIAB] OR "low birth weight"[TIAB] OR "low birthweight"[TIAB] OR “extremely low birth weight” OR “very low birth weight”[TIAB] OR VLBW[TIAB] OR LBW[TIAB] OR infan*[TIAB] OR neonat*[TIAB]) AND (randomized controlled trial OR controlled clinical trial OR randomized OR randomized OR clinical trials as topic OR randomly OR trial OR PT clinical trial) AND MH blood glucose monitoring OR MH blood specimen collection OR TI ( (continuous AND glucose AND monitor*) OR (CGM OR “glucose control”) ) OR AB ( (continuous AND glucose AND monitor*) OR (CGM OR “glucose control”) ) AND
(MH hyperglycemia OR MH hypoglycemia OR TI glycemia OR glycaemia OR “glycemic levels” OR Hypoglyc* OR Hyperglyc* OR AB glycemia OR glycaemia OR “glycemic levels” OR Hypoglyc* OR Hyperglyc*)

*Delayed cord clamping or cord milking vs immediate clamping*
(TI ( clamping OR milking OR stripping OR "cord clamping" OR "cord stripping" OR "cord milking" ) OR AB ( clamping OR milking OR stripping OR "cord clamping" OR "cord stripping" OR "cord milking")  OR (MH umbilical cord OR TI umbilical cord OR AB umbilical cord ) OR TI cord OR AB cord) AND (TI ( delay* OR deferr* OR immediate OR late OR early ) OR AB ( delay* OR deferr* OR immediate OR late OR early ) OR (MH blood specimen collection OR TI ( blood sampling OR blood sample OR blood specimen collection ) OR AB ( blood sampling OR blood sample OR blood specimen collection ))

*Placenta or cord sample vs blood sample from preterm infant, search 20200211*

(Infant, premature [MH] OR Infant, very low birth weight [MH] OR premature[TIAB] OR prematurity[TIAB] OR preterm[TIAB] OR "pre term"[TIAB] OR "low birth weight"[TIAB] OR "low birthweight"[TIAB] OR “extremely low birth weight” OR “very low birth weight”[TIAB] OR VLBW[TIAB] OR LBW[TIAB] OR infan*[TIAB] OR neonat*[TIAB]) AND (randomized controlled trial OR controlled clinical trial OR randomized OR randomized OR clinical trials as topic OR randomly OR trial OR PT clinical trial) AND MH ((Blood specimen collection) OR TI ( blood sample OR blood sampling OR blood specimen collection OR placental blood OR placenta specimen OR placental serum ) OR AB ( blood sample OR blood sampling OR blood specimen collection OR placental blood OR placenta specimen OR placental serum ) OR MH umbilical cord OR TI ( umbilical cord OR cord ) OR AB ( umbilical cord OR cord ))

## **CENTRAL**

*Micromethods and Reintroducing blood*

MeSH descriptor: [Infant, Premature] explode all trees OR MeSH descriptor: [Infant, Very Low Birth Weight] explode all trees OR premature OR prematurity OR preterm OR pre term OR low birth weight OR low birthweight OR very low birth weight OR VLBW OR LBW OR extremely low birth weight OR neonat*:ti,ab,kw AND (randomized controlled trial OR controlled clinical trial OR randomized OR randomised OR randomly OR trial OR groups NOT (animals NOT humans)) AND MeSH descriptor: [Point-of-Care Systems] explode all trees OR MeSH descriptor: [Phlebotomy] explode all trees OR MeSH descriptor: [Point-of-Care Systems] explode all trees OR blood specimen collectionOR blood sampling OR blood conservation OR blood testing OR re-introducing blood OR reintroducing blood OR dried blood spot OR phlebotomy OR( (“point-of-care” OR “point of care” OR POC) AND (technology OR device OR monitor* OR measur*)):ti,ab,kw AND (reduc* OR decreas* OR minimiz*) AND (blood loss OR transfusion OR anemia):ti,ab,kw

MeSH descriptor: [Infant, Premature] explode all trees OR MeSH descriptor: [Infant, Very Low Birth Weight] explode all trees OR premature OR prematurity OR preterm OR pre term OR low birth weight OR low birthweight OR very low birth weight OR VLBW OR LBW OR extremely low birth weight OR neonat*:ti,ab,kw AND (randomized controlled trial OR controlled clinical trial OR randomized OR randomised OR randomly OR trial OR groups NOT (animals NOT humans))

*Devices to monitor CO2 levels transcutaneous vs blood sampling*

MeSH descriptor: [Infant, Premature] explode all trees OR MeSH descriptor: [Infant, Very Low Birth Weight] explode all trees OR premature OR prematurity OR preterm OR pre term OR low birth weight OR low birthweight OR very low birth weight OR VLBW OR LBW OR extremely low birth weight OR neonat*:ti,ab,kw AND (randomized controlled trial OR controlled clinical trial OR randomized OR randomised OR randomly OR trial OR groups NOT (animals NOT humans)) AND MeSH descriptor: [Blood Chemical Analysis] explode all trees OR MeSH descriptor: [Blood Gas Analysis] explode all trees OR MeSH descriptor: [Blood Gas Monitoring, Transcutaneous] explode all trees OR MeSH descriptor: [Oximetry] explode all trees OR blood specimen collection OR blood sampling OR transcutaneous OR monitor* OR ((point-of-care OR point of care OR POC) AND (technology OR device OR monitor* OR measur*)):ti,ab,kw AND (MeSH descriptor: [Carbon dioxide] explode all trees OR MeSH descriptor: [Carbon Dioxide] explode all trees and with qualifier(s): [blood - BL])

*Devices to monitor O2 levels transcutaneous or intra-arterial vs blood sampling*

MeSH descriptor: [Infant, Premature] explode all trees OR MeSH descriptor: [Infant, Very Low Birth Weight] explode all trees OR premature OR prematurity OR preterm OR pre term OR low birth weight OR low birthweight OR very low birth weight OR VLBW OR LBW OR extremely low birth weight OR neonat*:ti,ab,kw AND (randomized controlled trial OR controlled clinical trial OR randomized OR randomised OR randomly OR trial OR groups NOT (animals NOT humans)) AND MeSH descriptor: [Blood Chemical Analysis] explode all trees OR MeSH descriptor: [Blood Gas Analysis] explode all trees OR MeSH descriptor: [Blood Gas Monitoring, Transcutaneous] explode all trees OR MeSH descriptor: [Oximetry] explode all trees OR blood specimen collection OR blood sampling OR transcutaneous OR monitor* OR Intra-arterial OR intraarterial OR intra arterial OR arterial line OR in-line OR end-tidal OR end tidal ((point-of-care OR point of care OR POC) AND (technology OR device OR monitor* OR measur*)):ti,ab,kw AND (MeSH descriptor: [Oxygen] explode all trees and with qualifier(s): [blood - BL] OR MeSH descriptor: [Oxygen] explode all trees)

*Devices to monitor glucose levels subcutaneous vs blood sampling*

MeSH descriptor: [Infant, Premature] explode all trees OR MeSH descriptor: [Infant, Very Low Birth Weight] explode all trees OR premature OR prematurity OR preterm OR pre term OR low birth weight OR low birthweight OR very low birth weight OR VLBW OR LBW OR extremely low birth weight OR neonat*:ti,ab,kw AND (randomized controlled trial OR controlled clinical trial OR randomized OR randomised OR randomly OR trial OR groups NOT (animals NOT humans)) AND (MeSH descriptor: [Monitoring, Physiologic] explode all trees AND MeSH descriptor: [Blood Glucose] explode all trees) OR MeSH descriptor: [Blood Specimen Collection] explode all trees AND (continuous AND glucose AND monitor*) OR CGM OR glucose control:ti,ab,kw

AND (MeSH descriptor: [Hypoglycemia] explode all trees OR MeSH descriptor: [Hyperglycemia] explode all trees OR glycemia OR glycaemia OR glycemic levels OR Hypoglyc* OR Hyperglyc*):ti,ab,kw

*Delayed cord clamping or cord milking vs immediate clamping*

randomized controlled trial OR controlled clinical trial OR randomized OR randomised OR randomly OR trial OR groups NOT (animals NOT humans) AND (umbilical cord OR cord:ti,ab,kw OR umbilical cord OR cord:ti,ab,kw) AND ((delay* OR deferr* OR immediate OR late OR early AND clamping OR milking OR stripping:ti,ab,kw) OR(MeSH descriptor: [Blood Specimen Collection] explode all trees OR blood specimen collection OR blood sample OR blood sampling:ti,ab,kw))

*Placenta or cord sample vs blood sample from preterm infant, search 20200211*

MeSH descriptor: [Infant, Premature] explode all trees OR MeSH descriptor: [Infant, Very Low Birth Weight] explode all trees OR premature OR prematurity OR preterm OR pre term OR low birth weight OR low birthweight OR very low birth weight OR VLBW OR LBW OR extremely low birth weight OR neonat*:ti,ab,kw AND (randomized controlled trial OR controlled clinical trial OR randomized OR randomised OR randomly OR trial OR groups NOT (animals NOT humans)) AND (MeSH descriptor: [Blood Specimen Collection] explode all trees OR (blood specimen collection OR blood sample OR blood sampling:ti,ab,kw) AND (cord OR umbilical cord:ti,ab,kw OR MeSH descriptor: [Umbilical Cord] explode all trees)) OR (MeSH descriptor: [Placenta] explode all trees AND MeSH descriptor: [Blood] explode all trees) OR (placental serum OR placental blood OR placenta specimen:ti,ab,kw OR placental serum OR placental blood OR placenta specimen:ti,ab,kw)

# **Clinicaltrials.gov**

Advanced search, Other terms: Premature infant AND blood

*Additional search 20200211*

((preterm OR premature) AND (Infant) OR (neonate)) AND ((blood sample OR blood sampling OR blood specimen collection) AND (umbilical cord)) OR (placental specimen OR placental blood OR placenta serum)

# International Clinical Trial Registry Platform, ICTRP

premature OR prematurity OR preterm OR pre term OR low birth weight OR low birthweight OR VLBW OR LBW OR infan* OR neonat* AND (blood sample OR blood specimen collection OR blood loss) [Title field]

*Additional search 20200211*

(premature OR prematurity OR preterm OR pre term OR low birth weight OR low birthweight OR VLBW OR LBW OR infan* OR neonat*) AND (((blood sample OR blood sampling OR blood specimen collection) AND (umbilical cord)) OR (placental specimen OR placental blood OR placenta serum)) [Title field]

1. **LILACS**

*Strategy 1 -* *Micromethods and Reintroducing blood (1AND2AND3AND4 = 29 articles retrieved)*

1. MH: ("Infant, Very Low Birth Weight") OR ("Infant, Very-Low-Birth-Weight") OR ("Infants, Very-Low-Birth-Weight") OR ("Very Low Birth Weight") OR ("Very Low Birth Weight Infant") OR ("Very-Low-Birth-Weight Infant") OR ("Very-Low-Birth-Weight Infants") OR ("Recién Nacido de muy Bajo Peso") OR ("Recém-Nascido de muito Baixo Peso") OR ("Infant, Premature") OR ("Infant, Preterm") OR ("Infants, Premature") OR ("Infants, Preterm") OR ("Neonatal Prematurity") OR ("Premature Infant") OR ("Premature Infants") OR ("Prematurity, Neonatal") OR ("Preterm Infant") OR ("Preterm Infants") OR ("Recien Nacido Prematuro") OR ("Recém-Nascido Prematuro") OR MS:M01.060.703.520.460.600$ OR MH:M01.060.703.520.520$
2. MH: ("Randomized Controlled Trial") OR ("Ensayo Clínico Controlado Aleatorio") OR ("Ensaio Clínico Controlado Aleatório") OR MH:V03.175.250.500.500$
3. MH: ("Blood Specimen Collection") OR ("Recolección de Muestras de Sangre") OR ("Coleta de Amostras Sanguíneas") OR ("Blood Specimen Collections") OR ("Collection, Blood Specimen") OR ("Collections, Blood Specimen") OR ("Specimen Collection, Blood") OR ("Specimen Collections, Blood") OR ("Monitoring, Physiologic") OR ("Monitoreo Fisiológico”) OR ("Monitorização Fisiológica”) OR ("Monitoring, Patient”) OR ("Monitoring, Physiological”) OR ("Patient Monitoring") OR ("Physiologic Monitoring”) OR ("Physiological Monitoring”) OR MH:E01.370.225.998.110$ OR MH:E04.665.150$ OR MH:E05.200.998.110$ OR MH:E01.370.520$
4. MH: ("Point-of-Care Systems”) OR (“Sistemas de Atención de Punto”) OR ("Sistemas Automatizados de Assistência Junto ao Leito”) OR ("Bedside Computing”) OR ("Bedside Technologies") OR ("Bedside Technology") OR ("Computing, Bedside") OR ("Point of Care") OR ("Point of Care Systems") OR ("Point of Care Technology") OR ("Point-of-Care") OR ("Point-of-Care System") OR ("Systems, Point-of-Care") OR ("Technologies, Bedside") OR ("Technology, Bedside”) OR Phlebotomy OR Flebotomía OR Flebotomia OR Phlebotomies OR Venesection OR Venesections OR Venipuncture OR Venipunctures OR MH:N04.452.442.452.680 OR MH:N04.452.515.360.652$ OR MH:N04.590.874$ OR MH:E01.370.225.998.110.625$ OR MH:E02.800.558$ OR MH:E04.665.150.625$ OR MH:E05.200.998.110.625$

*Strategy 2 - Devices to monitor CO2 levels transcutaneous versus blood sampling (1 AND 2 AND 3 AND 4 = 23 articles retrieved)*

1. MH: ("Infant, Very Low Birth Weight") OR ("Infant, Very-Low-Birth-Weight") OR ("Infants, Very-Low-Birth-Weight") OR ("Very Low Birth Weight") OR ("Very Low Birth Weight Infant") OR ("Very-Low-Birth-Weight Infant") OR ("Very-Low-Birth-Weight Infants") OR ("Recién Nacido de muy Bajo Peso") OR ("Recém-Nascido de muito Baixo Peso") OR ("Infant, Premature") OR ("Infant, Preterm") OR ("Infants, Premature") OR ("Infants, Preterm") OR ("Neonatal Prematurity") OR ("Premature Infant") OR ("Premature Infants") OR ("Prematurity, Neonatal") OR ("Preterm Infant") OR ("Preterm Infants") OR ("Recien Nacido Prematuro") OR ("Recém-Nascido Prematuro") OR MS:M01.060.703.520.460.600$ OR MH:M01.060.703.520.520$
2. MH: ("Randomized Controlled Trial") OR ("Ensayo Clínico Controlado Aleatorio") OR ("Ensaio Clínico Controlado Aleatório") OR MH:V03.175.250.500.500$
3. MH: ("Blood Specimen Collection") OR ("Recolección de Muestras de Sangre") OR ("Coleta de Amostras Sanguíneas") OR ("Blood Specimen Collections") OR ("Collection, Blood Specimen") OR ("Collections, Blood Specimen") OR ("Specimen Collection, Blood") OR ("Specimen Collections, Blood") OR ("Blood Gas Analysis") OR ("Análisis de los Gases de la Sangre") OR (Gasometria) OR ("Analyses, Blood Gas") OR ("Analysis, Blood Gas") OR ("Blood Gas Analyses") OR ("Gas Analyses, Blood") OR ("Gas Analysis, Blood") OR ("Blood Gas Monitoring, Transcutaneous") OR ("Monitoreo de Gas Sanguíneo Transcutáneo") OR ("Monitorização Transcutânea dos Gases Sanguíneos") OR ("Capnometries, Transcutaneous") OR ("Capnometry, Transcutaneous") OR ("Carbon Dioxide Partial Pressure Determination, Transcutaneous") OR ("Cutaneous Oximetries") OR ("Cutaneous Oximetry") OR ("Oximetries, Cutaneous") OR ("Oximetries, Transcutaneous") OR ("Oximetry, Cutaneous") OR ("Oximetry, Transcutaneous") OR ("Oxygen Partial Pressure Determination, Transcutaneous") OR ("PtcO2") OR ("TcPCO2") OR ("Transcutaneous Blood Gas Monitoring") OR ("Transcutaneous Capnometries") OR ("Transcutaneous Capnometry") OR ("Transcutaneous Oximetries") OR ("Transcutaneous Oximetry") OR (Oximetry) OR (Oximetría) OR (Oximetria) OR (Oximetries) OR ("Oximetries, Pulse") OR ("Oximetry, Pulse") OR ("Pulse Oximetries") OR ("Pulse Oximetry") OR ("Blood Chemical Analysis") OR ("Análisis Químico de la Sangre") OR ("Análise Química do Sangue") OR ("Analyses, Blood Chemical") OR ("Analysis, Blood Chemical") OR ("Blood Chemical Analyses") OR ("Chemical Analyses, Blood") OR ("Chemical Analysis, Blood") ("Monitoring, Physiologic") OR ("Monitoreo Fisiológico”) OR ("Monitorização Fisiológica”) OR ("Monitoring, Patient”) OR ("Monitoring, Physiological”) OR ("Patient Monitoring") OR ("Physiologic Monitoring”) OR ("Physiological Monitoring”) OR MH:E01.370.225.998.110$ OR MH:E04.665.150$ OR MH:E05.200.998.110$ OR MH:E01.370.520$ OR MH:E01.370.225.124.100.100$ OR MH:E01.370.386.700.100$ OR MH:E05.200.124.100.100$ OR MH:E01.370.225.124.100.100.600.100$ OR MH:E01.370.370.380.600.100$ OR MH:E01.370.386.700.100.600.100$ OR MH:E05.200.124.100.100.600.100$ OR MH:E01.370.225.124.100.100.600$ OR MH:E01.370.370.380.600$ OR MH:E01.370.386.700.100.600$ OR MH:E05.200.124.100.100.600$ OR MH:E01.370.225.124.100$ OR MH:E05.200.124.100$
4. MH: ("Carbon Dioxide") OR ("Dióxido de Carbono") OR ("Díoxido de Carbono") OR ("Anhydride, Carbonic") OR ("Carbonic Anhydride") OR ("Dioxide, Carbon") OR MH:D01.200.200$ OR MH:D01.362.150$ OR MH:D01.650.550.200$ OR MH:SP4.011.087.723.449.300.296$ OR MH:SP4.011.097.039.779.510$ OR MH:SP4.041.432.533.010.030.010.010$ OR MH:SP8.473.654.412.052.044$

*Strategy 3 - Devices to monitor O2 levels transcutaneous or intra-arterial versus blood sampling (1 AND 2 AND 3 AND 4 = 91 articles retrieved)*

1. MH: ("Infant, Very Low Birth Weight") OR ("Infant, Very-Low-Birth-Weight") OR ("Infants, Very-Low-Birth-Weight") OR ("Very Low Birth Weight") OR ("Very Low Birth Weight Infant") OR ("Very-Low-Birth-Weight Infant") OR ("Very-Low-Birth-Weight Infants") OR ("Recién Nacido de muy Bajo Peso") OR ("Recém-Nascido de muito Baixo Peso") OR ("Infant, Premature") OR ("Infant, Preterm") OR ("Infants, Premature") OR ("Infants, Preterm") OR ("Neonatal Prematurity") OR ("Premature Infant") OR ("Premature Infants") OR ("Prematurity, Neonatal") OR ("Preterm Infant") OR ("Preterm Infants") OR ("Recien Nacido Prematuro") OR ("Recém-Nascido Prematuro") OR MS:M01.060.703.520.460.600$ OR MH:M01.060.703.520.520$
2. MH: ("Randomized Controlled Trial") OR ("Ensayo Clínico Controlado Aleatorio") OR ("Ensaio Clínico Controlado Aleatório") OR MH:V03.175.250.500.500$
3. MH: ("Blood Specimen Collection") OR ("Recolección de Muestras de Sangre") OR ("Coleta de Amostras Sanguíneas") OR ("Blood Specimen Collections") OR ("Collection, Blood Specimen") OR ("Collections, Blood Specimen") OR ("Specimen Collection, Blood") OR ("Specimen Collections, Blood") OR ("Blood Gas Analysis") OR ("Análisis de los Gases de la Sangre") OR (Gasometria) OR ("Analyses, Blood Gas") OR ("Analysis, Blood Gas") OR ("Blood Gas Analyses") OR ("Gas Analyses, Blood") OR ("Gas Analysis, Blood") OR ("Blood Gas Monitoring, Transcutaneous") OR ("Monitoreo de Gas Sanguíneo Transcutáneo") OR ("Monitorização Transcutânea dos Gases Sanguíneos") OR ("Capnometries, Transcutaneous") OR ("Capnometry, Transcutaneous") OR ("Carbon Dioxide Partial Pressure Determination, Transcutaneous") OR ("Cutaneous Oximetries") OR ("Cutaneous Oximetry") OR ("Oximetries, Cutaneous") OR ("Oximetries, Transcutaneous") OR ("Oximetry, Cutaneous") OR ("Oximetry, Transcutaneous") OR ("Oxygen Partial Pressure Determination, Transcutaneous") OR ("PtcO2") OR ("TcPCO2") OR ("Transcutaneous Blood Gas Monitoring") OR ("Transcutaneous Capnometries") OR ("Transcutaneous Capnometry") OR ("Transcutaneous Oximetries") OR ("Transcutaneous Oximetry") OR (Oximetry) OR (Oximetría) OR (Oximetria) OR (Oximetries) OR ("Oximetries, Pulse") OR ("Oximetry, Pulse") OR ("Pulse Oximetries") OR ("Pulse Oximetry") OR ("Blood Chemical Analysis") OR ("Análisis Químico de la Sangre") OR ("Análise Química do Sangue") OR ("Analyses, Blood Chemical") OR ("Analysis, Blood Chemical") OR ("Blood Chemical Analyses") OR ("Chemical Analyses, Blood") OR ("Chemical Analysis, Blood") ("Monitoring, Physiologic") OR ("Monitoreo Fisiológico”) OR ("Monitorização Fisiológica”) OR ("Monitoring, Patient”) OR ("Monitoring, Physiological”) OR ("Patient Monitoring") OR ("Physiologic Monitoring”) OR ("Physiological Monitoring”) OR MH:E01.370.225.998.110$ OR MH:E04.665.150$ OR MH:E05.200.998.110$ OR MH:E01.370.520$ OR MH:E01.370.225.124.100.100$ OR MH:E01.370.386.700.100$ OR MH:E05.200.124.100.100$ OR MH:E01.370.225.124.100.100.600.100$ OR MH:E01.370.370.380.600.100$ OR MH:E01.370.386.700.100.600.100$ OR MH:E05.200.124.100.100.600.100$ OR MH:E01.370.225.124.100.100.600$ OR MH:E01.370.370.380.600$ OR MH:E01.370.386.700.100.600$ OR MH:E05.200.124.100.100.600$ OR MH:E01.370.225.124.100$ OR MH:E05.200.124.100$
4. MH: (Oxygen) OR (Oxígeno) OR (Oxigênio) OR (Dioxygen) OR ("Oxygen 16") OR ("Oxygen-16") OR MH:D01.268.185.550$ OR MH:D01.362.670$ OR MH:SP4.011.097.063.949$

*Strategy 4 - Devices to monitor glucose levels subcutaneous versus blood sampling (1 AND 2 AND 3 AND 4 AND 5 = 5 articles retrieved)*

1. MH: ("Infant, Very Low Birth Weight") OR ("Infant, Very-Low-Birth-Weight") OR ("Infants, Very-Low-Birth-Weight") OR ("Very Low Birth Weight") OR ("Very Low Birth Weight Infant") OR ("Very-Low-Birth-Weight Infant") OR ("Very-Low-Birth-Weight Infants") OR ("Recién Nacido de muy Bajo Peso") OR ("Recém-Nascido de muito Baixo Peso") OR ("Infant, Premature") OR ("Infant, Preterm") OR ("Infants, Premature") OR ("Infants, Preterm") OR ("Neonatal Prematurity") OR ("Premature Infant") OR ("Premature Infants") OR ("Prematurity, Neonatal") OR ("Preterm Infant") OR ("Preterm Infants") OR ("Recien Nacido Prematuro") OR ("Recém-Nascido Prematuro") OR MS:M01.060.703.520.460.600$ OR MH:M01.060.703.520.520$
2. MH: ("Randomized Controlled Trial") OR ("Ensayo Clínico Controlado Aleatorio") OR ("Ensaio Clínico Controlado Aleatório") OR MH:V03.175.250.500.500$
3. MH: ("Blood Specimen Collection") OR ("Recolección de Muestras de Sangre") OR ("Coleta de Amostras Sanguíneas") OR ("Blood Specimen Collections") OR ("Collection, Blood Specimen") OR ("Collections, Blood Specimen") OR ("Specimen Collection, Blood") OR ("Specimen Collections, Blood") OR ("Monitoring, Physiologic") OR ("Monitoreo Fisiológico”) OR ("Monitorização Fisiológica”) OR ("Monitoring, Patient”) OR ("Monitoring, Physiological”) OR ("Patient Monitoring") OR ("Physiologic Monitoring”) OR ("Physiological Monitoring”) OR MH:E01.370.225.998.110$ OR MH:E04.665.150$ OR MH:E05.200.998.110$ OR MH:E01.370.520$
4. MH: ("Blood glucose") OR (Glucemia) OR (Glicemia) OR ("Blood Sugar") OR ("Glucose, Blood") OR ("Sugar, Blood") OR MH:D09.947.875.359.448.500$
5. MH: (Hyperglycemia) OR (Hiperglucemia) OR (Hiperglicemia) OR ("Hyperglycemia, Postprandial") OR (Hyperglycemias) OR ("Hyperglycemias, Postprandial") OR ("Postprandial Hyperglycemia") OR ("Postprandial Hyperglycemias") OR (Hypoglycemia) OR (Hipoglucemia) OR (Hipoglicemia) OR ("Fasting Hypoglycemia") OR ("Hypoglycemia, Fasting") OR ("Hypoglycemia, Postabsorptive") OR ("Hypoglycemia, Postprandial") OR ("Hypoglycemia, Reactive") OR ("Postabsorptive Hypoglycemia") OR ("Postprandial Hypoglycemia") OR ("Reactive Hypoglycemia") OR MH:C18.452.394.952$ OR MH:C18.452.394.984$

*Strategy 5 - Devices to monitor glucose levels subcutaneous versus blood sampling (1 AND 2 AND 3 = 92 articles retrieved)*

1. MH: ("Infant, Very Low Birth Weight") OR ("Infant, Very-Low-Birth-Weight") OR ("Infants, Very-Low-Birth-Weight") OR ("Very Low Birth Weight") OR ("Very Low Birth Weight Infant") OR ("Very-Low-Birth-Weight Infant") OR ("Very-Low-Birth-Weight Infants") OR ("Recién Nacido de muy Bajo Peso") OR ("Recém-Nascido de muito Baixo Peso") OR ("Infant, Premature") OR ("Infant, Preterm") OR ("Infants, Premature") OR ("Infants, Preterm") OR ("Neonatal Prematurity") OR ("Premature Infant") OR ("Premature Infants") OR ("Prematurity, Neonatal") OR ("Preterm Infant") OR ("Preterm Infants") OR ("Recien Nacido Prematuro") OR ("Recém-Nascido Prematuro") OR MS:M01.060.703.520.460.600$ OR MH:M01.060.703.520.520$
2. MH: ("Randomized Controlled Trial") OR ("Ensayo Clínico Controlado Aleatorio") OR ("Ensaio Clínico Controlado Aleatório") OR MH:V03.175.250.500.500$
3. MH: ("Umbilical Cord") OR ("Cordón Umbilical") OR ("Cordão Umbilical") OR ("Cord, Umbilical") OR ("Cords, Umbilical") OR ("Umbilical Cords") OR MH:A16.378.693$
