# Supplementary material for: Interventions to minimize blood loss in very preterm infants—A systematic review and meta-analysis
Source: PLoS One. 2021 Feb 8;16(2):e0246353. doi: 10.1371/journal.pone.0246353 (PMC7870155; doi:10.1371/journal.pone.0246353)
Supplement: S2 File — (DOCX) [file pone.0246353.s003.docx]

# Characteristics and risk of bias of each included study

**Alan 2014**

| **Methods** | **Study design:** Randomized controlled trial  **Study grouping:** Parallel group |
| --- | --- |
| **Participants** | **Baseline Characteristics**  Cord milking   - *Gestational age (SD) (weeks)*: 28.4 (1.8) - *Birth weight (SD) (grams)*: 1103 (236) - *Number of patients at randomization*: 24 - *Number of patients at outcome*: 22, 19 (some outcomes)   Immediate cord clamping   - *Gestational age (SD) (weeks)*: 28.0 (1.9) - *Birth weight (SD) (grams)*: 1101 (262) - *Number of patients at randomization*: 24 - *Number of patients at outcome*: 22, 19 (some outcomes)   **Included criteria:** Gestational age <=32 weeks and estimated BW <=1500 g  **Excluded criteria:** suspected twin to twin transfusion syndrome or discordant twins; major congenital anomalies or chromosomal anomalies; vaginal bleeding due to placenta previa or abruption or placental tear; hemolytic disease of the fetus and newborn like Rhesus sensitization; intrauterine growth restriction; maternal gestational diabetes treated with insulin; hydrops fetalis; and refused parental consent  **Pretreatment:** Comparable baseline characteristics |
| **Interventions** | **Intervention Characteristics**  Cord milking   - *Description of procedure*: Infants were placed at the level of placenta in cesarean deliveries and below the level of placenta in vaginal deliveries in UCM group The umbilical cord was held at 25 to 30 cm distance from the baby and milked vigorously toward the umbilicus for 3 times at a speed of approximately 5 cm/seconds by the attending neonatologist before clamping. - *Time to cord clamping*: N/A   Immediate cord clamping   - *Description of procedure*: immediate cord clamping (< 10 s) - *Time to cord clamping*: 0-10 s |
| **Outcomes** | *Retinopathy of prematurity*   - **Outcome type**: Dichotomous Outcome   *IVH*   - **Outcome type**: Dichotomous Outcome   *Bronchopulmonary dysplasia/chronic lung disease*   - **Outcome type**: Dichotomous Outcome   *Necrotising enterocolitis*   - **Outcome type**: Dichotomous Outcome   *Volume in ml of blood withdrawn until hospital discharge*   - **Outcome type**: Continuous Outcome   *Volume in ml of blood transfused until hospital discharge*   - **Outcome type**: Continuous Outcome   *Number of blood transfusions until hospital discharge*   - **Outcome type**: Continuous Outcome   *Need for blood transfusions until hospital discharge*   - **Outcome type**: Dichotomous Outcome   *Concentrations of total hemoglobin (Hb)*   - **Outcome type**: Continuous Outcome   *Patent ductus arteriosus (pharmacological treatment and surgical ligation)*   - **Outcome type**: Dichotomous Outcome   *All-cause neonatal mortality (first 28 days)*   - **Outcome type**: Adverse Event   *Late sepsis until hospital discharge*   - **Outcome type**: Adverse Event   *Duration in days of hospital stay*   - **Outcome type**: Continuous Outcome |
| **Identification** | **Sponsorship source:** Not reported  **Country:** Turkey  **Setting:** Single center  **Comments:**  **Authors name:** Alan  **Institution:** Department of Pediatrics, Division of Neonatology, Ankara University School of Medicine  **Email:** alanserdar@gmail.com  **Address:** Ankara, Turkey |
| **Notes** |  |

**Risk of bias table**

| **Bias** | **Authors' judgement** | **Support for judgement** |
| --- | --- | --- |
| Random sequence generation (selection bias) | Unclear risk | Judgement Comment: Not reported |
| Allocation concealment (selection bias) | Low risk | Quote: "Before delivery, subjects were randomly assigned to one of the 2 experimental groups by using sequentially numbered sealed nontransparent envelopes."  Judgement Comment: quote "Before delivery, subjects were randomly assigned to one of the 2 experimental groups by using sequentially numbered sealed nontransparent envelopes." |
| Blinding of participants and personnel (performance bias) | High risk | Quote: "The neonatologist attending the delivery informed the obstetrician for the infant’s assignment. UCM was performed by one of the investigators (S.A.) who also took part in most of the deliveries. The intervention was unmasked for the attending neonatal and obstetric teams in the delivery room."  Judgement Comment: Quote "The neonatologist attending the delivery informed the obstetrician for the infant’s assignment. UCM was performed by one of the investigators (S.A.) who also took part in most of the deliveries. The intervention was unmasked for the attending neonatal and obstetric teams in the delivery room." |
| Blinding of outcome assessment (detection bias) | High risk | Judgement Comment: Not reported |
| Incomplete outcome data (attrition bias) | Unclear risk | Judgement Comment: Attrition and exclusions were partially explained, no clear information about the population used for counting outcomes presented as % |
| Selective reporting (reporting bias) | Unclear risk | Judgement Comment: No protocol found |
| Other bias | Low risk | Judgement Comment: None |

**Backes 2016**

| **Methods** | **Study design:** Randomized controlled trial  **Study grouping:** Parallel group |
| --- | --- |
| **Participants** | **Baseline Characteristics**  Delayed cord clamping   - *Gestational age (SD) (weeks)*: 24.4 (1.2) - *Birth weight (SD) (grams)*: 645 (193) - *Number of patients at randomization*: 18 - *Number of patients at outcome*: 18, 17   Immediate cord clamping   - *Gestational age (SD) (weeks)*: 24.6 (1.1) - *Birth weight (SD) (grams)*: 634 (160) - *Number of patients at randomization*: 22 - *Number of patients at outcome*: 22, 20   **Included criteria:** Women with singleton pregnancies between 22.5 and 27.6 weeks’ gestation who were admitted to the labor and delivery service  **Excluded criteria:** Women whose pregnancies were complicated by placental abruption, placental previa, multiple gestations, chromosomal abnormalities (including trisomy 21), known major congenital malformations, attending obstetrician refusal to participate or intent to withhold care were not eligible for enrollment.  **Pretreatment:** Comparable baseline characteristics |
| **Interventions** | **Intervention Characteristics**  Delayed cord clamping   - *Description of intervention*: The obstetrician clamped the umbilical cord 30 to 45 s following delivery of the infant. During the delay, the infant was held in a sterile towel approximately 10 to 15 inches below the mother’s introitus at vaginal delivery or below the level of the incision at cesarean section - *Time to cord clamping*: 37.4 (5.7)   Immediate cord clamping   - *Description of intervention*: The obstetrician clamped the umbilical cord immediately following delivery of the infant (<10 s) - *Time to cord clamping*: 3.8 (1.0) |
| **Outcomes** | *All-cause mortality during initial hospitalization*   - **Outcome type**: Dichotomous Outcome   *Retinopathy of prematurity*   - **Outcome type**: Dichotomous Outcome   *IVH*   - **Outcome type**: Dichotomous Outcome   *Bronchopulmonary dysplasia/chronic lung disease*   - **Outcome type**: Dichotomous Outcome   *Necrotising enterocolitis*   - **Outcome type**: Dichotomous Outcome   *Volume in ml of blood withdrawn until hospital discharge*   - **Outcome type**: Continuous Outcome   *Number of blood transfusions until hospital discharge*   - **Outcome type**: Continuous Outcome   *Concentrations of hemoglobin (Hb)*   - **Outcome type**: Continuous Outcome   *Late sepsis until hospital discharge*   - **Outcome type**: Dichotomous Outcome   *Patent ductus arteriosus (pharmacological treatment and surgical ligation)*   - **Outcome type**: Dichotomous Outcome   *All-cause neonatal mortality (first 28 days)*   - **Outcome type**: Dichotomous Outcome |
| **Identification** | **Sponsorship source:** Grant from the American Heart Association (# 10CRP3730033, CHB) and internal funding provided by Nationwide Children’s Hospital Research Institute.  **Country:** United States of America  **Setting:** Single center  **Comments:**  **Authors name:** Backes  **Institution:** The Center for Perinatal Research, Nationwide Children’s Hospital  **Email:** Carl.BackesJr@Nationwidechildrens.org  **Address:** Columbus, OH, USA |
| **Notes** |  |

**Risk of bias table**

| **Bias** | **Authors' judgement** | **Support for judgement** |
| --- | --- | --- |
| Random sequence generation (selection bias) | Low risk | Judgement Comment: A random number system was generated by a statistician not involved in the study. |
| Allocation concealment (selection bias) | Low risk | Judgement Comment: Laminated cards for randomization were maintained in sealed, opaque envelopes. |
| Blinding of participants and personnel (performance bias) | High risk | Quote: "As a result of the nature of the intervention, the study could not be blinded." |
| Blinding of outcome assessment (detection bias) | Low risk | Quote: "Prenatal and peripartum data were collected from the mothers’ medical records by a study member who was unaware of the treatment group. None of the study members present at the time of randomization or aware of group assignment participated in the daily clinical care of study patients." |
| Incomplete outcome data (attrition bias) | Low risk | Judgement Comment: Data appears to be complete. Attrition and exclusions were explained and accounted for. |
| Selective reporting (reporting bias) | Unclear risk | Judgement Comment: Protocol could not be found. |
| Other bias | Low risk | Judgement Comment: None |

**Baenziger 2007**

| **Methods** | **Study design:** Randomized controlled trial  **Study grouping:** Parallel group |
| --- | --- |
| **Participants** | **Baseline Characteristics**  Delayed cord clamping   - *Gestational age (SD) (weeks)*: 30.43 (2.30) - *Birth weight (SD) (grams)*: 1115 (343.71) - *Number of patients at randomization*: 15 - *Number of patients at outcome*: 15, 14 (depending on outcome measured)   Immediate cord clamping   - *Gestational age (SD) (weeks)*: 29.71 (2.38) - *Birth weight (SD) (grams)*: 1330 (483.95) - *Number of patients at randomization*: 24 - *Number of patients at outcome*: 24, 21 (depending on outcome measured)   **Included criteria:** Preterm neonates expected to be delivered at 24 to 32 completed weeks’ gestation  **Excluded criteria:** Multiple deliveries, children with perinatal asphyxia, major fetal malformations, and children whose parents refused consent  **Pretreatment:** Comparable baseline characteristics |
| **Interventions** | **Intervention Characteristics**  Delayed cord clamping   - *Description of procedure*: The infant was placed 15 cm below the placenta in cesarean-section deliveries and as low as possible for vaginal deliveries, and umbilical cord clamping was delayed 60 to 90 seconds. - *Time to cord clamping*: 60-90 s   Immediate cord clamping   - *Description of procedure*: Cord clamped in <20 seconds - *Time to cord clamping*: 0-20 s |
| **Outcomes** | *All-cause neonatal mortality (first 28 days)*   - **Outcome type**: Dichotomous Outcome   *Concentrations of total hemoglobin (Hb)*   - **Outcome type**: Continuous Outcome   *Mechanical ventilation (adjust to other format)*   - **Outcome type**: Dichotomous Outcome   *Late sepsis until hospital discharge*   - **Outcome type**: Dichotomous Outcome |
| **Identification** | **Sponsorship source:** Not reported  **Country:** Switzerland  **Setting:** Multi center  **Comments:**  **Authors name:** Baeznziger  **Institution:** Department of Neonatology, University Hospital Zurich  **Email:** oskar.baenziger@kispi.unizh.ch  **Address:** Zurich, Switzerland |
| **Notes** |  |

**Risk of bias table**

| **Bias** | **Authors' judgement** | **Support for judgement** |
| --- | --- | --- |
| Random sequence generation (selection bias) | Unclear risk | Judgement Comment: Sequence generation not reported. |
| Allocation concealment (selection bias) | Unclear risk | Judgement Comment: Not reported |
| Blinding of participants and personnel (performance bias) | High risk | Judgement Comment: by the nature of the study |
| Blinding of outcome assessment (detection bias) | High risk | Judgement Comment: Not reported |
| Incomplete outcome data (attrition bias) | Unclear risk | Judgement Comment: No protocol |
| Selective reporting (reporting bias) | Unclear risk | Judgement Comment: No protocol found. |
| Other bias | Unclear risk | Judgement Comment: Groups size: 15 vs 24 |

**Balasubramanian 2019**

| **Methods** | **Study design:** Randomized controlled trial  **Study grouping:** Parallel group |
| --- | --- |
| **Participants** | **Baseline Characteristics**  Blood sampling from the umbilical cord or from the placenta   - *Gestational age (SD) (weeks)*: 26.5 (1.25) - *Birth weight (SD) (grams)*: 825 (141.6) - *Number of patients at randomization*: 40 - *Number of patients at outcome*: 36   Blood sampling from the infant   - *Gestational age (SD) (weeks)*: 26.4 (1.39) - *Birth weight (SD) (grams)*: 808 (142.5) - *Number of patients at randomization*: 40 - *Number of patients at outcome*: 35   **Included criteria:** Preterm infants less than or equal to 28 weeks and/or less than or equal to 1 kg birth weight and recruited at the time of birth.  **Excluded criteria:** 1. History of clinical chorioamnionitis2. Monochorionic twins/triplets/Quadruplets3. Anomaly of the cord like true/False knots, Stricture4. Funisitis of the cord. Major congenital anomalies in the infant like Gastroschisis, Exomphalos6. Rh isoimmnisation7. HIV positive mother8. Hydrops fetalis9. Cord Blood Banking10. Placenta accreta/Marginal insertion/Battledore placenta/Velamentous insertion  **Pretreatment:** |
| **Interventions** | **Intervention Characteristics**  Blood sampling from the umbilical cord or from the placenta   - *Description of procedure*: Umbilical Cord blood sampling – Blood for initial investigations will be collected from the placental end of the umbilical cord.   Blood sampling from the infant   - *Description of procedure*: Admission blood sampling – Blood for initial investigations will be collected from the neonate on admission to the NICU. |
| **Outcomes** | *All-cause mortality during initial hospitalization*   - **Outcome type**: Dichotomous Outcome   *Retinopathy of prematurity*   - **Outcome type**: Dichotomous Outcome   *IVH*   - **Outcome type**: Dichotomous Outcome   *Bronchopulmonary dysplasia/chronic lung disease*   - **Outcome type**: Dichotomous Outcome   *Necrotising enterocolitis*   - **Outcome type**: Dichotomous Outcome   *Volume in ml of blood transfused until hospital discharge*   - **Outcome type**: Continuous Outcome - **Notes**: CUSTOM (MEDIAN, IQR) NEEDS TO BE MANUALLY EXPORTED TO REVMAN.   *Need for blood transfusions until hospital discharge*   - **Outcome type**: Dichotomous Outcome   *Concentrations of total hemoglobin (Hb)*   - **Outcome type**: Continuous Outcome   *Late sepsis until hospital discharge*   - **Outcome type**: Dichotomous Outcome   *Patent ductus arteriosus (pharmacological treatment and surgical ligation)*   - **Outcome type**: Dichotomous Outcome   *Duration in days of respiratory support*   - **Outcome type**: Continuous Outcome - **Notes**: CUSTOM (MEDIAN, IQR) NEEDS TO BE MANUALLY EXPORTED TO REVMAN.   *Duration in days of hospital stay*   - **Outcome type**: Continuous Outcome - **Notes**: CUSTOM (MEDIAN, IQR) - NEEDS TO BE MANUALLY EXPORTED TO REVMAN |
| **Identification** | **Sponsorship source:** Indian National Neonatal Collaborative  **Country:** India  **Setting:** NICU  **Comments:**  **Authors name:** H. Balasubramanian  **Institution:**  **Email:** drhari@suryahospitals.com  **Address:** |
| **Notes** |  |

**Risk of bias table**

| **Bias** | **Authors' judgement** | **Support for judgement** |
| --- | --- | --- |
| Random sequence generation (selection bias) | Low risk | Judgement Comment: Quote: "Random sequences with varying block sizes were generated by a statistician who was not part of the study". Quote: "Random sequences with varying block sizes were generated by a statistician who was not part of the study". |
| Allocation concealment (selection bias) | Low risk | Judgement Comment: Quote: "Allocation concealment was performed using sequentially numbered opaque sealed envelopes." |
| Blinding of participants and personnel (performance bias) | High risk | Judgement Comment: The clinicians performing the interventions were not blinded. Quote: "Because of the nature of the study, it was not feasible to blind the clinicians performing the sampling technique." Whether the clinicians caring for the infants during the subsequent weeks – and potentially affecting the outcomes – were blinded to the intervention is not mentioned. |
| Blinding of outcome assessment (detection bias) | Low risk | Judgement Comment: Quote: "However, the outcome assessors (laboratory technicians, primary investigators) were blinded to the intervention." |
| Incomplete outcome data (attrition bias) | Low risk | Judgement Comment: 80 neonates were randomized in two groups (CBS, ABS) with equal sample size (n=40). All 80 neonates were included in the survival analysis. 8 neonates were discharged; 4 were transferred out and 4 died. 36 and 35 neonates in the CBS and ABS group, respectively, were analyzed for secondary outcomes (89% of the neonates). After randomization to CBS, cord blood samples of 3 neonates were clotted. 1 neonate enrolled in the CBS group received the intervention of the other group when admission blood samples were accidentally drawn. |
| Selective reporting (reporting bias) | Unclear risk | Judgement Comment: The primary outcome of "Days until first blood transfusion" matches the published protocol. Out of 30 reported secondary outcomes, only 13 is specified in the protocol – e.g. "mortality" is not a pre-specified outcome in the protocol. Some secondary outcomes are reported in the protocol but not reported in the article. "Time to 30% reduction in hemoglobin" is not reported but other hemoglobin measures are presented. "Severe hyperbilirubinemia" is stated in the protocol but not reported in the article, and vice versa for "Duration of respiratory support". |
| Other bias | Low risk | Judgement Comment: The authors chose to only randomize neonates after successful cord sampling, basically declaring it as an inclusion criterion. This does not create any bias in selection of the population whereas might be considered in the assessment of indirectness in GRADE. Quote: "If cord sampling was successful and the weight at birth was less than 1000 g, neonates were considered eligible for study enrollment." They did comment on it in their discussion, saying it was done to not compromise the treatment fidelity. Quote: "However, we chose to randomize infants after cord blood collection because failed attempts at cord sampling were perceived to compromise the treatment fidelity between the 2 groups, especially with small sample sizes." |

**Chu 2019**

| **Methods** | **Study design:** Randomized controlled trial  **Study grouping:** Parallel group |
| --- | --- |
| **Participants** | **Baseline Characteristics**  Delayed cord clamping   - *Gestational age (SD) (weeks)*: 26.4 (0.9) - *Birth weight (SD) (grams)*: 1144.7 (347.4) - *Number of patients at randomization*: 19 - *Number of patients at outcome*: 19   Immediate cord clamping   - *Gestational age (SD) (weeks)*: 29.4 (2.0) - *Birth weight (SD) (grams)*: 1311.0 (457.6) - *Number of patients at randomization*: 19 - *Number of patients at outcome*: 19   **Included criteria:** Singleton pregnancy that were in labor or required birth between 24 to 32 weeks of gestational age, as determined by the last menstrual period and/or early dating ultrasound  **Excluded criteria:** Major life threatening fetal anomalies, multiple gestations, intrauterine fetal demise, or plan for stem cell collection and cord blood banking  **Pretreatment:** Comparable baseline characteristics |
| **Interventions** | **Intervention Characteristics**  Delayed cord clamping   - *Description of procedure*: The obstetrician held the infant in a warm towel 10–15 cm below the introitus (vaginal births) or at the incision level (cesarean section) ensuring no tension was placed on the umbilical cord and cut between 30-45 s - *Time to cord clamping*: 39.7 (36.2)   Immediate cord clamping   - *Description of procedure*: The umbilical cord was clamped and cut within 10 s of birth - *Time to cord clamping*: 5.4 (5.0) |
| **Outcomes** | *All-cause mortality during initial hospitalization*   - **Outcome type**: Dichotomous Outcome   *IVH*   - **Outcome type**: Dichotomous Outcome   *Need for blood transfusions until hospital discharge*   - **Outcome type**: Dichotomous Outcome   *Concentrations of total hemoglobin (Hb)*   - **Outcome type**: Continuous Outcome   *Late sepsis until hospital discharge*   - **Outcome type**: Dichotomous Outcome |
| **Identification** | **Sponsorship source:** Josie Chundamala, Scientific Grant Editor funded by the Department of Obstetrics and Gynecology at Mount Sinai Hospital assisted in editing and preparing this manuscript for submission  **Country:** Canada  **Setting:** Single center  **Comments:**  **Authors name:** Chu  **Institution:** Department of Obstetrics and Gynecology, University of Toronto  **Email:** Kellie.Murphy@sinaihealth.ca  **Address:** Toronto, Canada |
| **Notes** | Late onset sepsis is defined as >48hrs  Our protocol: LOS >72 hrs, contacted authors but no response |

**Risk of bias table**

| **Bias** | **Authors' judgement** | **Support for judgement** |
| --- | --- | --- |
| Random sequence generation (selection bias) | Unclear risk | Judgement Comment: Randomly generated card; generation process not described |
| Allocation concealment (selection bias) | Low risk | Quote: "sealed opaque envelopes" |
| Blinding of participants and personnel (performance bias) | High risk | Quote: "Given the nature of this trial, it was not possible to blind the participant, obstetrician, or neonatal resuscitation team as all needed to be present at the time of birth."  Judgement Comment: No blinding due to the nature of the study |
| Blinding of outcome assessment (detection bias) | Low risk | Quote: "recruitment into the trial was not recorded on the infant’s chart and, therefore, the team caring for the neonate in the Neonatal Intensive Care Unit (NICU) continued to manage and treat the neonate at the discretion of the attending NICU neonatologist, who was not present at the time of birth." |
| Incomplete outcome data (attrition bias) | Low risk | Judgement Comment: Data appears to be complete. Attrition and exclusions were explained and accounted for. |
| Selective reporting (reporting bias) | Unclear risk | Judgement Comment: no report of grade IV in results section |
| Other bias | Unclear risk | Quote: "Two cases in the DCC group did not fall within their designated cord clamping window time. One was the result of an infant that appeared unstable and a delay in resuscitation was thought to compromise neonatal well-being due to a tight nuchal cord at the time of birth that necessitated immediate cord clamping. The second was due to a protocol violation resulting from a communication failure that caused a prolonged delay of cord clamping of 180"  Judgement Comment: No units given in the tables. We had to assume the units for Hb. |

**Dipak 2017**

| **Methods** | **Study design:** Randomized controlled trial  **Study grouping:** Parallel group |
| --- | --- |
| **Participants** | **Baseline Characteristics**  Delayed cord clamping   - *Gestational age (SD) (weeks)*: 30.1(1.2) - *Birth weight (SD) (grams)*: 1316.1 (162.9) - *Number of patients at randomization*: 26 - *Number of patients at outcome*: 26   Immediate cord clamping   - *Gestational age (SD) (weeks)*: 29.9 (1.4) - *Birth weight (SD) (grams)*: 1283.7 (176.4) - *Number of patients at randomization*: 27 - *Number of patients at outcome*: 27   **Included criteria:** Mothers with 27-31.86 weeks’ gestation with preterm onset of labor  **Excluded criteria:** Mothers with multiple gestation, Rh-ve status, placenta previa or abruption-placenta, and those having fetus with major congenital anomalies, hydrops, fetal growth restriction with abnormal Doppler waveforms, or evidence of foetal distress  **Pretreatment:** Comparable baseline characteristics |
| **Interventions** | **Intervention Characteristics**  Delayed cord clamping   - *Description of procedure*: Neonates were held in a pre-warmed towel approximately 10-15 inches below the introitus at vaginal delivery/below the level of placental incision in caesarean delivery, and cord was clamped at 60 seconds - *Time to cord clamping*: 60 s   Immediate cord clamping   - *Description of procedure*: The obstetrician clamped the umbilical cord at 10 seconds and baby was held supine at level of introitus/placental incision - *Time to cord clamping*: 10 s |
| **Outcomes** | *Retinopathy of prematurity*   - **Outcome type**: Dichotomous Outcome   *Need for blood transfusions until hospital discharge*   - **Outcome type**: Dichotomous Outcome   *Concentrations of total hemoglobin (Hb)*   - **Outcome type**: Continuous Outcome   *Late sepsis until hospital discharge*   - **Outcome type**: Dichotomous Outcome   *Patent ductus arteriosus (pharmacological treatment and surgical ligation)*   - **Outcome type**: Dichotomous Outcome   *IVH*   - **Outcome type**: Dichotomous Outcome |
| **Identification** | **Sponsorship source:** None  **Country:** India  **Setting:** Single center  **Comments:**  **Authors name:** Dipak  **Institution:** Department of Neonatology, Seth GS Medical College and KEM Hospital  **Email:** drndipak@gmail.com  **Address:** Parel, Mumbai, India |
| **Notes** |  |

**Risk of bias table**

| **Bias** | **Authors' judgement** | **Support for judgement** |
| --- | --- | --- |
| Random sequence generation (selection bias) | Low risk | Quote: "Allocation of groups was done by random number sequence with variable block size of 3 or 6 using a ‘Random Allocation Software’ program." |
| Allocation concealment (selection bias) | Low risk | Quote: "The sequence was concealed in serially numbered, opaque, sealed and identical envelopes." |
| Blinding of participants and personnel (performance bias) | High risk | Judgement Comment: due to the nature of the study |
| Blinding of outcome assessment (detection bias) | High risk | Judgement Comment: Not reported |
| Incomplete outcome data (attrition bias) | Low risk | Judgement Comment: Data appears to be complete. Attrition and exclusions were explained and accounted for |
| Selective reporting (reporting bias) | High risk | Judgement Comment: no protocol published, PDA reported in the outcomes but not listed in the methods section, unclear definition of "brain injury", no report on IVH specifically, even if listed in the methods |
| Other bias | Low risk | Judgement Comment: None |

**Dong 2016**

| **Methods** | **Study design:** Randomized controlled trial  **Study grouping:** Parallel group |
| --- | --- |
| **Participants** | **Baseline Characteristics**  Delayed cord clamping   - *Gestational age (SD) (weeks)*: 29.5 (1.7) - *Birth weight (SD) (grams)*: 1291 (276) - *Number of patients at randomization*: 46 - *Number of patients at outcome*: 46   Immediate cord clamping   - *Gestational age (SD) (weeks)*: 29.1 (1.6) - *Birth weight (SD) (grams)*: 1241 (301) - *Number of patients at randomization*: 44 - *Number of patients at outcome*: 44   **Included criteria:** Gestational age <32 weeks; natural delivery, singleton, no severe developmental deformities  **Excluded criteria:** Premature infants who need immediate recovery, placenta previa and placental abruption  **Pretreatment:** Comparable baseline characteristics |
| **Interventions** | **Intervention Characteristics**  Delayed clamping or cord milking   - *Description of procedure*: The infant was positioned 10-20 cm below the placenta, the umbilical cord was cut at 45 seconds - *Time to cord clamping*: 45 s   Immediate cord clamping   - *Description of procedure*: Umbilical cord ligation within10 s - *Time to cord clamping*: 10 s |
| **Outcomes** | *Retinopathy of prematurity*   - **Outcome type**: Dichotomous Outcome   *IVH*   - **Outcome type**: Dichotomous Outcome   *Bronchopulmonary dysplasia/chronic lung disease*   - **Outcome type**: Dichotomous Outcome   *Necrotising enterocolitis*   - **Outcome type**: Dichotomous Outcome   *Need for blood transfusions until hospital discharge*   - **Outcome type**: Dichotomous Outcome   *Concentrations of total hemoglobin (Hb)*   - **Outcome type**: Continuous Outcome   *Late sepsis until hospital discharge*   - **Outcome type**: Dichotomous Outcome   *Volume in ml/kg of blood transfused until hospital discharge*   - **Outcome type**: Continuous Outcome |
| **Identification** | **Sponsorship source:** Not reported  **Country:** China  **Setting:** Single center  **Comments:**  **Authors name:** Dong  **Institution:** Department of Pediatrics, Nanjing Maternal and Child Health Hospital Affiliated to Nanjing Medical University  **Email:** shupinghan@njmu.edu.cn  **Address:** Nanjing, China |
| **Notes** |  |

**Risk of bias table**

| **Bias** | **Authors' judgement** | **Support for judgement** |
| --- | --- | --- |
| Random sequence generation (selection bias) | Unclear risk | Judgement Comment: Not reported |
| Allocation concealment (selection bias) | Unclear risk | Judgement Comment: Not reported (data extracted from translated paper) |
| Blinding of participants and personnel (performance bias) | High risk | Judgement Comment: Not reported |
| Blinding of outcome assessment (detection bias) | High risk | Judgement Comment: Not reported |
| Incomplete outcome data (attrition bias) | Low risk | Judgement Comment: All infants were accounted for. |
| Selective reporting (reporting bias) | Unclear risk | Judgement Comment: Data extracted from translated paper. No protocol could be found. Outcomes listed in the methods section are also reported as the results |
| Other bias | Low risk | Judgement Comment: None |

**Duley 2018**

| **Methods** | **Study design:** Randomized controlled trial  **Study grouping:** Parallel group |
| --- | --- |
| **Participants** | **Baseline Characteristics**  Delayed cord clamping   - *Gestational age (median) (weeks)*: 28.9 - *Birth weight, median (IQR) (grams)*: 1108 (880–1360) - *Number of patients at randomization*: 137 - *Number of patients at outcome*: 135   Immediate cord clamping   - *Gestational age (median) (weeks)*: 29.2 - *Birth weight, median (IQR) (grams)*: 1180 (900–1418) - *Number of patients at randomization*: 139 - *Number of patients at outcome*: 134   **Included criteria:** Live birth before 32 weeks gestation  **Excluded criteria:** Monochorionic twins; triplets or higher-order multiple pregnancy and known major congenital malformation  **Pretreatment:** Comparable baseline characteristics |
| **Interventions** | **Intervention Characteristics**  Delayed cord clamping   - *Description of procedure*: Umbilical cord clamping after at least 2min and, if needed, immediate neonatal stabilisation and resuscitation with cord intact. Until cord clamping, the baby was kept at the level of placenta (introitus or mothers’ abdomen, or if a caesarean birth the anterior thigh) - *Time to cord clamping (IQR)* : 120 s (36-134)   Immediate cord clamping   - *Description of procedure*: Clamping within 20s and, if needed, immediate neonatal stabilisation and resuscitation after clamping - *Time to cord clamping (IQR)* : 11 s (10-20) |
| **Outcomes** | *All-cause neonatal mortality (first 28 days)*   - **Outcome type**: Dichotomous Outcome   *All-cause mortality during initial hospitalization*   - **Outcome type**: Dichotomous Outcome   *Major neurodevelopmental disability*   - **Outcome type**: Dichotomous Outcome   *Retinopathy of prematurity*   - **Outcome type**: Dichotomous Outcome   *IVH*   - **Outcome type**: Dichotomous Outcome   *Bronchopulmonary dysplasia/chronic lung disease*   - **Outcome type**: Dichotomous Outcome   *Necrotising enterocolitis*   - **Outcome type**: Dichotomous Outcome   *Volume in ml of blood withdrawn until hospital discharge*   - **Outcome type**: Continuous Outcome   *Volume in ml of blood transfused until hospital discharge*   - **Outcome type**: Continuous Outcome   *Need for blood transfusions until hospital discharge*   - **Outcome type**: Dichotomous Outcome   *Concentrations of total hemoglobin (Hb)*   - **Outcome type**: Continuous Outcome   *Late sepsis until hospital discharge*   - **Outcome type**: Dichotomous Outcome   *Patent ductus arteriosus (pharmacological treatment and surgical ligation)*   - **Outcome type**: Dichotomous Outcome   *Duration in days of respiratory support*   - **Outcome type**: Continuous Outcome   *Duration in days of supplemental oxygen requirement*   - **Outcome type**: Continuous Outcome   *Duration in days of hospital stay*   - **Outcome type**: Continuous Outcome   *Composite outcome: Major Developmental Disability*   - **Outcome type**: Dichotomous Outcome   *Impaired Motor skills*   - **Outcome type**: Dichotomous Outcome |
| **Identification** | **Sponsorship source:** This trial is independent research funded by the National Institute for Health Research (NIHR) under its Programme Grants for Applied Research funding scheme (RPPG-0609-10107).  **Country:** United Kingdom  **Setting:** Multi center  **Comments:**  **Authors name:** Duley  **Institution:** Nottingham Clinical Trials Unit, Queen’s Medical Centre, University of Nottingham  **Email:** lelia.duley@nottingham.ac.uk  **Address:** Nottingham, UK |
| **Notes** | 8 references all belonging to 1 RCT - Duley 2018 "Randomised trial of cord clamping and initial stabilisation at very preterm birth." Trials 2014 is the protocol, Trials 2015 is an update to the protocol. Armstrong-Buisseret & Mitchell 2019 is outcomes at two years. The three references from 2013, 2014 are poster/meeting abstracts posted during the study period. |

**Risk of bias table**

| **Bias** | **Authors' judgement** | **Support for judgement** |
| --- | --- | --- |
| Random sequence generation (selection bias) | Low risk | Quote: "Sequence generation (1:1) was by computer, stratified by center with balanced blocks of randomly varying size, created by NCTU." |
| Allocation concealment (selection bias) | Low risk | Quote: "Randomisation was by attending clinicians, who took the next sealed consecutively numbered opaque envelope from a ring binder folder." |
| Blinding of participants and personnel (performance bias) | High risk | Judgement Comment: due to the nature of the study |
| Blinding of outcome assessment (detection bias) | Unclear risk | Quote: "single assessor reviewed the cranial ultrasound scan reports for intraventricular hemorrhage, blind to the allocated group. Then eight trained clinicians (neonatologists or radiologists) independently adjudicated each scan, blind to allocation. 27 If the adjudication disagreed with the scan report review, a second independent adjudicator assessed the scan images. Remaining discrepancies were resolved by discussion."  Judgement Comment: IVH assessment was blinded to the allocated group; unclear for other outcomes |
| Incomplete outcome data (attrition bias) | Low risk | Judgement Comment: Data appears to be complete. Attrition and exclusions were explained and accounted for.)  Lost for follow up 20% in one group. |
| Selective reporting (reporting bias) | Low risk | Judgement Comment: Protocol found, no selective reporting. |
| Other bias | Low risk | Judgement Comment: None |

**Elimian 2014**

| **Methods** | **Study design:** Randomized controlled trial  **Study grouping:** Parallel group |
| --- | --- |
| **Participants** | **Baseline Characteristics**  Delayed cord clamping   - *Gestational age (SD) (weeks)*: 30.9 (3.1) - *Birth weight (SD) (grams)*: 1661 (598) - *Number of patients at randomization*: 99 - *Number of patients at outcome*: 99   Immediate cord clamping   - *Gestational age (SD) (weeks)*: 30.7 (2.8) - *Birth weight (SD) (grams)*: 1542 (555) - *Number of patients at randomization*: 101 - *Number of patients at outcome*: 101   **Included criteria:** Singleton pregnancies, between 24 weeks 0 days and 34 weeks 0 days of gestation who were deemed to be at risk of being delivered prematurely  **Excluded criteria:** Fetuses with known major fetal structural or chromosomal abnormalities, multiple gestations, diabetes, intrauterine growth restriction, or non-reassuring fetal heart tracings  **Pretreatment:** Comparable baseline characteristics |
| **Interventions** | **Intervention Characteristics**  Delayed cord clamping   - *Description of procedure*: Clamping of the cord after 30 seconds after birth. Three to four passes of milking of the umbilical cord toward the neonate was allowed in all neonates in the delayed cord clamping group - *Time to cord clamping*: 32 (31–35) s   Immediate cord clamping   - *Description of procedure*: Clamping the umbilical cord within 5 seconds of delivery - *Time to cord clamping*: 0-5 s |
| **Outcomes** | *All-cause neonatal mortality (first 28 days)*   - **Outcome type**: Dichotomous Outcome   *IVH*   - **Outcome type**: Dichotomous Outcome   *White matter at term-equivalent MRI abnormalities at term equivalent age*   - **Outcome type**: Dichotomous Outcome   *Bronchopulmonary dysplasia/chronic lung disease*   - **Outcome type**: Dichotomous Outcome   *Necrotising enterocolitis*   - **Outcome type**: Dichotomous Outcome   *Need for blood transfusions until hospital discharge*   - **Outcome type**: Dichotomous Outcome   *Concentrations of total hemoglobin (Hb)*   - **Outcome type**: Continuous Outcome   *Patent ductus arteriosus (pharmacological treatment and surgical ligation)*   - **Outcome type**: Dichotomous Outcome   *Duration in days of respiratory support*   - **Outcome type**: Continuous Outcome   *Duration in days of supplemental oxygen requirement*   - **Outcome type**: Continuous Outcome |
| **Identification** | **Sponsorship source:** Not reported  **Country:** United States of America  **Setting:** Single center  **Comments:** We included IVH outcomes for infants born below or at 26 weeks of gestation  **Authors name:** Elimian  **Institution:** Departments of Obstetrics and Gynecology and Pediatrics, University of Oklahoma Health Sciences Center, Williams Pavilion  **Email:** andrew_elimian@nymc.edu  **Address:** Oklahoma City, Oklahoma, USA |
| **Notes** |  |

**Risk of bias table**

| **Bias** | **Authors' judgement** | **Support for judgement** |
| --- | --- | --- |
| Random sequence generation (selection bias) | Low risk | Quote: "Allocation sequence was generated by simple randomization using a computer." |
| Allocation concealment (selection bias) | Low risk | Quote: "The allocation sequence was concealed by using sequentially numbered, opaque, sealed envelopes kept in a central location on labor and delivery." |
| Blinding of participants and personnel (performance bias) | High risk | Judgement Comment: Due to the nature of the study |
| Blinding of outcome assessment (detection bias) | High risk | Judgement Comment: No clear information |
| Incomplete outcome data (attrition bias) | Low risk | Judgement Comment: Data appears to be complete. Attrition and exclusions were explained and accounted for. |
| Selective reporting (reporting bias) | Low risk | Judgement Comment: Protocol found. All outcomes reported. |
| Other bias | Unclear risk | Judgement Comment: Study population with uneven distribution of infants for GA |

**El Naggar 2019**

| **Methods** | **Study design:** Randomized controlled trial  **Study grouping:** Parallel group |
| --- | --- |
| **Participants** | **Baseline Characteristics**  Cord milking   - *Gestational age (SD) (weeks)*: 27.6 (1.8) - *Birth weight (SD) (grams)*: 1061 (383) - *Number of patients at randomization*: 37 - *Number of patients at outcome*: 37   Immediate cord clamping   - *Gestational age (SD) (weeks)*: 27.2 (2) - *Birth weight (SD) (grams)*: 1019 (282) - *Number of patients at randomization*: 36 - *Number of patients at outcome*: 36   **Included criteria:** Preterm infants born between 24 and 30+6 weeks’ gestation  **Excluded criteria:** Monochorionic twins, major congenital anomalies, placental abruption, fetal anaemia and intention to withhold resuscitation  **Pretreatment:** Comparable baseline characteristics apart from more women with gestational diabetes in the ICC group |
| **Interventions** | **Intervention Characteristics**  Cord milking   - *Description of procedure*: Infants placed at or below the level of the placenta and about 20cm of the cord (or if less, the available length of cord) was milked towards the umbilicus three times, holding the cord at the end of each strip to allow for refilling in between strips, before clamping. Speed of milking was approximately 10 cm/s - *Time to cord clamping*: Not reported   Immediate cord clamping   - *Description of procedure*: Infants had their umbilical cords clamped within 10s of birth - *Time to cord clamping*: 0-10 s |
| **Outcomes** | *All-cause mortality during initial hospitalization*   - **Outcome type**: Dichotomous Outcome   *Retinopathy of prematurity*   - **Outcome type**: Dichotomous Outcome   *IVH*   - **Outcome type**: Dichotomous Outcome   *Bronchopulmonary dysplasia/chronic lung disease*   - **Outcome type**: Dichotomous Outcome   *Necrotising enterocolitis*   - **Outcome type**: Dichotomous Outcome   *Number of blood transfusions until hospital discharge*   - **Outcome type**: Continuous Outcome   *Concentrations of total hemoglobin (Hb)*   - **Outcome type**: Continuous Outcome   *Late sepsis until hospital discharge*   - **Outcome type**: Dichotomous Outcome   *Patent ductus arteriosus (pharmacological treatment and surgical ligation)*   - **Outcome type**: Dichotomous Outcome   *Duration in days of hospital stay*   - **Outcome type**: Continuous Outcome   *Composite outcome: Major Developmental Disability*   - **Outcome type**: Dichotomous Outcome   *Impaired Motor skills*   - **Outcome type**: Dichotomous Outcome |
| **Identification** | **Sponsorship source:** This project was supported by grants from both Nova Scotia Health Research Foundation (PSO-EST-2013-9023 EGMS 1813) and IWK Research Foundation (1008052). No honorarium or other form of payment was given to anyone to produce this manuscript.  **Country:** Canada  **Setting:** Single center  **Comments:** Additional outcomes taken from the El-Naggar 2019 abstract (The effect of umbilical cord milking on neurodevelopmental outcomes of preterm infants at 36 months of age: A randomized controlled trial) with a subpopulation of the original patient population. For this reason, some of the outcomes reported have a smaller patient number.  **Authors name:** El-Naggar  **Institution:** Division of Neonatal-Perinatal Medicine, Department of Pediatrics, Dalhousie University  **Email:** walid.el-naggar@iwk.nshealth.ca  **Address:** Halifax, Nova Scotia, Canada |
| **Notes** |  |

**Risk of bias table**

| **Bias** | **Authors' judgement** | **Support for judgement** |
| --- | --- | --- |
| Random sequence generation (selection bias) | Low risk | Quote: "variable block sizes randomisation table." |
| Allocation concealment (selection bias) | Low risk | Quote: "Concealed opaque envelopes were prepared ahead of time and were opened just before the time of delivery." |
| Blinding of participants and personnel (performance bias) | High risk | Judgement Comment: due to the nature of the study |
| Blinding of outcome assessment (detection bias) | Unclear risk | Quote: "Despite our efforts to keep the healthcare providers blinded to the study intervention by not documenting the intervention in the charts, we cannot be absolutely sure that full blinding was achieved."  Quote: "The type of intervention was not documented in the mother’s or infant’s chart and was not communicated to the neonatal team at birth." |
| Incomplete outcome data (attrition bias) | Low risk | Judgement Comment: Data appears to be complete. Attrition and exclusions were explained and accounted for. |
| Selective reporting (reporting bias) | Low risk | Judgement Comment: Protocol found and all outcomes reported. |
| Other bias | Low risk | Judgement Comment: None |

**Finn 2019**

| **Methods** | **Study design:** Randomized controlled trial  **Study grouping:** Parallel group |
| --- | --- |
| **Participants** | **Baseline Characteristics**  Delayed cord clamping   - *Gestational age (median) (IQR)*: 28 (26.4-29.6) - *Birth weight (median) (IQR)*: 925 (630-1490) - *Number of patients at randomization*: 14 - *Number of patients at outcome*: 14   Immediate cord clamping   - *Gestational age (median) (IQR)*: 28.5 (25.7-30.5) - *Birth weight (median) (IQR)*: 1080 (755-1613) - *Number of patients at randomization*: 12 - *Number of patients at outcome*: 12   Cord milking   - *Gestational age (median) (IQR)*: 28.4 (25.7-29.6) - *Birth weight (median) (IQR)*: 930 (700-1545) - *Number of patients at randomization*: 19 - *Number of patients at outcome*: 18   **Included criteria:** Infants born at <32 weeks of gestation  **Excluded criteria:** Major congenital anomaly, bleeding from placenta previa, placental abruption or accreta, twin-to-twin transfusion syndrome, hydrops, and cord prolapse  **Pretreatment:** Comparable baseline characteristics |
| **Interventions** | **Intervention Characteristics**  Delayed cord clamping   - *Description of procedure*: The infant was placed on a mobile resuscitation trolley (Lifestart, Inspiration Healthcare, Leicester, UK) with the cord intact, at or below the level of the placenta. Routine neonatal care was provided, including positive end-expiratory pressure and the provision of positive pressure ventilation if required, and the cord was clamped at 60 seconds after delivery - *Time to cord clamping*: 60 s   Immediate cord clamping   - *Description of procedure*: Immediate cord clamping was defined as clamping the umbilicus within 20 seconds of delivery - *Time to cord clamping*: 0-20 s   Cord milking   - *Description of procedure*: The obstetrician held the infant at or below the level of the placenta and an assistant stripped the cord, 20 cm over 2 seconds, 3 times in the direction of the infant - *Time to cord clamping*: Not reported |
| **Outcomes** | *Retinopathy of prematurity*   - **Outcome type**: Dichotomous Outcome   *IVH*   - **Outcome type**: Dichotomous Outcome   *Bronchopulmonary dysplasia/chronic lung disease*   - **Outcome type**: Dichotomous Outcome   *Necrotising enterocolitis*   - **Outcome type**: Dichotomous Outcome   *Number of blood transfusions until hospital discharge*   - **Outcome type**: Continuous Outcome   *Concentrations of total hemoglobin (Hb)*   - **Outcome type**: Continuous Outcome   *Late sepsis until hospital discharge*   - **Outcome type**: Dichotomous Outcome   *Duration in days of respiratory support*   - **Outcome type**: Continuous Outcome |
| **Identification** | **Sponsorship source:** Supported by 2 awards from Science Foundation Ireland, a Research Centre Award (INFANT-12/RC/2272), and an Investigator Award (15/SIRG/3580 [to J.T.]).  **Country:** Ireland  **Setting:** Single center  **Comments:**  **Authors name:** Finn  **Institution:** Department of Pediatrics and Child Health and the Department of Obstetrics, Cork University Maternity Hospital  **Email:** daragh.finn@hse.ie  **Address:** Cork, Ireland |
| **Notes** |  |

**Risk of bias table**

| **Bias** | **Authors' judgement** | **Support for judgement** |
| --- | --- | --- |
| Random sequence generation (selection bias) | Low risk | Quote: "Randomization of groups was performed using a computer program" |
| Allocation concealment (selection bias) | Low risk | Quote: "allocation concealment was achieved by using opaque, sequentially numbered, sealed envelopes." |
| Blinding of participants and personnel (performance bias) | High risk | Judgement Comment: Due to the nature of the study |
| Blinding of outcome assessment (detection bias) | High risk | Judgement Comment: No information about clinicians/ researchers assessing the outcomes (except for cranial ultrasound) |
| Incomplete outcome data (attrition bias) | Low risk | Judgement Comment: Data appears to be complete. Attrition and exclusions were explained and accounted for. |
| Selective reporting (reporting bias) | High risk | Judgement Comment: the protocol was published retrospectively, no all the outcomes listed in the protocol are reported in the publication |
| Other bias | Low risk | Judgement Comment: None |

**Galderisi 2017**

| **Methods** | **Study design:** Randomized controlled trial  **Study grouping:** Parallel group |
| --- | --- |
| **Participants** | **Baseline Characteristics**  Normal blood sampling   - *Gestational age (Median (IQR)) (weeks)*: 30 (28–31) - *Birth weight (Median (IQR)) (grams)*: 1300 (1100–1760) - *Number of patients at randomization*: 25 - *Number of patients at outcome*: 24   Devices to monitor glucose levels, subcutaneous   - *Gestational age (Median (IQR)) (weeks)*: 30 (29-31) - *Birth weight (Median (IQR)) (grams)*: 1170 (1100–1595) - *Number of patients at randomization*: 25 - *Number of patients at outcome*: 20   **Included criteria:** < 32 weeks’ gestation and/or birthweight <1500 g.  **Excluded criteria:** Birthweight <500g; Malformative syndrome; Lack of parental consent; Chromosomal abnormalities  **Pretreatment:** |
| **Interventions** | **Intervention Characteristics**  Normal blood sampling   - *Description of procedure*: Hypo/hyper alarms are off. CGM data will be blinded. Glucose intake will be adequate according to 2-3 capillary glycemic tests per day.   Devices to monitor glucose levels, subcutaneous   - *Description of procedure*: CGM data will be "unblinded", with Hypo/hyperglycemia alarms on. Data will be recorded from CGM every three hours and intervention to adequate glucose intake will be performed to keep glycemia in normal range (72-144mg/dl) if necessary. |
| **Outcomes** | *All-cause neonatal mortality (first 28 days)*   - **Outcome type**: Dichotomous Outcome   *All-cause mortality during initial hospitalization*   - **Outcome type**: Dichotomous Outcome   *Late sepsis until hospital discharge*   - **Outcome type**: Dichotomous Outcome   *Duration in days of hospital stay*   - **Outcome type**: Continuous Outcome - **Notes**: CUSTOM (MEDIAN, IQR) NEEDS TO BE MANUALLY EXPORTED TO REVMAN.   *Pain score during device insertion*   - **Outcome type**: Continuous Outcome - **Notes**: CUSTOM (MEDIAN, RANGES) NEEDS TO BE MANUALLY EXPORTED TO REVMAN.   *IVH*   - **Outcome type**: Dichotomous Outcome - **Direction**: Lower is better |
| **Identification** | **Sponsorship source:** University Hospital of Padua  **Country:** Italy  **Setting:** Single center  **Comments:**  **Authors name:** A. Galderisi  **Institution:**  **Email:**  **Address:** |
| **Notes** |  |

**Risk of bias table**

| **Bias** | **Authors' judgement** | **Support for judgement** |
| --- | --- | --- |
| Random sequence generation (selection bias) | Low risk | Judgement Comment: Electronically generated block randomization. Was performed by an officer not involved in the study. |
| Allocation concealment (selection bias) | Low risk | Judgement Comment: Sequentially numbered, opaque, sealed envelopes generated electronically. Performed by an officer not involved in the study. Sequentially numbered, opaque, sealed envelopes generated electronically. Performed by an officer not involved in the study. |
| Blinding of participants and personnel (performance bias) | High risk | Judgement Comment: Treating personnel could not be blinded due to the nature of the study where the real-time CGM will give off a loud alarm, thus revealing the group assignment. |
| Blinding of outcome assessment (detection bias) | Unclear risk | Judgement Comment: In the method, the authors state: "Data were electronically anonymized by using an individual alphanumeric code and analyzed by investigators not involved in patient enrollment or data collection." However, they also state in the discussion that, quote: "An additional limit of our study includes the lack of investigator blinding." |
| Incomplete outcome data (attrition bias) | Low risk | Judgement Comment: Out of the 50 infants randomized to the two groups, 6 discontinued the allocated intervention (4 due to hospital transfers, all in the intervention group; 2 due to sensor detachment, 1 in each group). All 50 infants were included in Intention To Treat-analysis. |
| Selective reporting (reporting bias) | Unclear risk | Judgement Comment: Outcomes reported compared against protocol. Secondary outcomes not reported in study, but present in protocol: - Acute event associated to glycemic variability and/or hypoglycemia - Glycemic modeling - Pain associated to insertion of CGM respect to Pain associated to heel prick (reported for 7 infants in separate Conference Abstract from 2016). |
| Other bias | Unclear risk | Judgement Comment: Differences in baseline characteristics could introduce a bias and having an effect on outcomes. Authors state that characteristics arise similar between groups, but no p-values of difference between groups are given. Example: 4 vs 0 for small gestation age, 6 vs 11 for twins (large amount of twins??), 4 vs 1 for maternal diabetes. |

**Gokmen 2011**

| **Methods** | **Study design:** Randomized controlled trial  **Study grouping:** Parallel group |
| --- | --- |
| **Participants** | **Baseline Characteristics**  Delayed cord clamping   - *Gestational age (SD) (weeks)*: 29.3 (1.2) - *Birth weight (SD) (grams)*: 1360 (413) - *Number of patients at randomization*: 21 - *Number of patients at outcome*: 21   Immediate cord clamping   - *Gestational age (SD) (weeks)*: 29.4 (1.5) - *Birth weight (SD) (grams)*: 1323 (358) - *Number of patients at randomization*: 21 - *Number of patients at outcome*: 21   **Included criteria:** All women admitted between 24 and 31.6 weeks’ gestation with preterm labor  **Excluded criteria:** Vaginal bleeding due to placental abruption or placental tear, suspected major fetal anomalies, severe intrauterine growth restriction(IUGR,-3rd percentile), suspected twin-twin transfusion syndrome or discordant twin growth and maternal drug abuse  **Pretreatment:** Comparable baseline characteristics |
| **Interventions** | **Intervention Characteristics**  Delayed cord clamping   - *Description of procedure*: DCC as between 30 and 45 s until the first clamp was placed on the umbilical cord - *Time to cord clamping*: 30-45 s   Immediate cord clamping   - *Description of procedure*: ICC was considered as < 10 s - *Time to cord clamping*: 0-10 s |
| **Outcomes** | *Retinopathy of prematurity*   - **Outcome type**: Dichotomous Outcome   *IVH*   - **Outcome type**: Dichotomous Outcome   *Necrotising enterocolitis*   - **Outcome type**: Dichotomous Outcome   *Number of blood transfusion until hospital discharge*   - **Outcome type**: Continuous Outcome   *Concentrations of total hemoglobin (Hb)*   - **Outcome type**: Continuous Outcome   *Late sepsis until hospital discharge*   - **Outcome type**: Dichotomous Outcome   *Patent ductus arteriosus (pharmacological treatment and surgical ligation)*   - **Outcome type**: Dichotomous Outcome   *Duration in days of respiratory support*   - **Outcome type**: Continuous Outcome   *Duration in days of supplemental oxygen requirement*   - **Outcome type**: Continuous Outcome |
| **Identification** | **Sponsorship source:** Not reported  **Country:** Turkey  **Setting:** Single center  **Comments:**  **Authors name:** Gokmen  **Institution:** Division of Neonatology, Department of Pediatrics, Baskent University School of Medicine  **Email:** zggokmen@yahoo.com  **Address:** Ankara, Turkey |
| **Notes** |  |

**Risk of bias table**

| **Bias** | **Authors' judgement** | **Support for judgement** |
| --- | --- | --- |
| Random sequence generation (selection bias) | Unclear risk | Judgement Comment: No information |
| Allocation concealment (selection bias) | Unclear risk | Judgement Comment: No information |
| Blinding of participants and personnel (performance bias) | High risk | Quote: "was not possible to mask the trial assignment to the neonatal or obstetric team in the delivery room. However," |
| Blinding of outcome assessment (detection bias) | Low risk | Quote: "However, the neonatal staff was asked not to record the time in the chart (only randomization code number), and this information was not available to the staff in the NICU." |
| Incomplete outcome data (attrition bias) | Low risk | Judgement Comment: Data appears to be complete. Attrition and exclusions were explained and accounted for. |
| Selective reporting (reporting bias) | Unclear risk | Judgement Comment: No protocol found |
| Other bias | Low risk | Judgement Comment: None |

**Hosono 2008**

| **Methods** | **Study design:** Randomized controlled trial  **Study grouping:** Parallel group |
| --- | --- |
| **Participants** | **Baseline Characteristics**  Cord milking   - *Gestational age (SD) (weeks)*: 27.0 (1.5) - *Birth weight (SD) (grams)*: 836 (223) - *Number of patients at randomization*: 20 - *Number of patients at outcome*: 20   Immediate cord clamping   - *Gestational age (SD) (weeks)*: 26.6 (1.2) - *Birth weight (SD) (grams)*: 846 (171) - *Number of patients at randomization*: 20 - *Number of patients at outcome*: 20   **Included criteria:** VLBW infants born between 24 to 28 weeks’ gestation  **Excluded criteria:** multiple births major congenital anomalies or chromosomal anomalies and hydrops fetalis  **Pretreatment:** Comparable baseline characteristics |
| **Interventions** | **Intervention Characteristics**  Cord milking   - *Description of procedure*: Infants were placed at or below the level of the placenta, and about 20 cm of the umbilical cord was vigorously milked towards the umbilicus two to three times before clamping the cord. The milking speed was about 20 cm per 2 seconds. - *Time to cord clamping*: Not reported   Immediate cord clamping   - *Description of procedure*: Umbilical cord was clamped immediately - *Time to cord clamping*: Not reported |
| **Outcomes** | *All-cause mortality during initial hospitalization*   - **Outcome type**: Dichotomous Outcome |
| **Identification** | **Sponsorship source:** Not reported  **Country:** Japan  **Setting:** Single center  **Comments:**  **Authors name:** Hosono  **Institution:** Department of Pediatrics and Child Health, Nihon University School of Medicine  **Email:** hosonos@med.nihon-u.ac.jp  **Address:** Oyaguchi Itabashi, Tokyo, Japan |
| **Notes** |  |

**Risk of bias table**

| **Bias** | **Authors' judgement** | **Support for judgement** |
| --- | --- | --- |
| Random sequence generation (selection bias) | Low risk | Judgement Comment: No information |
| Allocation concealment (selection bias) | Low risk | Quote: "serially numbered opaque envelopes" |
| Blinding of participants and personnel (performance bias) | High risk | Judgement Comment: Due to the nature of the study |
| Blinding of outcome assessment (detection bias) | High risk | Judgement Comment: No information |
| Incomplete outcome data (attrition bias) | Low risk | Judgement Comment: Data appears to be complete. Attrition and exclusions were explained and accounted for |
| Selective reporting (reporting bias) | Unclear risk | Judgement Comment: No protocol found |
| Other bias | Low risk | Judgement Comment: None |

**Josephsen 2014**

| **Methods** | **Study design:** Randomized controlled trial  **Study grouping:** Parallel group |
| --- | --- |
| **Participants** | **Baseline Characteristics**  Cord milking   - *Gestational age (SD) (weeks)*: 26.5 (1.4) - *Birth weight (SD) (grams)*: 914.6 (208.6) - *Number of patients at randomization*: 13 - *Number of patients at outcome*: 13   Immediate cord clamping   - *Gestational age (SD) (weeks)*: 26.1 (0.9) - *Birth weight (SD) (grams)*: 809.6 (178.7) - *Number of patients at randomization*: 13 - *Number of patients at outcome*: 12   **Included criteria:** Singletons born between 24 and 27 6/7 weeks gestation  **Excluded criteria:** Multiple gestation, congenital abnormalities, hydrops fetalis, and known fetal anemia  **Pretreatment:** Comparable baseline characteristics |
| **Interventions** | **Intervention Characteristics**  Cord milking   - *Description of procedure*: Actively milking 18 cm of the umbilical cord to the umbilicus 3 times by a limited group of physicians trained in this specific technique - *Time to cord clamping*: Not reported   Immediate cord clamping   - *Description of procedure*: Routine immediate cord clamping - *Time to cord clamping*: Not reported |
| **Outcomes** | *All-cause neonatal mortality (first 28 days)*   - **Outcome type**: Dichotomous Outcome   *All-cause mortality during initial hospitalization*   - **Outcome type**: Dichotomous Outcome   *One-year survival*   - **Outcome type**: Dichotomous Outcome   *Major neurodevelopmental disability*   - **Outcome type**: Dichotomous Outcome   *Retinopathy of prematurity*   - **Outcome type**: Dichotomous Outcome   *IVH*   - **Outcome type**: Dichotomous Outcome   *White matter at term-equivalent MRI abnormalities at term equivalent age*   - **Outcome type**: Dichotomous Outcome   *Bronchopulmonary dysplasia/chronic lung disease*   - **Outcome type**: Dichotomous Outcome   *Necrotising enterocolitis*   - **Outcome type**: Dichotomous Outcome   *Volume in ml of blood withdrawn until hospital discharge*   - **Outcome type**: Continuous Outcome   *Volume in ml of blood transfused until hospital discharge*   - **Outcome type**: Continuous Outcome   *Number of blood transfusions until hospital discharge*   - **Outcome type**: Continuous Outcome   *Need for blood transfusions until hospital discharge*   - **Outcome type**: Dichotomous Outcome   *Concentrations of total hemoglobin (Hb)*   - **Outcome type**: Continuous Outcome   *Concentrations of fetal hemoglobin (Hb F)*   - **Outcome type**: Continuous Outcome   *Late sepsis until hospital discharge*   - **Outcome type**: Dichotomous Outcome   *Patent ductus arteriosus (pharmacological treatment and surgical ligation)*   - **Outcome type**: Dichotomous Outcome   *Duration in days of respiratory support*   - **Outcome type**: Continuous Outcome   *Duration in days of supplemental oxygen requirement*   - **Outcome type**: Continuous Outcome   *Duration in days of hospital stay*   - **Outcome type**: Continuous Outcome   *Composite outcome: Major Developmental Disability*   - **Outcome type**: Dichotomous Outcome   *Poor academic performance*   - **Outcome type**: Dichotomous Outcome   *Impaired Motor skills*   - **Outcome type**: Dichotomous Outcome   *Behavior problem*   - **Outcome type**: Dichotomous Outcome   *Time in minutes to perform the procedure in each study arm*   - **Outcome type**: Continuous Outcome   *Pain score during device insertion*   - **Outcome type**: Continuous Outcome   *Number of skin-breaking procedure associated to blood testing, insertion and repositioning of the device*   - **Outcome type**: Dichotomous Outcome   *Skin/soft tissue injury associated to blood testing, insertion and repositioning of the device*   - **Outcome type**: Dichotomous Outcome   *Site infection associated to blood testing, insertion and repositioning of the device*   - **Outcome type**: Dichotomous Outcome   *Thrombosis*   - **Outcome type**: Dichotomous Outcome |
| **Identification** | **Sponsorship source:** Not reported  **Country:** United States of America  **Setting:** Single center  **Comments:** Abstract only  **Authors name:** Josephsen  **Institution:** Saint Louis University, Department of Pediatrics, Division of Neonatology  **Email:** jjosephs@slu.edu  **Address:** Saint Louis, MO, USA |
| **Notes** |  |

**Risk of bias table**

| **Bias** | **Authors' judgement** | **Support for judgement** |
| --- | --- | --- |
| Random sequence generation (selection bias) | Unclear risk | Judgement Comment: Not reported |
| Allocation concealment (selection bias) | Unclear risk | Judgement Comment: Not reported |
| Blinding of participants and personnel (performance bias) | High risk | Judgement Comment: due to the nature of the study |
| Blinding of outcome assessment (detection bias) | High risk | Judgement Comment: Not reported |
| Incomplete outcome data (attrition bias) | Unclear risk | Judgement Comment: Unclear information |
| Selective reporting (reporting bias) | Unclear risk | Judgement Comment: Protocol found. Hard to judge as this is only a substudy and the larger study has yet to be published. Hard to judge the results from the follow-up that are mentioned in the protocol and in the abstract |
| Other bias | Low risk | Judgement Comment: None |

**Katheria 2014**

| **Methods** | **Study design:** Randomized controlled trial  **Study grouping:** Parallel group |
| --- | --- |
| **Participants** | **Baseline Characteristics**  Cord milking   - *Gestational age (SD) (weeks)*: 28 (2) - *Birth weight (SD) (grams)*: 1170 (356) - *Number of patients at randomization*: 30 - *Number of patients at outcome*: 30   Immediate cord clamping   - *Gestational age (SD) (weeks)*: 28 (3) - *Birth weight (SD) (grams)*: 1131 (396) - *Number of patients at randomization*: 30 - *Number of patients at outcome*: 30   **Included criteria:** Pregnant women dated by their earliest ultrasound or last menstrual period at <32 weeks’ gestation  **Excluded criteria:** Monochorionic multiples, incarcerated mothers, placenta previa, concern for abruptions, or refusal to perform the intervention by the obstetrician (OB)  **Pretreatment:** Comparable baseline characteristics |
| **Interventions** | **Intervention Characteristics**  Cord milking   - *Description of procedure*: UCM was performed by having the delivering OB hold the infant below the mother’s introitus at vaginal delivery or below the level of the incision at cesarean delivery and having the assistant (the second OB) milk about 20 cm of umbilical cord over 2 seconds (counting aloud), repeating 2 additional times as described previously. - *Time to cord milking/clamping (SD)*: 17 (8)   Immediate cord clamping   - *Description of procedure*: Not reported - *Time to cord milking/clamping (SD)*: 14 (9) |
| **Outcomes** | *All-cause mortality during initial hospitalization*   - **Outcome type**: Dichotomous Outcome   *IVH*   - **Outcome type**: Dichotomous Outcome   *Need for blood transfusions until hospital discharge*   - **Outcome type**: Dichotomous Outcome   *Concentrations of total hemoglobin (Hb)*   - **Outcome type**: Continuous Outcome   *Patent ductus arteriosus (pharmacological treatment and surgical ligation)*   - **Outcome type**: Dichotomous Outcome   *Duration in days of respiratory support*   - **Outcome type**: Continuous Outcome   *Duration in days of supplemental oxygen requirement*   - **Outcome type**: Continuous Outcome   *Bronchopulmonary dysplasia*   - **Outcome type**: Dichotomous Outcome |
| **Identification** | **Sponsorship source:** This study was funded by the University of California, San Diego with no external funding sources  **Country:** United States of America  **Setting:** Single center  **Comments:**  **Authors name:** Katheria  **Institution:** Division of Neonatology, UCSD Medical Center, University of California San Diego  **Email:** anup.katheria@sharp.com  **Address:** San Diego, CA, USA |
| **Notes** |  |

**Risk of bias table**

| **Bias** | **Authors' judgement** | **Support for judgement** |
| --- | --- | --- |
| Random sequence generation (selection bias) | Unclear risk | Judgement Comment: Not reported |
| Allocation concealment (selection bias) | Low risk | Quote: "Infants were randomized by the placement of their information in opaque, sealed envelopes immediately before delivery" |
| Blinding of participants and personnel (performance bias) | High risk | Quote: "The OBs were made aware of the randomization by the neonatology team before delivery of the infant. " |
| Blinding of outcome assessment (detection bias) | Unclear risk | Judgement Comment: The investigators performing echocardiograms were not involved in the randomization or the recording of the intervention. Unclear for the other outcomes |
| Incomplete outcome data (attrition bias) | Low risk | Judgement Comment: Data appears to be complete. Attrition and exclusions were explained and accounted for. |
| Selective reporting (reporting bias) | Unclear risk | Judgement Comment: Protocol found. Authors planned on assessing neurodevelopmental outcomes for infants aged 18-36 months. These outcomes are not reported in the paper but would likely be reported in an upcoming paper. |
| Other bias | Low risk | Judgement Comment: No definition of ICC |

**Kazemi 2017**

| **Methods** | **Study design:** Randomized controlled trial  **Study grouping:** Parallel group |
| --- | --- |
| **Participants** | **Baseline Characteristics**  Delayed cord clamping   - *Gestational age (SD) (weeks)*: 30.1(1.7) - *Birth weight (SD) (grams)*: 1260.8(213.4) - *Number of patients at randomization*: 35 - *Number of patients at outcome*: 35   Immediate cord clamping   - *Gestational age (SD) (weeks)*: 29.8 (1.8) - *Birth weight (SD) (grams)*: 1241.2 (233.7) - *Number of patients at randomization*: 35 - *Number of patients at outcome*: 35   **Included criteria:** Preterm infants with gestational age of less than 32 weeks, weighing fewer than 1500 grams at birth, born by C-section who did not require advanced resuscitation and showed no congenital anomaly in physical examination.  **Excluded criteria:** Maternal use of medications affecting the coagulation system, birth asphyxia, need for resuscitation at the time of delayed clamping, birth trauma, need for advanced resuscitation, infants from multiple gestation or breech presentation as the cause of C-section, and mother’s systemic diseases such as preeclampsia, hyper-tension, and uncontrolled diabetes.  **Pretreatment:** Comparable baseline characteristics |
| **Interventions** | **Intervention Characteristics**  Delayed cord clamping   - *Description of procedure*: The umbilical cord clamping was delayed by 30 - 45 seconds and the infants were put under sterile conditions 25 - 30 cm below the mother, before umbilical cord was clamped - *Time to cord clamping*: 30-45 s   Immediate cord clamping   - *Description of procedure*: The umbilical cord was clamped immediately in fewer than 10 seconds - *Time to cord clamping*: 0-10 s |
| **Outcomes** | *IVH*   - **Outcome type**: Dichotomous Outcome   *White matter at term-equivalent MRI abnormalities at term equivalent age*   - **Outcome type**: Dichotomous Outcome - **Notes**: there is an inconsistency in data reportsee Table 3 - from the text in results section I understand that "grade" in the table refers to PVL. Then in the table we have 2 cases of PVL reported, but in the description of its grade we can find grade 1 - 1case and grade 2 - 3 cases, that gives 4 in total. Strangely they also did not report grade 3 of PVL. |
| **Identification** | **Sponsorship source:** Not reported  **Country:** Iran  **Setting:** Single center  **Comments:**  **Authors name:** Kazemi  **Institution:** Non-Communicable Pediatric Diseases Research Center, Health Research Institute, Babol University of Medical Sciences  **Email:** matia.mojaveri@yahoo.com  **Address:** Babol, Iran |
| **Notes** |  |

**Risk of bias table**

| **Bias** | **Authors' judgement** | **Support for judgement** |
| --- | --- | --- |
| Random sequence generation (selection bias) | Unclear risk | Judgement Comment: Not reported |
| Allocation concealment (selection bias) | Unclear risk | Judgement Comment: Not reported |
| Blinding of participants and personnel (performance bias) | High risk | Judgement Comment: Not reported |
| Blinding of outcome assessment (detection bias) | High risk | Judgement Comment: Not reported |
| Incomplete outcome data (attrition bias) | Low risk | Judgement Comment: Data appears to be complete. Attrition and exclusions were explained and accounted for. |
| Selective reporting (reporting bias) | High risk | Judgement Comment: More outcomes are reported than on the protocol; "survival" is not reported. The protocol was registered while recruiting |
| Other bias | Low risk | Judgement Comment: None |

**Kugelman 2007**

| **Methods** | **Study design:** Randomized controlled trial  **Study grouping:** Parallel group |
| --- | --- |
| **Participants** | **Baseline Characteristics**  Delayed cord clamping   - *Gestational age (SD) (weeks)*: 30.3(1.78) - *Birth weight (SD) (grams)*: 1233.68 (247.3) - *Number of patients at randomization*: 19 - *Number of patients at outcome*: 19   Immediate cord clamping   - *Gestational age (SD) (weeks)*: 29.78 (1.95) - *Birth weight (SD) (grams)*: 1328.35 (308.2) - *Number of patients at randomization*: 17 - *Number of patients at outcome*: 17   **Included criteria:** Gestational age between 24 to 34 and 6/7 weeks assessed by the obstetrical team from dating of last menstrual period and/or ultrasound, written informed consent from the parents, and agreement by the obstetrician to enroll the patients in the study.  **Excluded criteria:** Parents refused consent, vaginal bleeding due to placenta previa or abruption or placental tear, fetus suspected of having a major anomaly, severe intrauterine growth restriction (IUGR; < 3%), maternal gestational diabetes treated with insulin, suspected twin–twin transfusion syndrome or discordant twins (cautious definition of estimated weight difference by fetal ultrasound of > 20%, even without monozygosity), and maternal drug abuse  **Pretreatment:** Comparable baseline characteristics |
| **Interventions** | **Intervention Characteristics**  Delayed cord clamping   - *Description of procedure*: DCC was between 30 to 45 seconds at the time the first clamp was placed on the umbilical cord. Obstetrician held the neonate in a dry blanket or towel and lower the baby as much as possible without creating tension on the cord while the neonate was still attached to the placental circulation. The goal was for the attendant to hold the neonate ~20 to 30 cm below the mother’s introitus at vaginal delivery or below the level of the incision at CS - *Time to cord clamping*: 30-45 s   Immediate cord clamping   - *Description of procedure*: ICC was considered < 10 seconds - *Time to cord clamping*: 0-10 s |
| **Outcomes** | *Patient ductus arteriosus (pharmacological treatment and surgical ligation)*   - **Outcome type**: Dichotomous Outcome   *Necrotising enterocolitis*   - **Outcome type**: Dichotomous Outcome   *Number of blood transfusions until hospital discharge*   - **Outcome type**: Continuous Outcome   *Concentrations of total hemoglobin (Hb)*   - **Outcome type**: Continuous Outcome   *Late sepsis until hospital discharge*   - **Outcome type**: Dichotomous Outcome   *Duration in days of respiratory support*   - **Outcome type**: Continuous Outcome   *Duration in days of* supplemental oxygen requirement   - **Outcome type**: Continuous Outcome |
| **Identification** |  |
| **Notes** | **Sponsorship source:** Not reported  **Country:** Israel  **Setting:** Single center  **Comments:**  **Authors name:** Kugelman  **Institution:** Department of Neonatology, Bnai-Zion Medical Center, The Bruce Rappaport Faculty of Medicine  **Email:** a_kugelman@rambam.health.gov.il  **Address:** Haifa, Israel |

**Risk of bias table**

| **Bias** | **Authors' judgement** | **Support for judgement** |
| --- | --- | --- |
| Random sequence generation (selection bias) | Unclear risk | Judgement Comment: Not reported |
| Allocation concealment (selection bias) | Low risk | Quote: "a system of randomly prepared cards in sealed nontransparent envelopes containing early or late group assignment and kept in the labor unit." |
| Blinding of participants and personnel (performance bias) | Unclear risk | Judgement Comment: Not reported |
| Blinding of outcome assessment (detection bias) | Unclear risk | Judgement Comment: Not reported |
| Incomplete outcome data (attrition bias) | Low risk | Quote: "Out of eligible 173 preterm expected deliveries, 22 were excluded because of exclusion criteria (five neonates with significant IUGR, two neonates because of suspected congenital malformations, eight neonates due to maternal vaginal bleeding, six neonates due to suspected twin– twin discordance or transfusion syndrome, and one neonate due to maternal drug abuse and heavy smoking). There were 151 eligible premature neonates. Twenty-one were not included because of parental refusal to participate, and 65 were not included because of technical limitations (rapid delivery and not enough time to acquire informed consent, or unavailability of staff)."  Judgement Comment: data are presented in accordance with methods section |
| Selective reporting (reporting bias) | Unclear risk | Judgement Comment: No protocol found |
| Other bias | Low risk | Judgement Comment: None |

**March 2013**

| **Methods** | **Study design:** Randomized controlled trial  **Study grouping:** Parallel group |
| --- | --- |
| **Participants** | **Baseline Characteristics**  Cord milking   - *Gestational age (IQR) (weeks)*: 27.0 (25.5–28.1) - *Birth weight (IQR) (grams)*: 755.0 (687.5–980.0) - *Number of patients at randomization*: 56 - *Number of patients at outcome*: 36   Immediate cord clamping   - *Gestational age (IQR) (weeks)*: 26.3 (25.1–27.1) - *Birth weight (IQR) (grams)*: 770.0 (650.0–940.0) - *Number of patients at randomization*: 57 - *Number of patients at outcome*: 39   **Included criteria:** Pregnant women (aged 18 years or older) admitted to our institution at risk for delivering a singleton preterm infant between 24 and 28 completed weeks of gestation  **Excluded criteria:** Antenatally diagnosed major fetal congenital anomaly, known Rh sensitization, hydrops fetalis, known recent maternal exposure to Parvovirus, elevated peak systolic velocity of the fetal middle cerebral artery or clinical suspicion of placental abruption at delivery due to excessive maternal bleeding or uterine hypertonicity  **Pretreatment:** Comparable baseline characteristics |
| **Interventions** | **Intervention Characteristics**  Cord milking   - *Description of procedure*: An extended hand’s width length of cord (from the tip of the thumb to the tip of the pinky finger, 20±2 cm) was used as the standard. Infants in the cord milking group were placed at or below the level of the placenta if delivered vaginally or at the same level as the placenta if delivered by cesarean section, and ~20 cm of the umbilical cord was actively milked towards the umbilicus three times before clamping the cord. - *Time to cord clamping*: Not reported   Immediate cord clamping   - *Description of procedure*: Cord clamped and cut immediately after delivery. - *Time to cord clamping*: Not reported |
| **Outcomes** | *All-cause neonatal mortality (first 28 days)*   - **Outcome type**: Dichotomous Outcome   *Retinopathy of prematurity*   - **Outcome type**: Dichotomous Outcome   *IVH*   - **Outcome type**: Dichotomous Outcome   *Necrotising enterocolitis*   - **Outcome type**: Dichotomous Outcome   *Volume in ml of blood transfused until hospital discharge*   - **Outcome type**: Continuous Outcome   *Number of blood transfusions until hospital discharge*   - **Outcome type**: Continuous Outcome   *Concentrations of total hemoglobin (Hb)*   - **Outcome type**: Continuous Outcome   *Late sepsis until hospital discharge*   - **Outcome type**: Dichotomous Outcome   *Duration in days of respiratory support*   - **Outcome type**: Continuous Outcome |
| **Identification** | **Sponsorship source:** This work was conducted with support from Harvard Catalyst. The Harvard Clinical and Translational Science Center (National Center for Research Resources and the National Center for Advancing Translational Sciences, National Institutes of Health Award 8UL1TR000170-05 and financial contributions from the Harvard University and its affiliated academic health-care centers)  **Country:** United States of America  **Setting:** Single center  **Comments:**  **Authors name:** March  **Institution:** Division of Maternal-Fetal Medicine, Beth Israel Deaconess Medical Center  **Email:** melissamarch@gmail.com  **Address:** Boston, MA, USA |
| **Notes** |  |

**Risk of bias table**

| **Bias** | **Authors' judgement** | **Support for judgement** |
| --- | --- | --- |
| Random sequence generation (selection bias) | Low risk | Quote: "were randomized before delivery to one of two groups using random permuted blocks of 10; a statistician provided the randomization sequence." |
| Allocation concealment (selection bias) | Low risk | Quote: "Serially numbered opaque envelopes contained arm bands identifying whether a patient was assigned to the cord milking or control group. These arm bands were secured" |
| Blinding of participants and personnel (performance bias) | High risk | Quote: "The neonatologists and pediatric support staff were not blinded to treatment assignment given that they were required to be present for the delivery." |
| Blinding of outcome assessment (detection bias) | Low risk | Quote: "However, they were not alerted for study participation or treatment assignment and no notation of study participation was made in the neonate’s chart in order to minimize the possibility that postnatal treatment decisions would be influenced by study participation." |
| Incomplete outcome data (attrition bias) | Low risk | Judgement Comment: Data appears to be complete. Attrition and exclusions were explained and accounted for. |
| Selective reporting (reporting bias) | Unclear risk | Judgement Comment: No protocol found. |
| Other bias | Low risk | Judgement Comment: None |

**Mercer 2003**

| **Methods** | **Study design:** Randomized controlled trial  **Study grouping:** Parallel group |
| --- | --- |
| **Participants** | **Baseline Characteristics**  Delayed cord clamping   - *Gestational age (SD) (weeks)*: 28 (2) - *Birth weight (SD) (grams)*: 1064 (290) - *Number of patients at randomization*: 16 - *Number of patients at outcome*: 16   Immediate cord clamping   - *Gestational age (SD) (weeks)*: 27 (2.2) - *Birth weight (SD) (grams)*: 1005 (260) - *Number of patients at randomization*: 16 - *Number of patients at outcome*: 16   **Included criteria:** Gestational age between 24 and 31 and 6/7 weeks assessed by the obstetrical team from dating of last menstrual period or ultrasound, singleton pregnancy, the obstetrician agreed to enrolment into the study and parents gave written consent.  **Excluded criteria:** If the obstetrician or parents refused consent, if there was intent to withhold or withdraw care, or if the women had diagnoses of placenta previa or abruption, bleeding, or a fetus with a major anomaly  **Pretreatment:** Comparable baseline characteristics |
| **Interventions** | **Intervention Characteristics**  Delayed cord clamping   - *Description of procedure*: The obstetrician was instructed to hold the infant in a blanket or towel and lower the baby as much as possible without creating tension on the cord while the infant was still attached to the placental circulation. The goal was for the attendant to hold the infant approximately 10 to15 in below the mother’s introitus at vaginal delivery or 10 to 15 in below the level of the incision at Cesarean section. After delivery of the buttocks, the PI counted out the time elapsed in 10-second intervals to the obstetrician. The cord clamping interval ended at 30 to 45 seconds when the obstetrician placed the first clamp on the umbilical cord. - *Time to cord clamping*: 32 (12) s   Immediate cord clamping   - *Description of procedure*: The obstetrician clamped the umbilical cord between 5 and 10 seconds after delivery of the buttocks and transferred the baby to the neonatology staff for routine care of the infant. - *Time to cord clamping*: 6.2 (3) s |
| **Outcomes** | *All-cause mortality during initial hospitalization*   - **Outcome type**: Dichotomous Outcome   *IVH*   - **Outcome type**: Dichotomous Outcome   *Necrotising enterocolitis*   - **Outcome type**: Dichotomous Outcome   *Volume in ml of blood transfused until hospital discharge*   - **Outcome type**: Continuous Outcome   *Concentrations of total hemoglobin (Hb)*   - **Outcome type**: Continuous Outcome   *Duration in days of supplemental oxygen requirement*   - **Outcome type**: Continuous Outcome   *Duration in days of hospital stay*   - **Outcome type**: Continuous Outcome |
| **Identification** | **Sponsorship source:** Sigma Theta Tau, Epsilon Chapter; University of Rhode Island Foundation and College of Nursing  **Country:** United States of America  **Setting:** Single center  **Comments:**  **Authors name:** Mercer  **Institution:** College of Nursing, University of Rhode Island  **Email:** Not reported  **Address:** Kingston, RI, USA |
| **Notes** |  |

**Risk of bias table**

| **Bias** | **Authors' judgement** | **Support for judgement** |
| --- | --- | --- |
| Random sequence generation (selection bias) | Unclear risk | Judgement Comment: Not reported |
| Allocation concealment (selection bias) | Low risk | Quote: "sealed nontransparent envelopes" |
| Blinding of participants and personnel (performance bias) | High risk | Judgement Comment: due to the nature of the study |
| Blinding of outcome assessment (detection bias) | Unclear risk | Quote: "However, the neonatal staff was asked not to record the cord clamping interval in the infant’s chart so that this information was not available to staff caring for the infant in the NICU. The subsequent clinical management of the infants was left to the discretion of the neonatologists." |
| Incomplete outcome data (attrition bias) | Low risk | Judgement Comment: data are presented in accordance with methods section |
| Selective reporting (reporting bias) | Unclear risk | Judgement Comment: No protocol found |
| Other bias | Low risk | Judgement Comment: None |

**Mercer 2006**

| **Methods** | **Study design:** Randomized controlled trial  **Study grouping:** Parallel group |
| --- | --- |
| **Participants** | **Baseline Characteristics**  Delayed cord clamping   - *Gestational age (SD) (weeks)*: 28.3(2.1) - *Birth weight (SD) (grams)*: 1175(346) - *Number of patients at randomization*: 36 - *Number of patients at outcome*: 36   Immediate cord clamping   - *Gestational age (SD) (weeks)*: 28.2(2.4) - *Birth weight (SD) (grams)*: 1151(379) - *Number of patients at randomization*: 36 - *Number of patients at outcome*: 36   **Included criteria:** Women admitted between 24 and 31.6 weeks’ gestation with preterm labor  **Excluded criteria:** Obstetrician’s refusal to participate, major congenital anomalies or multiple gestations, intent to withhold care, severe maternal illnesses, or placenta abruption or previa.  **Pretreatment:** Comparable baseline characteristics |
| **Interventions** | **Intervention Characteristics**  Delayed cord clamping   - *Description of procedure*: The obstetrician clamped the cord at 30 to 45seconds and held the infant in a sterile towel or blanket approximately 10 to 15 inches below the mother’s introitus at vaginal delivery or below the level of the incision at cesarean section. Care was taken that no tension or traction was placed on the cord. A stopwatch was used to mark the time when the infant’s buttocks were delivered from the vagina or the uterus (or head if breech), and then the time elapsed was counted out in10-second intervals for the obstetrician. At 30 to 45seconds, the obstetrician clamped and cut the umbilical cord, and the infant was moved to the radiant warmer for care - *Time to cord clamping*: 32.1 (12.6)   Immediate cord clamping   - *Description of procedure*: The obstetrician clamped the umbilical cord immediately (10 seconds) after birth - *Time to cord clamping*: 6.9 (4.3) |
| **Outcomes** | *All-cause mortality during initial hospitalization*   - **Outcome type**: Dichotomous Outcome   *Retinopathy of prematurity*   - **Outcome type**: Dichotomous Outcome   *IVH*   - **Outcome type**: Dichotomous Outcome   *Bronchopulmonary dysplasia/chronic lung disease*   - **Outcome type**: Dichotomous Outcome   *Necrotising enterocolitis*   - **Outcome type**: Dichotomous Outcome   *Volume in ml of blood withdrawn until hospital discharge*   - **Outcome type**: Continuous Outcome   *Volume in ml of blood transfused until hospital discharge*   - **Outcome type**: Continuous Outcome   *Need for blood transfusions until hospital discharge*   - **Outcome type**: Dichotomous Outcome   *Concentrations of total hemoglobin (Hb)*   - **Outcome type**: Continuous Outcome   *Late sepsis until hospital discharge*   - **Outcome type**: Dichotomous Outcome |
| **Identification** | **Sponsorship source:** Supported by National Institutes of Health, National Institute for Nursing Research grantK23 NR00078.  **Country:** United States of America  **Setting:** Single center  **Comments:** Data taken from three papers: Delayed Cord Clamping in Very Preterm Infants Reduces the Incidence of Intraventricular Hemorrhage and Late-Onset Sepsis: A Randomized, Controlled Trial, Seven-month Developmental Outcomes of Very Low Birth Weight Infants Enrolled in a Randomized Controlled Trial of Delayed Versus Immediate Cord Clamping, Hemodynamic Effects of Delayed Cord Clamping in Premature Infants  **Authors name:** Mercer  **Institution:** College of Nursing, University of Rhode Island  **Email:** jmercer@uri.edu  **Address:** Kingston, Rhode Island, USA |
| **Notes** |  |

**Risk of bias table**

| **Bias** | **Authors' judgement** | **Support for judgement** |
| --- | --- | --- |
| Random sequence generation (selection bias) | Low risk | Quote: "A statistician who was not involved in the trial developed a computer-generated random number system. Block-stratiﬁed randomization was used to assign the intervention to the subjects above and below 28 weeks with a prespeciﬁed equal probability to avoid unequal numbers of participants in each gestational age group." |
| Allocation concealment (selection bias) | Low risk | Quote: "Two sets of cards labeled for randomization were enclosed in sequenced, opaque envelopes containing group assignment and kept in the labor unit." |
| Blinding of participants and personnel (performance bias) | High risk | Judgement Comment: due to the nature of the study |
| Blinding of outcome assessment (detection bias) | Unclear risk | Quote: "However, staff that attended each birth adhered to the principal investigator’s request not to reveal the infant’s grouping in the infant’s medical charts."  Judgement Comment: Not blinded |
| Incomplete outcome data (attrition bias) | Low risk | Judgement Comment: Data appears to be complete. Attrition and exclusions were explained and accounted for. |
| Selective reporting (reporting bias) | Unclear risk | Judgement Comment: No protocol found |
| Other bias | Low risk | Judgement Comment: None |

**Mercer 2016**

| **Methods** | **Study design:** Randomized controlled trial  **Study grouping:** Parallel group |
| --- | --- |
| **Participants** | **Baseline Characteristics**  Delayed cord clamping   - *Gestational age (SD) (weeks)*: 28.3 (2) - *Birth weight (SD) (grams)*: 1203 (352) - *Number of patients at randomization*: 104 - *Number of patients at outcome*: 103   Immediate cord clamping   - *Gestational age (SD) (weeks)*: 28.4 (2) - *Birth weight (SD) (grams)*: 1136 (350) - *Number of patients at randomization*: 107 - *Number of patients at outcome*: 105   **Included criteria:** Women with a singleton pregnancy estimated at 24 to 31.6 weeks gestation by obstetrical evaluation.  **Excluded criteria:** Multiple gestation, prenatally diagnosed major congenital anomalies, severe or multiple maternal illnesses, and mothers who were at risk for loss to follow-up.  **Pretreatment:** Comparable baseline characteristics |
| **Interventions** | **Intervention Characteristics**  Delayed cord clamping   - *Description of procedure*: The obstetrician placed the infant in a sterile warm towel or blanket and held the infant approximately 10–15 inches below the mother’s introitus at vaginal delivery or below the level of the placenta at cesarean delivery. Care was taken to avoid traction on the cord. Suctioning was at the discretion of the obstetrician. The research nurse, using a stopwatch, counted out the time elapsed in ten second intervals to the obstetrician. At 30 to 45 seconds, the obstetrician was asked to milk the infant’s cord once then clamp and cut the umbilical cord. If unable to carry out the DCC protocol as planned, the cord was milked quickly 2 to 3 times before clamping when possible (n = 11). In the event that the timing of the cord clamping was less than 30 seconds with no cord milking and the baby was randomized to the DCC group, a protocol violation report was completed and the infant remained in the DCC group for primary intention-to-treat analyses (n = 15 - *Time to cord clamping*: 32 (16) s   Immediate cord clamping   - *Description of procedure*: Routine cord clamping in fewer than 10 seconds - *Time to cord clamping*: 6.6 (6) |
| **Outcomes** | *All-cause mortality during initial hospitalization*   - **Outcome type**: Dichotomous Outcome   *IVH*   - **Outcome type**: Dichotomous Outcome   *Bronchopulmonary dysplasia/chronic lung disease*   - **Outcome type**: Dichotomous Outcome   *Necrotising enterocolitis*   - **Outcome type**: Dichotomous Outcome   *Concentrations of total hemoglobin (Hb)*   - **Outcome type**: Continuous Outcome   *Late sepsis until hospital discharge*   - **Outcome type**: Dichotomous Outcome   *Impaired Motor skills*   - **Outcome type**: Dichotomous Outcome |
| **Identification** | **Sponsorship source:** The main study was funded by the National Institute for Nursing Research (RO1 NR100015), and the 18–22 month follow-up was funding by the Thrasher Research Fund (9185).  **Country:** United States of America  **Setting:** Single center  **Comments:**  **Authors name:** Mercer  **Institution:** University of Rhode Island  **Email:** jmercer@uri.edu  **Address:** Kingston, RI, USA |
| **Notes** |  |

**Risk of bias table**

| **Bias** | **Authors' judgement** | **Support for judgement** |
| --- | --- | --- |
| Random sequence generation (selection bias) | Unclear risk | Judgement Comment: Not reported |
| Allocation concealment (selection bias) | Unclear risk | Judgement Comment: No indication if the envelopes were opaque |
| Blinding of participants and personnel (performance bias) | High risk | Judgement Comment: due to the nature of the study |
| Blinding of outcome assessment (detection bias) | Low risk | Judgement Comment: Quote: "Staff who attended each birth were asked not to reveal the infant’s grouping in the infant’s medical records. Personnel collecting on-going clinical data and the follow-up staff completing the developmental assessment remained blinded" |
| Incomplete outcome data (attrition bias) | Low risk | Judgement Comment: Data appears to be complete. Attrition and exclusions were explained and accounted for. |
| Selective reporting (reporting bias) | Unclear risk | Judgement Comment: Two protocols found. All outcomes reported. But in paper they mention measuring BPD as a secondary outcome and it is not reported anywhere except in a forest plot. |
| Other bias | Low risk | Judgement Comment: None |

**Nelle 2012**

| **Methods** | **Study design:** Randomized controlled trial  **Study grouping:** Parallel group |
| --- | --- |
| **Participants** | **Baseline Characteristics**  Delayed cord clamping   - *Gestational age (SD) (weeks)*: 29.0 (2) - *Birth weight (SD) (grams)*: 1140 (240) - *Number of patients at randomization*: 19 - *Number of patients at outcome*: 19   Immediate cord clamping   - *Gestational age (SD) (weeks)*: 28.6 (2) - *Birth weight (SD) (grams)*: 1180 (270) - *Number of patients at randomization*: 16 - *Number of patients at outcome*: 16   **Included criteria:** premature infants < 1500g  **Excluded criteria:** not reported  **Pretreatment:** not reported |
| **Interventions** | **Intervention Characteristics**  Delayed cord clamping   - *Description of procedure*: The umbilical cords were clamped after 30 seconds and the infants were placed 30 cm below placenta level - *Time to cord clamping*: >30s   Immediate cord clamping   - *Description of procedure*: The umbilical cords were clamped immediately - *Time to cord clamping*: Not reported |
| **Outcomes** | *Concentrations of total hemoglobin (Hb)*   - **Outcome type**: Continuous Outcome |
| **Identification** | **Sponsorship source:** Not reported  **Country:** Switzerland  **Setting:** Single center  **Comments:**  **Authors name:** Nelle  **Institution:** Division of Neonatology, University Children's Hospital Berne  **Email:** mathias.nelle@insel.ch  **Address:** Berne, Switzerland |
| **Notes** |  |

**Risk of bias table**

| **Bias** | **Authors' judgement** | **Support for judgement** |
| --- | --- | --- |
| Random sequence generation (selection bias) | Unclear risk | Judgement Comment: Not reported |
| Allocation concealment (selection bias) | Unclear risk | Judgement Comment: Not reported |
| Blinding of participants and personnel (performance bias) | High risk | Judgement Comment: due to the nature of the study |
| Blinding of outcome assessment (detection bias) | High risk | Judgement Comment: Not reported |
| Incomplete outcome data (attrition bias) | Unclear risk | Judgement Comment: No report of total hemoglobin in the outcomes |
| Selective reporting (reporting bias) | Unclear risk | Judgement Comment: No protocol found |
| Other bias | Low risk | Judgement Comment: None |

**Oh 2011**

| **Methods** | **Study design:** Randomized controlled trial  **Study grouping:** Parallel group |
| --- | --- |
| **Participants** | **Baseline Characteristics**  Delayed cord clamping   - *Gestational age (SD) (weeks)*: 26 (1.4) - *Birth weight (SD) (grams)*: 854 (222) - *Number of patients at randomization*: 16 - *Number of patients at outcome*: 16   Immediate cord clamping   - *Gestational age (SD) (weeks)*: 26 (1.1) - *Birth weight (SD) (grams)*: 767 (243) - *Number of patients at randomization*: 17 - *Number of patients at outcome*: 17   **Included criteria:** Women with gestational age between 24 0/7 and 27 6/7 weeks singleton pregnancies  **Excluded criteria:** Not reported  **Pretreatment:** Comparable baseline characteristics |
| **Interventions** | **Intervention Characteristics**  Delayed cord clamping   - *Description of procedure*: The infant’s umbilical cord was clamped at 30-45 seconds after delivery of the infant’s presenting part - *Time to cord clamping*: 30-45 s   Immediate cord clamping   - *Description of procedure*: The umbilical cord was clamped < 10 seconds after delivery of the infant’s presenting part - *Time to cord clamping*: 10 s |
| **Outcomes** | *Retinopathy of prematurity*   - **Outcome type**: Dichotomous Outcome   *IVH*   - **Outcome type**: Dichotomous Outcome   *Necrotising enterocolitis*   - **Outcome type**: Dichotomous Outcome   *Volume in ml of blood withdrawn until hospital discharge*   - **Outcome type**: Continuous Outcome   *Volume in ml/kg of blood transfused until hospital discharge*   - **Outcome type**: Continuous Outcome   *Concentrations of total hemoglobin (Hb)*   - **Outcome type**: Continuous Outcome   *Late sepsis until hospital discharge*   - **Outcome type**: Dichotomous Outcome   *Patent ductus arteriosus (pharmacological treatment and surgical ligation)*   - **Outcome type**: Dichotomous Outcome   *Need for blood transfusions until hospital discharge*   - **Outcome type**: Dichotomous Outcome |
| **Identification** | **Sponsorship source:** The National Institutes of Health and the Eunice Kennedy Shriver National Institute of Child Health and Human Development (NICHD) provided grant support for the Neonatal Research Network’s Delayed Cord Clamping Study  **Country:** United States of America  **Setting:** Multi center  **Comments:**  **Authors name:** Oh  **Institution:** Department of Pediatrics, Women and Infants’ Hospital  **Email:** woh@wihri.org  **Address:** Providence, RI, USA |
| **Notes** |  |

**Risk of bias table**

| **Bias** | **Authors' judgement** | **Support for judgement** |
| --- | --- | --- |
| Random sequence generation (selection bias) | Unclear risk | Judgement Comment: Not reported |
| Allocation concealment (selection bias) | Unclear risk | Judgement Comment: Not reported |
| Blinding of participants and personnel (performance bias) | High risk | Judgement Comment: due to the nature of the study |
| Blinding of outcome assessment (detection bias) | High risk | Judgement Comment: Not reported |
| Incomplete outcome data (attrition bias) | Unclear risk | Judgement Comment: Except for BPD, data appears to be complete, attrition and exclusions were explained and accounted for. |
| Selective reporting (reporting bias) | Unclear risk | Judgement Comment: No protocol found |
| Other bias | Low risk | Judgement Comment: None |

**Prescott 2014**

| **Methods** | **Study design:** Randomized controlled trial  **Study grouping:** Parallel group |
| --- | --- |
| **Participants** | **Baseline Characteristics**  Blood sampling from the umbilical cord or from the placenta   - *Gestational age (SD) (weeks)*: 28 (3) - *Birth weight (SD) (grams)*: 1021 (291) - *Number of patients at randomization*: 24 - *Number of patients at outcome*: 17-24 (7 infants less at outcome than at randomization, unclear which group)   Blood sampling from the infant   - *Gestational age (SD) (weeks)*: 27 (3) - *Birth weight (SD) (grams)*: 1025.5 (308) - *Number of patients at randomization*: 20 - *Number of patients at outcome*: 13-20 (7 infants less at outcome than at randomization, unclear which group)   **Included criteria:** Participants were infants born less than 30 weeks’ gestation or whose birthweight was less than 1200 grams.  **Excluded criteria:** There were no specific exclusion criteria, however infants transferred out of participating institutions during the first week of life were excluded from analysis.  **Pretreatment:** |
| **Interventions** | **Intervention Characteristics**  Blood sampling from the umbilical cord or from the placenta   - *Description of procedure*: The experimental arm had admission lab tests drawn from the umbilical cord blood.   Blood sampling from the infant   - *Description of procedure*: The control arm had admission lab tests drawn from the infant. |
| **Outcomes** | *Retinopathy of prematurity*   - **Outcome type**: Dichotomous Outcome   *IVH*   - **Outcome type**: Dichotomous Outcome   *Volume in ml of blood transfused until hospital discharge*   - **Outcome type**: Continuous Outcome   *Number of blood transfusions until hospital discharge*   - **Outcome type**: Continuous Outcome   *Concentrations of total hemoglobin (Hb)*   - **Outcome type**: Continuous Outcome |
| **Identification** | **Sponsorship source:** San Antonio Military Medical Center IRB  **Country:** United States of America  **Setting:** 3 military treatment facilities in level 3 NICUs  **Comments:**  **Authors name:** Alicia Prescott  **Institution:** San Antonio Military Medical Center IRB  **Email:** Telephone number: 858 - 232- 8459  **Address:** |
| **Notes** |  |

**Risk of bias table**

| **Bias** | **Authors' judgement** | **Support for judgement** |
| --- | --- | --- |
| Random sequence generation (selection bias) | Low risk | Judgement Comment: Quote: "Competitive enrollment occurred across all participating sites utilizing simple randomization at a 1:1 allocation ratio." |
| Allocation concealment (selection bias) | Low risk | Judgement Comment: Quote: “Randomization schedule was provided via closed envelopes by the 59^th^ Medical Wing statistician for dissemination to each of the participating sites.” |
| Blinding of participants and personnel (performance bias) | High risk | Judgement Comment: No mention of blinding the interventions in the method. However, the authors do state in the limitations of the study that, quote: "Weaknesses include the current limited enrollment and inability to blind randomization to the providers caring for the infant", suggesting blinding of the personnel could not be done. |
| Blinding of outcome assessment (detection bias) | Unclear risk | Judgement Comment: Some efforts to blind outcome assessors were mentioned in the study. Quote: "The presence and severity of intraventricular hemorrhage (IVH) was interpreted by a pediatric radiologist who was blinded to the infants’ randomization." However, it is unclear whether the remaining outcomes were blinded. |
| Incomplete outcome data (attrition bias) | High risk | Judgement Comment: For primary analysis, an ITT approach was used. Quote: "The primary analytic approach was based on intention to treat." However, it is unclear if this approach has been used for all outcomes as the number of participants included in each outcome is not reported. Quote: "There were no specific exclusion criteria, however infants transferred out of participating institutions during the first week of life were excluded from analysis." – this would mean that participants might have been excluded from the stated intention-to-treat analysis. Quote: "Only 37 of the 44 infants had CBCs obtained on day of life 7. Five were not obtained by the ordering provider at the correct time. Two patients died prior to day of life 7." – unclear which group they belonged to. |
| Selective reporting (reporting bias) | High risk | Judgement Comment: Protocol for the ongoing RCT available at https://clinicaltrials.gov/ct2/show/NCT02103296. Performing this interim analysis is not mentioned there, nor do the authors mention the protocol in this publication. Composite outcome of ROP, BPD, IVH or death not present in protocol. Also does not present separate data on BPD or death in outcomes, only as a part of the composite. |
| Other bias | High risk | Judgement Comment: Interim analysis of ongoing RCT with a total sample size of 225 infants. At the moment data outcomes are available only for a minority (44) of these 225 infants. "A total of 70 women were admitted at less than 30 weeks’ gestation to labor and delivery at one of three participating institutions." This implies that only data available from 1 of the 3 centers were included in the study. Further data from the other centers might change the results. 8 infants (33%) in the intervention group compared to 5 infants (25%) in the control group received DCC. Quote: "There was a slightly higher percentage of delayed cord clamping in the treatment group, however it was not statistically significant." |

**Rabe 2000**

| **Methods** | **Study design:** Randomized controlled trial  **Study grouping:** Parallel group |
| --- | --- |
| **Participants** | **Baseline Characteristics**  Delayed cord clamping   - *Gestational age (SD) (weeks)*: 30.01 (1.57) - *Birth weight (SD) (grams)*: 1185 (394) - *Number of patients at randomization*: 20 - *Number of patients at outcome*: 19   Immediate cord clamping   - *Gestational age (SD) (weeks)*: 29.48 (1.96) - *Birth weight (SD) (grams)*: 1080 (340) - *Number of patients at randomization*: 20 - *Number of patients at outcome*: 20   **Included criteria:** Single preterm infants of <33 weeks’ gestation  **Excluded criteria:** Rhesus incompatibility, fetal hydrops, congenital fetal anomalies, Apgar <3 at 0 min1thus allowing the obstetrician to stop the delayed cord clamping if the infant was deteriorating) and multiple pregnancies  **Pretreatment:** Comparable baseline characteristics |
| **Interventions** | **Intervention Characteristics**  Delayed cord clamping   - *Description of procedure*: If possible the infant was held below the level of the placenta for at least 20 cm beside the mother and the cord was clamped after 45 s - *Time to cord clamping*: 45 s   Immediate cord clamping   - *Description of procedure*: If possible the infant was held below the level of the placenta for at least 20 cm beside the mother and the cord was clamped after 20 s - *Time to cord clamping*: 20 s |
| **Outcomes** | *All-cause neonatal mortality (first 28 days)*   - **Outcome type**: Dichotomous Outcome   *IVH*   - **Outcome type**: Dichotomous Outcome   *Necrotising enterocolitis*   - **Outcome type**: Dichotomous Outcome   *Need for blood transfusions until hospital discharge*   - **Outcome type**: Dichotomous Outcome   *Concentrations of total hemoglobin (Hb)*   - **Outcome type**: Continuous Outcome   *Patent ductus arteriosus (pharmacological treatment and surgical ligation)*   - **Outcome type**: Dichotomous Outcome |
| **Identification** | **Sponsorship source:** Not reported  **Country:** Germany  **Setting:** Single center  **Comments:**  **Authors name:** Rabe  **Institution:** Department of Paediatrics, University Children's Hospital Muenster  **Email:** hrabe@uni-muenster.de  **Address:** Muenster, Germany |
| **Notes** |  |

**Risk of bias table**

| **Bias** | **Authors' judgement** | **Support for judgement** |
| --- | --- | --- |
| Random sequence generation (selection bias) | Unclear risk | Judgement Comment: Not reported |
| Allocation concealment (selection bias) | Low risk | Quote: "The patients were randomised to either 20 s (group 1) or 45 s (group 2) of delayed cord clamping by opening a sealed dark envelope." |
| Blinding of participants and personnel (performance bias) | High risk | Judgement Comment: due to the nature of the study |
| Blinding of outcome assessment (detection bias) | Unclear risk | Quote: "Efforts were made to blind the study. Trial participation was entered into the infant's notes, but information about the actual cord clamping time was recorded separately." |
| Incomplete outcome data (attrition bias) | Low risk | Judgement Comment: Data appears to be complete. Attrition and exclusions were explained and accounted for. |
| Selective reporting (reporting bias) | Unclear risk | Judgement Comment: No protocol published |
| Other bias | Low risk | Judgement Comment: None |

**Silahli 2018**

| **Methods** | **Study design:** Randomized controlled trial  **Study grouping:** Parallel group |
| --- | --- |
| **Participants** | **Baseline Characteristics**  Cord milking   - *Gestational age (SD) (weeks)*: <=32 weeks - *Birth weight (SD) (grams)*: 1408 (387) - *Number of patients at randomization*: 38 - *Number of patients at outcome*: 38   Immediate cord clamping   - *Gestational age (SD) (weeks)*: <= 32 weeks - *Birth weight (SD) (grams)*: 1454 (394) - *Number of patients at randomization*: 37 - *Number of patients at outcome*: 37   **Included criteria:** Infants with gestational age less than or equal to 32 weeks  **Excluded criteria:** Twin-to-twin transfusion syndrome, fetal and maternal bleeding, dysmorphic features and conotruncal heart disease  **Pretreatment:** Comparable baseline characteristics |
| **Interventions** | **Intervention Characteristics**  Cord milking   - *Description of procedure*: Length of umbilical cord equal to an extended hand's width (from the tip of the thumb to the tip of the pinky [20±2 cm]) was used as the standard. Infants in the UCM group were placed at or below the level of the placenta if delivered vaginally or at the same level as the placenta if delivered by cesarean section (CS), and then~20cm of the umbilical cord was gently milked towards the umbilicus 3 times. - *Time to cord clamping*: Not reported   Immediate cord clamping   - *Description of procedure*: The umbilical cord was clamped within the first 10s of delivery and was immediately thereafter cut. - *Time to cord clamping*: 0-10 s |
| **Outcomes** | *IVH*   - **Outcome type**: Dichotomous Outcome   *Concentrations of total hemoglobin (Hb)*   - **Outcome type**: Continuous Outcome   *Late sepsis until hospital discharge*   - **Outcome type**: Dichotomous Outcome   *Patent ductus arteriosus (pharmacological treatment and surgical ligation)*   - **Outcome type**: Dichotomous Outcome   *Duration in days of hospital stay*   - **Outcome type**: Continuous Outcome |
| **Identification** | **Sponsorship source:** Baskent University Research Fund  **Country:** Turkey  **Setting:** Single center  **Comments:**  **Authors name:** Silahli  **Institution:** Baskent University, Konya Education and Research Centre  **Email:** msilahli@gmail.com  **Address:** Konya, Turkey |
| **Notes** |  |

**Risk of bias table**

| **Bias** | **Authors' judgement** | **Support for judgement** |
| --- | --- | --- |
| Random sequence generation (selection bias) | Low risk | Quote: "randomised before delivery to two groups using random permuted blocks of 10; an independent statistician provided the randomisation sequence." |
| Allocation concealment (selection bias) | Low risk | Quote: "Serially-numbered opaque envelopes contained arm bands signifying that a patient was assigned to the UCM or the ECC group." |
| Blinding of participants and personnel (performance bias) | High risk | Judgement Comment: due to the nature of the study |
| Blinding of outcome assessment (detection bias) | Unclear risk | Quote: "Double-blinding ensured that neither the women nor the radiologists knew to which group each woman was assigned. A length"  Judgement Comment: although the neonatologists were not blinded |
| Incomplete outcome data (attrition bias) | Unclear risk | Judgement Comment: no exact report on mortality in the study, even if it is mentioned as an outcome |
| Selective reporting (reporting bias) | Unclear risk | Judgement Comment: No protocol published |
| Other bias | Low risk | Judgement Comment: None |

**Tarnow Mordi 2018**

| **Methods** | **Study design:** Randomized controlled trial  **Study grouping:** Parallel group |
| --- | --- |
| **Participants** | **Baseline Characteristics**  Delayed cord clamping   - *Gestational age (SD) (weeks)*: 28 (2) - *Birth weight (SD) (grams)*: 1018 (281) - *Number of patients at randomization*: 818 - *Number of patients at outcome*: 784   Immediate cord clamping   - *Gestational age (SD) (weeks)*: 28 (2) - *Birth weight (SD) (grams)*: 1000 (269) - *Number of patients at randomization*: 816 - *Number of patients at outcome*: 782   **Included criteria:** Fetuses delivered before 30 weeks of gestation  **Excluded criteria:** Fetal hemolytic disease, hydrops fetalis, twin–twin transfusion, genetic syndromes, and potentially lethal malformations  **Pretreatment:** Comparable baseline characteristics |
| **Interventions** | **Intervention Characteristics**  Delayed cord clamping   - *Description of procedure*: Clamping 60 seconds or more after delivery, with the infant held as low as possible below the introitus or placenta and without palpation of the cord. - *Time to cord clamping*: >60 s   Immediate cord clamping   - *Description of procedure*: Clamping within 10 seconds after delivery - *Time to cord clamping*: 0-10 s |
| **Outcomes** | *All-cause neonatal mortality (first 28 days)*   - **Outcome type**: Dichotomous Outcome - **Direction**: Lower is bet   *Retinopathy of prematurity*   - **Outcome type**: Dichotomous Outcome - **Direction**: Lower is better   *IVH*   - **Outcome type**: Dichotomous Outcome - **Direction**: Lower is better   *White matter at term-equivalent MRI abnormalities at term equivalent age*   - **Outcome type**: Dichotomous Outcome - **Direction**: Lower is better   *Bronchopulmonary dysplasia/chronic lung disease*   - **Outcome type**: Dichotomous Outcome - **Direction**: Lower is better   *Necrotising enterocolitis*   - **Outcome type**: Dichotomous Outcome - **Direction**: Lower is better   *Late sepsis until hospital discharge*   - **Outcome type**: Dichotomous Outcome - **Direction**: Lower is better - **Notes**: Late is defined as >72h   *Patent ductus arteriosus (pharmacological treatment and surgical ligation)*   - **Outcome type**: Dichotomous Outcome - **Direction**: Lower is better |
| **Identification** | **Sponsorship source:** Supported by the National Health and Medical Research Council (NHMRC) and by the NHMRC Clinical Trials Centre, University of Sydney.  **Country:** Australia  **Setting:** Multi center  **Comments:**  **Authors name:** Tarnow-Mordi  **Institution:** The National Health and Medical Research Council Clinical Trials Centre, University of Sydney  **Email:** williamtm@med.usyd.edu.au  **Address:** Sydney, Australia |
| **Notes** |  |

**Risk of bias table**

| **Bias** | **Authors' judgement** | **Support for judgement** |
| --- | --- | --- |
| Random sequence generation (selection bias) | Low risk | Judgement Comment: The study protocol says: "Randomisation and treatment allocation will be completed by a member of the obstetric, neonatal or midwifery team. Central phone randomisation will be used. This is a computerised interactive voice response system. Randomisation will be performed using an interactive voice response system built by an independent study statistician at the NHMRC Clinical Trials Centre, University of Sydney. All data will be stored securely by the statistical group at the center." Though not explicitly stated, likely that sequence generation was random. |
| Allocation concealment (selection bias) | Low risk | Judgement Comment: The protocol says: "Central phone randomisation will be used. This is a computerised interactive voice response system." |
| Blinding of participants and personnel (performance bias) | High risk | Quote: "We performed an unblinded, randomized, con- trolled trial" |
| Blinding of outcome assessment (detection bias) | High risk | Quote: "For practical reasons, no attempt was made to make staff who were diagnosing these morbidities unaware of the timing of cord clamping."  Judgement Comment: Not blinded |
| Incomplete outcome data (attrition bias) | Low risk | Quote: "Of 1634 fetuses that underwent randomization, 54 were born after 30 weeks, 10 were stillborn, and 4 had consent withdrawn (Fig. 1). A total of 1566 infants were born alive before 30 weeks of gestation and were eligible for evaluation."  Judgement Comment: Data appears to be complete. Attrition and exclusions were explained and accounted for |
| Selective reporting (reporting bias) | Low risk | Judgement Comment: Protocol registered prospectively and published: ACTRN12610000633088 |
| Other bias | Low risk | Judgement Comment: None |

**Thomson 2018**

| **Methods** | **Study design:** Randomized controlled trial  **Study grouping:** Parallel group |
| --- | --- |
| **Participants** | **Baseline Characteristics**  Normal blood sampling   - *Gestational age (SD) (weeks)*: 27.96 (2.1) - *Birth weight (SD) (grams)*: 901 (144) - *Number of patients at randomization*: 12 - *Number of patients at outcome*: 10   Devices to monitor glucose levels, subcutaneous   - *Gestational age (SD) (weeks)*: 27.5 (2.8) - *Birth weight (SD) (grams)*: 823 (282) - *Number of patients at randomization*: 11 - *Number of patients at outcome*: 10   **Included criteria:** Birth weight <1200 g, age <48 hours and written informed parental consent.  **Excluded criteria:** Any baby with a major congenital malformation, any underlying metabolic disorder or if mothers had diabetes mellitus.  **Pretreatment:** |
| **Interventions** | **Intervention Characteristics**  Normal blood sampling   - *Description of procedure*: "Enlite sensors were linked to an Ipro 2 to collect data prospectively but blinded to the clinical team. Standard care aimed to target glucose levels between 2.6 and 10 mmol/L by reduction of dextrose intake or use of sliding scale insulin infusion at the discretion of the clinical team. Sliding scale insulin was considered if BG levels were >10 mmol/L on more than two occasions. The masked CGM data were downloaded on day 7, at the end of the study period."   Devices to monitor glucose levels, subcutaneous   - *Description of procedure*: "Enlite sensors were linked to Paradigm Veo. This allowed real-time viewing of sensor glucose (SG) data, which were used in conjunction with the paper guideline to support clinical management (online supplementary figure). The latter provided simple guidance and was not a rigid algorithm and had not undergone formal in silico testing. The nurses recorded the SG value alongside standard hourly clinical observation, using it to guide the need for BG testing. The guideline prompted review and intervention based on both absolute glucose levels and change." |
| **Outcomes** |  |
| **Identification** | **Sponsorship source:** University of Cambridge and Cambridge University Hospitals NHS Foundation Trust  **Country:** United Kingdom  **Setting:** NICU  **Comments:**  **Authors name:** Lynn Thomson  **Institution:** Department of Paediatrics, University of Cambridge, Cambridge, UK.  **Email:** kb274@cam.ac.uk  **Address:** |
| **Notes** |  |

**Risk of bias table**

| **Bias** | **Authors' judgement** | **Support for judgement** |
| --- | --- | --- |
| Random sequence generation (selection bias) | Low risk | Judgement Comment: Quote: "Randomization using a simple computer randomization programme that included minimization of differences in gestational age and birth weight took place within 48hours of birth." |
| Allocation concealment (selection bias) | Unclear risk | Judgement Comment: No method of concealing the allocation sequence is mentioned. |
| Blinding of participants and personnel (performance bias) | High risk | Judgement Comment: The control group had a masked glucose monitor sensor, supplying some blinding for the participants and parents. The intervention group allowed the personnel real-time viewing of sensor glucose (SG) data, thus were not blinded to the allocated groups. |
| Blinding of outcome assessment (detection bias) | High risk | Judgement Comment: There is no mention of blinding of outcome assessors. |
| Incomplete outcome data (attrition bias) | High risk | Judgement Comment: Assessed as high risk of bias due to no intention-to-treat analysis. 3 out of 23 infants were excluded from the study and data from only 20 infants were analyzed (87%). Quote: "Twenty-three babies were recruited to the pilot study. No CGM data were found at the time of data download in two control babies, and one baby in the intervention group died of a massive pulmonary hemorrhage, within 24 hours of birth. These babies were excluded from the analyses." |
| Selective reporting (reporting bias) | Unclear risk | Judgement Comment: Unclear reporting of outcomes in method section, no protocol available |
| Other bias | Unclear risk | Judgement Comment: In intervention group 30% (3/10) of mothers had chorioamnionitis, whereas none of the mothers in control group; PROM was present in 40% (4/10) in intervention group and in 20% (2/10) in control group. Medtronic provided the CGM system and sensors, but had no role in designing of the study, gathering of data, access to data, preparation of manuscript. |

**Uettwiller 2015**

| **Methods** | **Study design:** Randomized controlled trial  **Study grouping:** Parallel group |
| --- | --- |
| **Participants** | **Baseline Characteristics**  Normal blood sampling   - *Gestational age (MEDIAN [MIN-MAX]) (weeks)*: 29.6 [24.1–34.7] - *Birth weight (MEDIAN [MIN-MAX]) (grams)*: 1014 [579–1485] - *Number of patients at randomization*: 23 - *Number of patients at outcome*: 21   Devices to monitor glucose levels, subcutaneous   - *Gestational age (MEDIAN [MIN-MAX]) (weeks)*: 30.1 [24.4–37] - *Birth weight (MEDIAN [MIN-MAX]) (grams)*: 1000 [620–1485] - *Number of patients at randomization*: 25 - *Number of patients at outcome*: 22   **Included criteria:** "Very low birth weight (VLBW) preterm infants (birth weight under 1500g) who were admitted before 24 hours of life in the Department of Neonatology of the University Hospital of Tours."  **Excluded criteria:** "Serious congenital abnormality, a skin condition that contraindicated continuous glucose monitoring, a transfer to another hospital during the first days of life or an absence of parental agreement."  **Pretreatment:** |
| **Interventions** | **Intervention Characteristics**  Normal blood sampling   - *Description of procedure*: "IGM-group: the intervention(s) to be administered is intermittent capillary glucose testing (IGM-group) associated with a blind-CGMS to detect retrospectively missed hypoglycemia."   Devices to monitor glucose levels, subcutaneous   - *Description of procedure*: "CGM-group: the intervention(s) to be administered is Continuous glucose monitoring with real time glycemia each 5 minutes." |
| **Outcomes** | *Number of skin-breaking procedure associated to blood testing, insertion and repositioning of the device*   - **Outcome type**: Continuous Outcome |
| **Identification** | **Sponsorship source:** University Hospital of Tours  **Country:** France  **Setting:** NICU  **Comments:**  **Authors name:** Florence Uetwiller  **Institution:** Médecine Pédiatrique, CHRU de Tours, Université François Rabelais, Tours, France  **Email:** florence.uettwiller@univ-tours.fr  **Address:** |
| **Notes** |  |

**Risk of bias table**

| **Bias** | **Authors' judgement** | **Support for judgement** |
| --- | --- | --- |
| Random sequence generation (selection bias) | Low risk | Judgement Comment: Quote: "...patients were randomized with stratification according to their birth weight ( 1000g, and 1001–1500g). The random allocation sequence was automatically generated by the statistical software...". |
| Allocation concealment (selection bias) | Low risk | Judgement Comment: Quote: "Two series (one per birth weight category) of numbered and sealed envelopes were created, containing a note with the device to be used for each patient. Each envelope was opened in order after the enrollment of each patient". |
| Blinding of participants and personnel (performance bias) | High risk | Judgement Comment: The personnel and participants could not be blinded due to the alarm which was present in the intervention group but not in the control group. Quote: "In the CGM group, glucose values 60 mg/dl were notified by an alarm..." |
| Blinding of outcome assessment (detection bias) | Unclear risk | Judgement Comment: The principal investigator enrolled the patients and was the only one who had access to the data from the trial. Quote: "All the stored data (RT- and blind-CGMS) were then secondarily transferred to an online secured database and analyzed retrospectively with an access restricted to the principal investigator." It is unclear if the principal investigator also was involved in the care of the neonates or if the treating clinicians were also outcome assessors. |
| Incomplete outcome data (attrition bias) | Low risk | Judgement Comment: Fergusson 2002Out of the infants randomized (n=48), 5 infants were excluded, leaving 43 infants to be analyzed, 21 and 22 in each group. Quote: "Four monitorings failed, two in each group, due to technical problems during the insertion of the sensor, including angulations of the sensor in three cases and minor local bleeding in one case. One patient was excluded directly after the inclusion be- cause of a branchiooculofacial syndrome. Finally, 43 monitorings could be analyzed, 21 and 22." |
| Selective reporting (reporting bias) | Unclear risk | Judgement Comment: Outcomes specified in the methods are reported in the results. Unclear, because of the statement regarding their protocol. Quote: "The present study has been registered with the ClinicalTrials.gov registry (N°NCT01942239; under the name RTCGMS) retrospectively, because registration in a WHO-approved registry was not systematic at the time of the trial design." |
| Other bias | Unclear risk | Judgement Comment: There was higher maternal-fetal infection rate in intervention group (6/25 (=24%)) compared to 0% in control group (p < 0.05). |

**Widness 2005**

| **Methods** | **Study design:** Randomized controlled trial  **Study grouping:** Parallel groups |
| --- | --- |
| **Participants** | **Baseline Characteristics**  Normal blood sampling   - *Gestational age (SD) (weeks)*: 26.0 (1.8) - *Birth weight (SD) (grams)*: 734 (120) - *Number of patients at randomization*: 47 - *Number of patients at outcome*: 42   Devices to reintroduce the blood after analysis   - *Gestational age (SD) (weeks)*: 26.0 (2.0) - *Birth weight (SD) (grams)*: 742 (132) - *Number of patients at randomization*: 46 - *Number of patients at outcome*: 41   **Included criteria:** Preterm infants <24 hours of age, with birth weights between 500 and 1000 g, who had a UAC inserted as clinically indicated.  **Excluded criteria:** Infants were excluded if the maternal prenatal antibody testing indicated the presence of immune hemolytic disease, if the infant was enrolled in a competing research study in which receiving RBC transfusions was an important outcome measure, if the infant had hydrops or a life-threatening congenital anomaly, or if the infant's condition was deemed incompatible with survival (≤22 weeks of gestation, trisomy 13, or trisomy 18).  **Pretreatment:** |
| **Interventions** | **Intervention Characteristics**  Normal blood sampling   - *Description of procedure*: Routine care - Conventional laboratory blood analyses of pH, blood gases, and electrolyte levels in NICU laboratories performed with benchtop analyzers.   Devices to reintroduce the blood after analysis   - *Description of procedure*: Intervention consisting of an in-line, ex vivo, bedside monitor that withdraws blood through an umbilical artery catheter, analyzes blood gases and sodium, potassium, and hematocrit levels, and returns the sample to the patient. On the command of the operator, the monitor automatically withdraws 1.5-mL blood samples through the UAC, analyzes the samples for blood gases and sodium, potassium, and hematocrit levels, and then reinfuses all except 25 μL of blood. |
| **Outcomes** | *All-cause mortality during initial hospitalization*   - **Outcome type**: Dichotomous Outcome   *Major neurodevelopmental disability*   - **Outcome type**: Continuous Outcome   *Retinopathy of prematurity*   - **Outcome type**: Dichotomous Outcome   *IVH*   - **Outcome type**: Dichotomous Outcome   *Necrotising enterocolitis*   - **Outcome type**: Dichotomous Outcome   *Concentrations of total hemoglobin (Hb)*   - **Outcome type**: Continuous Outcome   *Patent ductus arteriosus (pharmacological treatment and surgical ligation)*   - **Outcome type**: Dichotomous Outcome   *Duration in days of hospital stay*   - **Outcome type**: Continuous Outcome |
| **Identification** | **Sponsorship source:** Not reported.  **Country:** United States of America  **Setting:** NICU at 2 centers  **Comments:**  **Authors name:** John A. Widness  **Institution:** University of Iowa Hospitals and Clinics  **Email:** john-widness@uiowa.edu  **Address:** |
| **Notes** |  |

**Risk of bias table**

| **Bias** | **Authors' judgement** | **Support for judgement** |
| --- | --- | --- |
| Random sequence generation (selection bias) | Low risk | Judgement Comment: Quote: "Subjects were randomized with permuted blocks from a computer-generated random number table." |
| Allocation concealment (selection bias) | Low risk | Judgement Comment: Quote: "After informed consent was obtained, research personnel assigned subjects to study groups by drawing opaque envelopes consecutively." |
| Blinding of participants and personnel (performance bias) | High risk | Judgement Comment: Quote: "The study was conducted as a prospective, randomized, unmasked, 2-center, controlled trial." Quote: "The study was conducted as a prospective, randomized, unmasked, 2-center, controlled trial." |
| Blinding of outcome assessment (detection bias) | High risk | Judgement Comment: The study was conducted as a prospective, randomized, unmasked, 2-center, controlled trial. |
| Incomplete outcome data (attrition bias) | High risk | Judgement Comment: Short-term outcomes: "In conformity with the intention-to-treat approach, data for both surviving and non-surviving infants were included in the primary outcome analysis." For long-term neurodevelopmental outcomes only 23 (of 46) from the intervention group and 25 (of 47) from the control group were used in the analysis. There was no explanation given about these nr. for long-term study data. Short-term outcomes: "In conformity with the intention-to-treat approach, data for both surviving and non-surviving infants were included in the primary outcome analysis." For long-term neurodevelopmental outcomes only 23 (of 46) from the intervention group and 25 (of 47) from the control group were used in the analysis. There was no explanation given about these nr. for long-term study data. |
| Selective reporting (reporting bias) | Unclear risk | Judgement Comment: No protocol is referred to or found. However, the study was published in 2005 when this was not standard practice. Plasma ferritin values not presented for all infants compared to rest of values in same table. (example: plasma ferritin on day 14 only presented for 38 (out of 46 / 42?) infants. The study authors chose not to present all neurodevelopmental outcomes at 18-24 months, simply stating "no difference in the numbers" and "data not shown." Same case with the cumulative number of RBC transfusions administered to the 2 groups, simply stating that "A similar reduction was observed..." presenting only the difference and P-values (0.82 (P = .02) and 0.73 (P = .53) and not the actual data. |
| Other bias | Low risk | Judgement Comment: None |

**References to the 31 included studies:**

### Alan 2014

Alan, S.; Arsan, S.; Okulu, E.; Akin, I. M.; Kilic, A.; Taskin, S.; Cetinkaya, E.; Erdeve, O.; Atasay, B.. Effects of umbilical cord milking on the need for packed red blood cell transfusions and early neonatal hemodynamic adaptation in preterm infants born </=1500 g: a prospective, randomized, controlled trial. Journal of pediatric hematology/oncology 2014;36(8):e493-8. [DOI: 10.1097/mph.0000000000000143]

Alan, S.; Arsan, S.; Okulu, Emel; Akin, I.; Kilic, A.; Taskin, S.; Cetinkaya, Esra; Erdeve, Omer; Atasay, B. Effects of umbilical cord milking on the need for packed red blood cell transfusions and early neonatal hemodynamic adaptation in preterm infants born =1500 g. Archives of disease in childhood. 2014;99:A453‐A454. [DOI: 10.1136/archdischild-2014-307384.1253]

Alan, Serdar; Arsan, Saadet; Okulu, Emel; Akin, Ilke M.; Kilic, Atila; Taskin, Salih; Cetinkaya, Esra; Erdeve, Omer; Atasay, Begum T. I. Effects of umbilical cord milking on the need for packed red blood cell transfusions; early neonatal hemodynamic adaptation in preterm infants, born; amp; g: a prospective, randomized controlled trial. J Pediatr Hematol Oncol 2014;36(8):e493-8. [DOI: ]

### Backes 2016

Backes, C. H.; Huang, H.; Iams, J. D.; Bauer, J. A.; Giannone, P. J.. Timing of umbilical cord clamping among infants born at 22 through 27 weeks' gestation. J Perinatol 2016;36(1):35-40. [DOI: 10.1038/jp.2015.117]

### Baenziger 2007

Baenziger, Oskar; Stolkin, Florian; Keel, Mathias; von Siebenthal, Kurt; Fauchere, Jean-Claude; Das Kundu, Seema; Dietz, Vera; Bucher, Hans-Ulrich; Wolf, Martin. The influence of the timing of cord clamping on postnatal cerebral oxygenation in preterm neonates: a randomized, controlled trial. Pediatrics 2007;119(3):455-9. [DOI: ]

### Balasubramanian 2019

Balasubramanian, H.; Malpani, P.; Sindhur, M.; Kabra, N. S.; Ahmed, J.; Srinivasan, L.. Effect of Umbilical Cord Blood Sampling versus Admission Blood Sampling on Requirement of Blood Transfusion in Extremely Preterm Infants: A Randomized Controlled Trial. Journal of Pediatrics 2019;211:39-45.e2. [DOI: 10.1016/j.jpeds.2019.04.033]

Ctri,. Comparison of the effects of blood sampling from the neonate at birth versus blood sampling from umbilical cord on the need for blood transfusion in very premature infants. http://www.who.int/trialsearch/Trial2.aspx?TrialID=CTRI/2017/04/008320 2017. [DOI: ]

### Chu 2019

Chu, K. S.; Shah, P. S.; Whittle, W. L.; Windrim, R.; Murphy, K. E.. The "DUC" trial: a pilot randomized controlled trial of immediate versus delayed cord clamping in preterm infants born between 24 and 32 weeks gestation. J Matern Fetal Neonatal Med 2019;1-4. [DOI: 10.1080/14767058.2019.1702959]

Windrim, R.; Murphy, K.; Chu, K.; Whittle, W.; Shah, P.. The DUC trial: A pilot randomized controlled trial of immediate vs. delayed umbilical cord clamping in preterm infants born between 24 and 32 weeks gestation. American Journal of Obstetrics and Gynecology 2011;204(1):S201. [DOI: 10.1016/j.ajog.2010.10.521]

### Dipak 2017

Dipak, N. K.; Nanavati, R. N.; Kabra, N. K.; Srinivasan, A.; Ananthan, A.. Effect of delayed cord clamping on hematocrit, and thermal and hemodynamic stability in preterm neonates: A randomized controlled trial. Indian Pediatrics 2017;54(2):112-115. [DOI: 10.1007/s13312-017-1011-8]

### Dong 2016

Dong, Xiao-Yue; Sun, Xiao-Fan; Li, Meng-Meng; Yu, Zhang-Bing; Han, Shu-Ping T. I.. Zhongguo Dang Dai Er Ke Za Zhi 2016;18(7):635-8. [DOI: ]

### Duley 2018

Armstrong-Buisseret, L.; Powers, K.; Dorling, J.; Bradshaw, L.; Johnson, S.; Mitchell, E.; Duley, L.. Randomised trial of cord clamping at very preterm birth: outcomes at 2 years. Arch Dis Child Fetal Neonatal Ed 2019. [DOI: 10.1136/archdischild-2019-316912]

Bradshaw, L. E.; Pushpa-Rajah, A.; Dorling, J.; Mitchell, E. J.; Duley, L.; Bradshaw, L.. Cord pilot trial: Update to randomised trial protocol. Trials 2015;16(1). [DOI: 10.1186/s13063-015-0936-2]

Duley, L.; Abbott, J.; Dorling, J.; Field, D.; Gyte, G.; Oddie, S.; Thornton, J.. Timing of cord clamping and care at the bedside for very preterm birth: A pilot randomised trial. BJOG: An International Journal of Obstetrics and Gynaecology 2013;120:159. [DOI: 10.1111/1471-0528.12293]

Duley, L.; Pushpa-Rajah, A.. Immediate versus deferred cord clamping for very preterm birth: A pilot randomised trial. Archives of Disease in Childhood: Fetal and Neonatal Edition 2014;99:A76-A77. [DOI: 10.1136/archdischild-2014-306576.218]

Duley, L.; Pushpa-Rajah, A.; Dorling, J.. Cord pilot trial: Cord clamping within 20 seconds versus clamping after at least 2 minutes for very preterm births. Archives of Disease in Childhood 2014;99:A457. [DOI: 10.1136/archdischild-2014-307384.1263]

Duley, Lelia; Dorling, Jon; Pushpa-Rajah, Angela; Oddie, Sam J.; Yoxall, Charles William; Schoonakker, Bernard; Bradshaw, Lucy; Mitchell, Eleanor J.; Fawke, Joe Anthony. Randomised trial of cord clamping and initial stabilisation at very preterm birth. Arch Dis Child Fetal Neonatal Ed 2018;103(1):F6-F14. [DOI: ]

Mitchell, E.; Armstrong-Buisseret, L.; Dorling, J.; Bradshaw, L.; Johnson, S.; Powers, K.; Duley, L.. Randomised trial of cord clamping at very preterm birth: Outcomes at two years. BJOG: An International Journal of Obstetrics and Gynaecology 2019;126:14. [DOI: 10.1111/1471-0528.15632]

Pushpa-Rajah, A.; Bradshaw, L.; Dorling, J.; Gyte, G.; Mitchell, E. J.; Thornton, J.; Duley, L.; Murphy, L.; Whitaker, K.; Clarke, M.; Cooke, R.; Kenyon, S.; Steer, P.; Goddard, L.; Branchett, K.; Altman, D.; Devane, D.; Shennan, A.; Stenson, B.; Erven, A.; Field, D.; Oddie, S.; Ezzat, M.; Robertson, A.; Nelson, D.; Young, K.; Schoonakker, B.; Smith, C.; Batra, D.; Aladengady, N.; Kanhari, A.; Van Der Pool, E.; Fawke, J.; Hubbard, M.; Mousa, T.; Yoxall, B.; Peake, M.; Weeks, A.; Churchill, D.; Sutcliffe, M.; Pillay, T.; Kirkwood, G.; Hooton, Y.. Cord pilot trial - immediate versus deferred cord clamping for very preterm birth (before 32 weeks gestation): Study protocol for a randomized controlled trial. Trials 2014;15(1). [DOI: 10.1186/1745-6215-15-258]

### Elimian 2014

Elimian, A.; Goodman, J.; Escobedo, M.; Nightingale, L.; Knudtson, E.; Williams, M.. A randomized controlled trial of immediate versus delayed cord clamping in the preterm neonate. American Journal of Obstetrics and Gynecology 2013;208(1):S22. [DOI: 10.1016/j.ajog.2012.10.209]

Elimian, Andrew; Goodman, Jean; Escobedo, Marilyn; Nightingale, Lydia; Knudtson, Eric; Williams, Marvin. Immediate compared with delayed cord clamping in the preterm neonate: a randomized controlled trial. Obstet Gynecol 2014;124(6):1075-9. [DOI: ]

Nct,. Trial of Immediate vs. Delayed Cord Clamping in the Preterm Neonate. https://clinicaltrials.gov/show/NCT00579839 2007. [DOI: ]

### El Naggar 2019

El-Naggar, W.; McMillan, D.; Hussain, A.; Armson, A.; Dodds, L.; Warren, A.; Whyte, R.; Vincer, M.; Simpson, D.. The effect of umbilical cord milking on neurodevelopmental outcomes of preterm infants at 36 months of age: A randomized controlled trial. Paediatrics and Child Health (Canada) 2019;24:e56. [DOI: 10.1093/pch/pxz066.0141]

El-Naggar, W.; Simpson, D.; Hussain, A.; Armson, A.; Dodds, L.; Warren, A.; Whyte, R.; McMillan, D.. Cord milking versus immediate clamping in preterm infants: a randomised controlled trial. Arch Dis Child Fetal Neonatal Ed 2019;104(2):F145-f150. [DOI: 10.1136/archdischild-2018-314757]

Nct. The Effect of Cord Milking on Hemodynamic Status of Preterm Infants. https://clinicaltrials.gov/show/NCT01487187 2011. [DOI: ]

Walid, El-Naggar; Centre, I. W. K. Health. The Effect of Cord Milking on Hemodynamic Status of Preterm Infants. 2018. [DOI: ]

### Finn 2019

Finn, D.; Ryan, D. H.; Pavel, A.; O'Toole, J. M.; Livingstone, V.; Boylan, G. B.; Kenny, L. C.; Dempsey, E. M.. Clamping the Umbilical Cord in Premature Deliveries (CUPiD): Neuromonitoring in the Immediate Newborn Period in a Randomized, Controlled Trial of Preterm Infants Born at <32 Weeks of Gestation. J Pediatr 2019;208:121-126.e2. [DOI: 10.1016/j.jpeds.2018.12.039]

Isrctn. Clamping the umbilical cord in premature deliveries (CUPID). http://www.who.int/trialsearch/Trial2.aspx?TrialID=ISRCTN92719670 2016. [DOI: ]

### Galderisi 2017

Galderisi, A.; Facchinetti, A.; Steil, G. M.; Ortiz-Rubio, P.; Cobelli, C.; Trevisanuto, D.. Neonatal hypoglycemia continuous glucose monitoring: A randomized controlled trial in preterm infants. Diabetes Technology and Therapeutics 2016;18:A57. [DOI: 10.1089/dia.2016.2525]

Galderisi, A.; Facchinetti, A.; Steil, G.; Ortiz-Rubio, P.; Cavallin, F.; Baraldi, E.; Trevisanuto, D.; Cobelli, C.. Continuous glucose monitoring in very preterm infants: A randomized controlled trial. Diabetes Technology and Therapeutics 2017;19:A34-A35. [DOI: 10.1089/dia.2017.2525.abstracts]

Galderisi, Alfonso; Facchinetti, Andrea; Steil, Garry M.; Ortiz-Rubio, Paulina; Cavallin, Francesco; Tamborlane, William V.; Baraldi, Eugenio; Cobelli, Claudio; Trevisanuto, Daniele. Continuous Glucose Monitoring in Very Preterm Infants: A Randomized Controlled Trial. Pediatrics 2017;140(4). [DOI: ]

Nct. Continuous Glucose Monitoring and Preterm Infants. https://clinicaltrials.gov/show/NCT02583776 2015. [DOI: ]

### Gokmen 2011

Gokmen, Zeynel; Ozkiraz, Servet; Tarcan, Aylin; Kozanoglu, Ilknur; Ozcimen, Emel Ebru; Ozbek, Namik. Effects of delayed umbilical cord clamping on peripheral blood hematopoietic stem cells in premature neonates. J Perinat Med 2011;39(3):323-9. [DOI: ]

### Hosono 2008

Hosono, S.; Mugishima, H.; Fujita, H.; Hosono, A.; Minato, M.; Okada, T.; Takahashi, S.; Harada, K. T. I. Umbilical cord milking reduces the need for red cell transfusions; improves neonatal adaptation in infants born at less than, weeks; gestation: a randomised controlled, trial. Arch Dis Child Fetal Neonatal Ed 2008;93(1):F14-9. [DOI: ]

Hosono, S.; Mugishima, H.; Fujita, H.; Hosono, A.; Okada, T.; Takahashi, S.; Masaoka, N.; Yamamoto, T. T. I. Blood pressure; urine output during the first 120 h of life in infants born at less than, weeks; gestation related to umbilical cord, milking. Arch Dis Child Fetal Neonatal Ed 2009;94(5):F328-31. [DOI: ]

Jprn, Umin. A multicenter randomized control study of the effect of umbilical cord milking in avoiding red cell transfusions in extremely immature infants. http://www.who.int/trialsearch/Trial2.aspx?TrialID=JPRN-UMIN000000990 2008. [DOI: ]

### Josephsen 2014

Josephsen, J.; Vlastos, E.; Potter, S.; Al-Hosni, M.. Milking the umbilical cord in extreme preterm infants. American Journal of Obstetrics and Gynecology 2014;210(1):S403-S404. [DOI: 10.1016/j.ajog.2013.10.863]

St. Louis, University. Milking the Umbilical Cord for Extreme Preterm Infants. 2016. [DOI: ]

### Katheria 2014

Katheria, Anup C.; Leone, Tina A.; Woelkers, Doug; Garey, Donna M.; Rich, Wade; Finer, Neil N.. The effects of umbilical cord milking on hemodynamics and neonatal outcomes in premature neonates. J Pediatr 2014;164(5):1045-1050.e1. [DOI: ]

Katheria, Anup; Blank, Doug; Rich, Wade; Finer, Neil. Umbilical cord milking improves transition in premature infants at birth. PLoS One 2014;9(4):e94085-e94085. [DOI: ]

Nct,. Effects of Milking the Umbilical Cord on Systemic Blood Flow. https://clinicaltrials.gov/show/NCT01434732 2011. [DOI: ]

Sharp, HealthCare. Effects of Milking the Umbilical Cord on Systemic Blood Flow. 2013. [DOI: ]

### Kazemi 2017

Effects of delayed cord clamping on intraventricular hemorrhage in preterm infants. Iranian journal of pediatrics 2017;27(5). [DOI: 10.5812/ijp.6570]

Irct2014091319145N. The Effect Of Delayed Cord Clamping On The Incidence Of Intra Ventricular Hemorrhage. http://www.who.int/trialsearch/Trial2.aspx?TrialID=IRCT2014091319145N1 2014. [DOI: ]

### Kugelman 2007

###### Published and unpublished data

Kugelman A, Borenstein-Levin L, Riskin A, et al.. Immediate Versus Delayed Umbilical Cord Clamping in Premature Neonates Born < 35 Weeks: A Prospective, Randomized, Controlled Study. Am J Perinatol. 2007;24(5):307‐315.

### March 2013

Eastern Virginia Medical, School. Umbilical Cord Milking on the Reduction of Red Blood Cell Transfusion Rates in Infants. 2011. [DOI: ]

March, M. I.; Hacker, M. R.; Parson, A. W.; Modest, A. M.; de Veciana, M.. The effects of umbilical cord milking in extremely preterm infants: a randomized controlled trial. J Perinatol 2013;33(10):763-7. [DOI: ]

March, M.; De Veciana, M.; Parson, A.. The efficacy of umbilical cord milking on the reduction of red blood cell transfusion rates in infants born between 24 and 28 6/7 weeks gestation - A randomized controlled trial. American Journal of Obstetrics and Gynecology 2011;204(1):S204. [DOI: 10.1016/j.ajog.2010.10.531]

Nct. Umbilical Cord Milking on the Reduction of Red Blood Cell Transfusion Rates in Infants. https://clinicaltrials.gov/show/NCT01523769 2011. [DOI: ]

### Mercer 2003

Mercer, Judith S.; McGrath, Margaret M.; Hensman, Angelita; Silver, Helayne; Oh, William. Immediate and delayed cord clamping in infants born between 24 and 32 weeks: a pilot randomized controlled trial. J Perinatol 2003;23(6):466-72. [DOI: ]

### Mercer 2006

Mercer, J. S.; Vohr, B. R.; Erickson-Owens, D. A.; Padbury, J. F.; Oh, W.. Seven-Month Neurodevelopmental Outcomes of Infants Enrolled in a Randomized Controlled Trial of Delayed Versus Immediate Cord Clamping. Pediatric academic societies annual meeting; 2009 may 2 5; baltimore MD, united states 2009. [DOI: ]

Mercer, J. S.; Vohr, B. R.; Erickson-Owens, D. A.; Padbury, J. F.; Oh, W.. Seven-month developmental outcomes of very low birth weight infants enrolled in a randomized controlled trial of delayed versus immediate cord clamping. J Perinatol 2010;30(1):11-6. [DOI: ]

Mercer, Judith S.; Vohr, Betty R.; McGrath, Margaret M.; Padbury, James F.; Wallach, Michael; Oh, William. Delayed cord clamping in very preterm infants reduces the incidence of intraventricular hemorrhage and late-onset sepsis: a randomized, controlled trial. Pediatrics 2006;117(4):1235-42. [DOI: ]

Sommers, R.; Stonestreet, B. S.; Oh, W.; Laptook, A.; Yanowitz, T. D.; Raker, C.. Hemodynamic effects of delayed cord clamping in premature infants. Pediatrics 2012;129(3):e667 // 72. [DOI: 10.1542/peds.2011-2550]

### Mercer 2016

Delayed Cord Clamping in Infants with Suspected Intrauterine Growth Restriction. Journal of pediatrics 2018. [DOI: 10.1016/j.jpeds.2018.05.028]

Mercer, J. S.; Erikson-Owens, D. A.; Vohr, B. R.; Tucker, R.; Oh, W.; Padbury, J. F.. Delayed cord clamping at birth improves motor scores at 18 to 22 months corrected age: a randomized controlled trial. Pediatric academic societies (PAS) annual meeting; 2015 apr 25 - 28; san diego, USA 2015. [DOI: ]

Mercer, Judith S.; Erickson-Owens, Debra A.; Vohr, Betty R.; Tucker, Richard J.; Parker, Ashley B.; Oh, William; Padbury, James F.. Effects of Placental Transfusion on Neonatal and 18 Month Outcomes in Preterm Infants: A Randomized Controlled Trial. J Pediatr 2016;168:50-5.e1. [DOI: ]

Nct. 18 Month Follow Up of Preterm Infants Enrolled in the Cord Clamping Study. https://clinicaltrials.gov/show/NCT01426698 2011. [DOI: ]

Nct. Effects of Delayed Cord Clamping in Very Low Birth Weight Infants. https://clinicaltrials.gov/show/NCT00840983 2009. [DOI: ]

### Nelle 2012

Nelle, M.; Walter, K. N.; Gerull, R.. Late cord-clamping improves circulation in neonates. Archives of Disease in Childhood 2012;97:A6. [DOI: 10.1136/archdischild-2012-302724.0019]

### Oh 2011

Network, Nichd Neonatal Research; National Center for Research, Resources. Delayed Cord Clamping in VLBW Infants. 2000. [DOI: ]

Oh, W.; Carlo, W. A.; Fanaroff, A. A.; McDonald, S.; Donovan, E. F.; Poole, K.. Delayed cord clamping in extremely low birthweight infants - a pilot randomized controlled trial. Pediatric research 2002;5(4 Suppl):365‐366. [DOI: ]

Oh, W.; Fanaroff, A. A.; Carlo, W. A.; Donovan, E. F.; McDonald, S. A.; Poole, W. K.. Effects of delayed cord clamping in very-low-birth-weight infants. J Perinatol 2011;31 Suppl 1:S68-71. [DOI: ]

### Prescott 2014

NCT02103296; Capt Alicia Prescott, Yes; C. R.Darnall Army Medical Center; Walter Reed National Military Medical, Center; United States Naval Medical Center, Portsmouth; Madigan Army Medical, Center; Tripler Army Medical, Center. Umbilical Cord Blood Use For Admission Blood Tests of Very Low Birth Weight Preterm Neonates: A Multi-center Randomized Clinical Trial. 2014. [DOI: ]

### Rabe 2000

Rabe, H.; Hentschel, R.; Brune, T.; Hulskamp, G.; Jorch, G.. A randomised study of delayed cord clamping: the starting point in treatment of anamia of prematurity. Prenatal and neonatal medicine 1996;1 Suppl 1:174. [DOI: ]

Rabe, H.; Wacker, A.; Hulskamp, G.; Hornig-Franz, I.; Schulze-Everding, A.; Harms, E.; Cirkel, U.; Louwen, F.; Witteler, R.; Schneider, H. P.. A randomised controlled trial of delayed cord clamping in very low birth weight preterm infants. European journal of pediatrics 2000;159(10):775-7. [DOI: 10.1007/pl00008345]

### Silahli 2018

Silahli, M.; Duman, E.; Gokmen, Z.; Toprak, E.; Gokdemir, M.; Ecevit, A.. The relationship between placental transfusion, and thymic size and neonatal morbidities in premature infants - A Randomized Control Trial. J Pak Med Assoc 2018;68(11):1560-1565. [DOI: ]

### Tarnow Mordi 2018

Actrn. Australian Placental Transfusion Pilot Study: investigating standard cord clamping procedures versus three methods of autologous placental blood transfusion in pre term infants. http://www.who.int/trialsearch/Trial2.aspx?TrialID=ACTRN12609000248268 2009. [DOI: ]

Nct. The Australian Placental Transfusion Study (APTS): should Very Pre Term Babies Receive a Placental Blood Transfusion at Birth Via Deferring Cord Clamping Versus Standard Cord Clamping Procedures? https://clinicaltrials.gov/show/NCT02606058 2015. [DOI: ]

Popat, H.; Galea, C.; Evans, N.; Lingwood, B.; Colditz, P.; Halliday, R.; Greenhalgh, M.; Malcolm, G.; Osborn, D.. Effect of delayed cord clamping on cerebral oxygenation in preterm infants <30 weeks gestation. Journal of Paediatrics and Child Health 2017;53:80. [DOI: 10.1111/jpc.13494_237]

Popat, H.; Mann, K.; Buchan, J.; Brown, R.; Cornthwaite, K.; De Waal, K.; Evans, N.; Gill, A.; Hague, W.; Hecker, T.; et al.. Australian placental transfusion study echo sub-study: effect on systemic blood flow. Journal of paediatrics and child health 2015;51:17‐. [DOI: 10.1111/jpc.12884]

Popat, Himanshu; Robledo, Kristy P.; Sebastian, Lucille; Evans, Nicholas; Gill, Andrew; Kluckow, Martin; Sinhal, Sanjay; Waal, Koert de; Tarnow-Mordi, William; Osborn, David. Interobserver agreement and image quality of functional cardiac ultrasound measures used in a randomised trial of delayed cord clamping in preterm infants. Arch Dis Child Fetal Neonatal Ed 2018;103(3):F257-F263. [DOI: ]

Popat, Himanshu; Robledo, Kristy P.; Sebastian, Lucille; Evans, Nicholas; Gill, Andrew; Kluckow, Martin; Sinhal, Sanjay; de Waal, Koert; Tarnow-Mordi, William; Osborn, David. Effect of Delayed Cord Clamping on Systemic Blood Flow: A Randomized Controlled Trial. J Pediatr 2016;178:81-86.e2. [DOI: ]

Tarnow-Mordi, W.; Morris, J.; Kirby, A.; Robledo, K.; Askie, L.; Brown, R.; Evans, N.; Finlayson, S.; Fogarty, M.; Gebski, V.; Ghadge, A.; Hague, W.; Isaacs, D.; Jeffery, M.; Keech, A.; Kluckow, M.; Popat, H.; Sebastian, L.; Aagaard, K.; Belfort, M.; Pammi, M.; Abdel-Latif, M.; Reynolds, G.; Ariff, S.; Sheikh, L.; Chen, Y.; Colditz, P.; Liley, H.; Pritchard, M.; De Luca, D.; De Waal, K.; Forder, P.; Duley, L.; El-Naggar, W.; Gill, A.; Newnham, J.; Simmer, K.; Groom, K.; Weston, P.; Gullam, J.; Patel, H.; Koh, G.; Lui, K.; Marlow, N.; Morris, S.; Sehgal, A.; Wallace, E.; Soll, R.; Young, L.; Sweet, D.; Walker, S.; Watkins, A.; Wright, I.; Osborn, D.; Simes, J.. Delayed Versus Immediate Cord Clamping in Preterm Infants. Obstetrical and Gynecological Survey 2018;73(5):265-266. [DOI: 10.1097/01.ogx.0000534708.24689.e0]

Tarnow-Mordi, W.; Morris, J.; Kirby, A.; Robledo, K.; Askie, L.; Brown, R.; Evans, N.; Finlayson, S.; Fogarty, M.; Gebski, V.; et al.. Delayed versus Immediate Cord Clamping in Preterm Infants. New England journal of medicine 2017;377(25):2445‐2455. [DOI: 10.1056/NEJMoa1711281]

### Thomson 2018

Thomson, L.; Elleri, D.; Bond, S.; Howlett, J.; Dunger, D. B.; Beardsall, K.. Targeting glucose control in preterm infants: pilot studies of continuous glucose monitoring. Archives of disease in childhood: fetal and neonatal edition 2018. [DOI: 10.1136/archdischild-2018-314814]

### Uettwiller 2015

Nct. Real-time Continuous Glucose Monitoring in Very Low Birth Weight Neonates. https://clinicaltrials.gov/show/NCT01942239 2013. [DOI: ]

Uettwiller, Florence; Chemin, Aude; Bonnemaison, Elisabeth; Favrais, Géraldine; Saliba, Elie; Labarthe, François. Real-time continuous glucose monitoring reduces the duration of hypoglycemia episodes: a randomized trial in very low birth weight neonates. PLoS One 2015;10(1):e0116255-e0116255. [DOI: ]

### Widness 2005

Widness, John A.; Madan, Ashima; Grindeanu, Ligia A.; Zimmerman, M. Bridget; Wong, David K.; Stevenson, David K.. Reduction in red blood cell transfusions among preterm infants: results of a randomized trial with an in-line blood gas and chemistry monitor. Pediatrics 2005;115(5):1299-306. [DOI: ]

Top of Form

Bottom of Form
