# Supplementary material for: Interventions to minimize blood loss in very preterm infants—A systematic review and meta-analysis
Source: PLoS One. 2021 Feb 8;16(2):e0246353. doi: 10.1371/journal.pone.0246353 (PMC7870155; doi:10.1371/journal.pone.0246353)
Supplement: S3 File — (DOCX) [file pone.0246353.s004.docx]

**Excluded studies**

In total 80 studies where excluded:

Infants > 32 weeks: 21 studies

Wrong comparator (e.g. no immediate cord clamping): 21 studies

Wrong study design (e.g. not an RCT): 15 studies

Duplicate study: 11 studies

Wrong intervention (e.g. delayed cord clamping done before 30 sec): 7 studies

Terminated: 3 studies

Not enough information: 2 studies

| **Reason for exclusion:** | **Excluded studies:** |
| --- | --- |
| Infants > 32 weeks | CerianiCernadas 2010  CerianiCernadas 2012  Chaparro 2006  Kc 2017  Kc 2019  Kilbride 1984  Krowchuk 2017  Kugelman 2009  Kumar 2015  LagoLeal 2018  LagoLeal 2019  Nct 2012  Nct 2016a  Nct 2018d  Nct 2018e  Nct 2018f  Nct 2018g  NCT03621943 2018  Ranjit 2015  Ruangkit 2018  Sharp 2022a |
| Wrong comparator (e.g. no immediate cord clamping) | Ankara 2010  Ibrahim 2000  Isrctn 2017  K 2017  Katheria 2015  Katheria 2019  KingAbdulaziz 2019  Nct 2014a  Nct 2015  Nct 2016  Nct 2019  Pratesi 2018  Rabe 2009  Rabe 2010  Rabe 2016  Sharp 2014  Sharp 2022  Tctr 2015  TriHealth 2018  Universityof 2017a  WeillMedicalCollegeofCornell 2019 |
| Wrong study design (e.g. not an RCT) | Carroll 2015  Cernadas 2015  Daz Rossello 2006  FernndezMedina 2014  FondazionePoliclinicoUniversitarioAgostinoGemelli 2019  Galderisi 2018  Jprn 2017  JPRN UMIN000026456 2017  Kaempf 2012  Kamper 1983  Katheria 2018  Kinsley 2018  Popat 2019  Tobias 2019  Universityof 2019 |
| Duplicate study | Aladangady 2006a  Dong 2016a  Elimian 2014a  Hosono 2009  HospitalUniversitariValldHebronResearch 2017  IRCT20100512003915N22 2019  JubileeMissionMedical 2019  Li 2018  Nct 2017a  Rabe 2010a  Universityof 2017 |
| Wrong intervention (e.g. delayed cord clamping done before 30 sec) | Centre 2021  Irct20141208020249N 2018  Katheria 2016  Katheria 2019a  Kugelman 2016  Nct 2011  Nct 2019a |
| Terminated | JohnsHopkins 2013  Nct 2013  UniversityofCalifornia 2018 |
| Not enough information | Isrctn 2006  Phillips 2011 |

References:

**Aladangady 2006a**

Aladangady, N.; McHugh, S.; Aitchison, T. C.; Wardrop, C. A.; Holland, B. M.. Infants' blood volume in a controlled trial of placental transfusion at preterm delivery. Pediatrics 2006;117(1):93-8. [DOI: 10.1542/peds.2004-1773]

**Ankara 2010**

Ankara, University. Autologous Cord Blood Transfusion in Preterm Infants. 2010. [DOI: ]

**Carroll 2015**

Carroll, P. D.. Umbilical Cord Blood-An Untapped Resource: Strategies to Decrease Early Red Blood Cell Transfusions and Improve Neonatal Outcomes. Clinics in Perinatology 2015;42(3):541-556. [DOI: 10.1016/j.clp.2015.04.01

**Centre 2021**

Centre, I. W. K. Health. Milking of the Cut-Cord During Resuscitation of Preterm Infants (The MOCC Study). 2021. [DOI: ]

**CerianiCernadas 2010**

Ceriani Cernadas, J. M.; Carroli, G.; Pellegrini, L.; Ferreira, M.; Ricci, C.; Casas, O.; Lardizabal, J.; Morasso, M. D. C.. The effect of early and delayed umbilical cord clamping on ferritin levels in term infants at six months of life. A randomized, controlled trial. Archivos Argentinos de Pediatria 2010;108(3):201-208. [DOI: ]

**CerianiCernadas 2012**

Ceriani Cernadas, J. M.; Carroli, G.; Pellegrini, L.; Ferreira, M.; Ricci, C.; Casas, O.; Lardizabal, J.; Morasso, M. C.. The effect of early and delayed umbilical cord clamping on ferritin levels in term infants at six months of life. A randomized, controlled trial. Revista Chilena de Pediatria 2012;83(1):86-87. [DOI: 10.4067/S0370-41062012000100012]

**Cernadas 2015**

Cernadas, J. M. C.. Effect of delayed cord clamping on neurodevelopment at 4 years of age. A randomized clinical trial. Archivos Argentinos de Pediatria 2015;113(5):479-480. [DOI: ]

**Chaparro 2006**

Chaparro, C. M.; Neufeld, L. M.; Tena Alavez, G.; Eguia-Liz Cedillo, R.; Dewey, K. G.. Effect of timing of umbilical cord clamping on iron status in Mexican infants: a randomised controlled trial. Lancet (London, England) 2006;367(9527):1997-2004. [DOI: 10.1016/s0140-6736(06)68889-2]

**Daz Rossello 2006**

Díaz-Rossello, J. L.. Early umbilical cord clamping and cord-blood banking...Chaparro CM, Neufeld LM, Tena Alavez G et al. Effect of timing of umbilical cord clamping on iron status in Mexican infants: a randomized controlled trial. Lancet 2006;367:1997-2004. Lancet 2006;368 North American Edition(9538):840-840. [DOI: ]

**Dong 2016a**

Dong, X. Y.; Sun, X. F.; Li, M. M.; Yu, Z. B.; Han, S. P.. [Influence of delayed cord clamping on preterm infants with a gestational age of <32 weeks]. Zhongguo Dang Dai Er Ke Za Zhi 2016;18(7):635-8. [DOI: ]

**Elimian 2014a**

Elimian, A.; Goodman, J.; Escobedo, M.; Nightingale, L.; Knudtson, E.; Williams, M.. Immediate compared with delayed cord clamping in the preterm neonate. Obstetrics and gynecology 2014;124(6):1075‐1079. [DOI: 10.1097/AOG.0000000000000556]

**FernndezMedina 2014**

Fernández Medina, Isabel María. Late clamping of the umbilical cord in premature neonates: The real haemodynamic benefits. Enfermeria Clinica 2014;24(5):305-307. [DOI: 10.1016/j.enfcli.2013.10.005]

**FondazionePoliclinicoUniversitarioAgostinoGemelli 2019**

Fondazione Policlinico Universitario Agostino Gemelli, Irccs. Cord Blood Transfusion In Preterm Neonates (CB-TrIP). 2019. [DOI: ]

**Galderisi 2018**

Galderisi, Alfonso; Lago, Paola; Steil, Garry M.; Ghirardo, Martina; Cobelli, Claudio; Baraldi, Eugenio; Trevisanuto, Daniele. Procedural Pain during Insertion of a Continuous Glucose Monitoring Device in Preterm Infants. Journal of Pediatrics 2018;200:261-264.e1. [DOI: 10.1016/j.jpeds.2018.03.040]

**Hosono 2009**

Hosono, S.; Mugishima, H.; Fujita, H.; Hosono, A.; Okada, T.; Takahashi, S.; Masaoka, N.; Yamamoto, T.. Blood pressure and urine output during the first 120 h of life in infants born at less than 29 weeks' gestation related to umbilical cord milking. Arch Dis Child Fetal Neonatal Ed 2009;94(5):F328-31. [DOI: 10.1136/adc.2008.142935]

**HospitalUniversitariValldHebronResearch 2017**

Hospital Universitari Vall d'Hebron Research, Institute. Timing of Umbilical Cord Occlusion in Premature Babies( <33 w). Delayed vs Early. 2017. [DOI: ]

**Ibrahim 2000**

Ibrahim, H. M.; Krouskop, R. W.; Lewis, D. F.; Dhanireddy, R.. Placental transfusion: umbilical cord clamping and preterm infants. J Perinatol 2000;20(6):351-4. [DOI: ]

**IRCT20100512003915N22 2019**

IRCT20100512003915N22; Tabriz University of Medical Sciences, No. the short term outcome of three different umbilical cord clamping methods. 2019. [DOI: ]

**Irct20141208020249N 2018**

Irct20141208020249N. early outcome effects in delayed umbilical cord clamping. http://www.who.int/trialsearch/Trial2.aspx?TrialID=IRCT20141208020249N2 2018. [DOI: ]

**Isrctn 2006**

Isrctn. Effects of a slight delay in cord clamping time versus milking the cord in preterm infants. http://www.who.int/trialsearch/Trial2.aspx?TrialID=ISRCTN86296143 2006. [DOI: ]

**Isrctn 2017**

Isrctn. Early compared to delayed umbilical cord clamping in very small prematurely born babies: a study to know which one is better for infant health. http://www.who.int/trialsearch/Trial2.aspx?TrialID=ISRCTN12219110 2017. [DOI: ]

**JohnsHopkins 2013**

Johns Hopkins, University. Milking the Umbilical Cord Versus Immediate Clamping in Pre-term Infants < 33 Weeks. 2013. [DOI: ]

**Jprn 2017**

Jprn, Umin. Effects of delayed cord clamping in preterm infants. http://www.who.int/trialsearch/Trial2.aspx?TrialID=JPRN-UMIN000026456 2017. [DOI: ]

**JPRN UMIN000026456 2017**

JPRN-UMIN000026456; Niigata University, Medical; amp,; Dental Hospital, No. Effects of delayed cord clamping in preterm infants. 2017. [DOI: ]

**JubileeMissionMedical 2019**

Jubilee Mission Medical, College; Research, Institute. Effect of Intact Umbilical Cord Milking on Neonatal and First Year Neurodevelopmental Outcomes in Very Preterm Infants. 2019. [DOI: ]

**K 2017**

K. C, Ashish; Rana, Nisha; Målqvist, Mats; Ranneberg, Linda Jarawka; Subedi, Kalpana; Andersson, Ola. Effects of Delayed Umbilical Cord Clamping vs Early Clamping on Anemia in Infants at 8 and 12 Months. JAMA Pediatrics 2017;171(3):264-270. [DOI: 10.1001/jamapediatrics.2016.3971]

**Kaempf 2012**

Kaempf, J. W.; Tomlinson, M. W.; Kaempf, A. J.; Wu, Y.; Wang, L.; Tipping, N.; Grunkemeier, G.. Delayed umbilical cord clamping in premature neonates. Obstetrics and gynecology 2012;120(2 Pt 1):325-30. [DOI: 10.1097/AOG.0b013e31825f269f]

**Kamper 1983**

Kamper, J.; Nielsen, G.; Erichsen, G.; Filtenborg, J. A.; Lillquist, K.; Pedersen, V. F.; Skjolda, J.; Stabell, I.. Transcutaneous PO2 monitoring during treatment with continuous positive airway pressure in infants with idiopathic respiratory distress syndrome. Acta anaesthesiologica Scandinavica 1983;27(1):1-4. [DOI: 10.1111/j.1399-6576.1983.tb01894.x]

**Katheria 2015**

Katheria, Anup C.; Giang, Truong; Cousins, Larry; Oshiro, Bryan; Finer, Neil N.. Umbilical Cord Milking Versus Delayed Cord Clamping in Preterm Infants. Pediatrics 2015;136(1):61-69. [DOI: 10.1542/peds.2015-0368]

**Katheria 2016**

Katheria, Anup; Poeltler, Debra; Durham, Jayson; Steen, Jane; Rich, Wade; Arnell, Kathy; Maldonado, Mauricio; Cousins, Larry; Finer, Neil. Neonatal Resuscitation with an Intact Cord: A Randomized Clinical Trial. J Pediatr 2016;178:75-80.e3. [DOI: ]

**Katheria 2018**

Katheria, A. C.. Delayed cord clamping may not be beneficial in the premature infant. Journal of Pediatrics 2018;196:324-327. [DOI: 10.1016/j.jpeds.2018.02.057]

**Katheria 2019**

Katheria, A. C.; Reister, F.; Hummler, H.; Essers, J.; Mendler, M.; Truong, G.; Davis-Nelson, S.; Subramaniam, A.; Carlo, W.; Yankowitz, T. D.; et al.. LB 1: premature Infants Receiving Cord Milking or Delayed Cord Clamping: a Randomized Controlled Non-inferiority Trial. American journal of obstetrics and gynecology 2019;220(1):S682‐. [DOI: 10.1016/j.ajog.2018.12.004]

**Katheria 2019a**

Katheria, A.; Reister, F.; Essers, J.; Mendler, M.; Hummler, H.; Subramaniam, A.; Carlo, W.; Tita, A.; Truong, G.; Davis-Nelson, S.; Schmolzer, G.; Chari, R.; Kaempf, J.; Tomlinson, M.; Yanowitz, T.; Beck, S.; Simhan, H.; Dempsey, E.; O'Donoghue, K.; Bhat, S.; Hoffman, M.; Faksh, A.; Arnell, K.; Rich, W.; Finer, N.; Vaucher, Y.; Khanna, P.; Meyers, M.; Varner, M.; Allman, P.; Szychowski, J.; Cutter, G.. Association of Umbilical Cord Milking vs Delayed Umbilical Cord Clamping With Death or Severe Intraventricular Hemorrhage Among Preterm Infants. Jama 2019;322(19):1877-1886. [DOI: 10.1001/jama.2019.16004]

**Kc 2017**

Kc, A.; Rana, N.; Malqvist, M.; Jarawka Ranneberg, L.; Subedi, K.; Andersson, O.. Effects of Delayed Umbilical Cord Clamping vs Early Clamping on Anemia in Infants at 8 and 12 Months: A Randomized Clinical Trial. JAMA pediatrics 2017;171(3):264-270. [DOI: 10.1001/jamapediatrics.2016.3971]

**Kc 2019**

Kc, A.; Singhal, N.; Gautam, J.; Rana, N.; Andersson, O.. Effect of early versus delayed cord clamping in neonate on heart rate, breathing and oxygen saturation during first 10 minutes of birth - randomized clinical trial. Maternal health, neonatology and perinatology 2019;5:7. [DOI: 10.1186/s40748-019-0103-y]

**Kilbride 1984**

Kilbride, H. W.; Merenstein, G. B.. Continuous transcutaneous oxygen monitoring in acutely ill preterm infants. Critical care medicine 1984;12(2):121-4. [DOI: 10.1097/00003246-198402000-00009]

**KingAbdulaziz 2019**

King Abdulaziz, University. Deferred Cord Clamping Compared to Umbilical Cord Milking in Preterm Infants. 2019. [DOI: ]

**Kinsley 2018**

Kinsley, T.. Beneficial effects of delayed cord clamping on the respiratory system in premature infants. Canadian Journal of Respiratory Therapy 2018;54(2):52. [DOI: ]

**Krowchuk 2017**

Krowchuk, Heidi V.. Effects of Delayed Cord Clamping on Residual Placental Blood Volume, Hemoglobin and Bilirubin Levels in Term infants: A Randomized Controlled Trial. MCN: The American Journal of Maternal Child Nursing 2017;42(5):300-300. [DOI: 10.1097/NMC.0000000000000360]

**Kugelman 2009**

Kugelman, Amir; Borenstein-Levin, Liron; Kessel, Aharon; Riskin, Arieh; Toubi, Elias; Bader, David. Immunologic and infectious consequences of immediate versus delayed umbilical cord clamping in premature infants: a prospective, randomized, controlled study. J Perinat Med 2009;37(3):281-7. [DOI: ]

**Kugelman 2016**

Kugelman, A.; Golan, A.; Riskin, A.; Shoris, I.; Ronen, M.; Qumqam, N.; Bader, D.; Bromiker, R.. Impact of Continuous Capnography in Ventilated Neonates: A Randomized, Multicenter Study. The Journal of pediatrics 2016;168:56-61.e2. [DOI: 10.1016/j.jpeds.2015.09.051]

**Kumar 2015**

Kumar, Bimlesh; Upadhyay, Amit; Gothwal, Sunil; Jaiswal, Vijay; Joshi, Payas; Dubey, Kirti. Umbilical Cord Milking and Hematological Parameters in Moderate to Late Preterm Neonates: A Randomized Controlled Trial. Indian Pediatr 2015;52(9):753-7. [DOI: ]

**LagoLeal 2018**

Lago Leal, V.; Pamplona Bueno, L.; Cabanillas Vilaplana, L.; Nicolas Montero, E.; Martin Blanco, M.; Fernandez Romero, C.; El Bakkali, S.; Pradillo Aramendi, T.; Sobrino Lorenzano, L.; Castellano Esparza, P.; et al.. Effect of Milking Maneuver in Preterm Infants: a Randomized Controlled Trial. Fetal diagnosis and therapy 2018. [DOI: 10.1159/000485654]

**LagoLeal 2019**

Lago Leal, V.; Nicolás Montero, E.; Martín Blanco, M.; Fernández Romero, C.; El Bakkali, S.; Pradillo Aramendi, T.; Sobrino Lorenzano, L.; Castellano Esparza, P.; Ballesteros Benito, E.; Rayo Navarro, N.; Del Barrio Fernández, P.; Ocaña Martínez, V.; Martínez Cortés, L.. Effect of Milking Maneuver in Preterm Infants: A Randomized Controlled Trial. Fetal Diagnosis and Therapy 2019;45(1):57-61. [DOI: 10.1159/000485654]

**Li 2018**

Li, J.; Yu, B.; Wang, W.; Luo, D.; Dai, Q. L.; Gan, X. Q.. Does intact umbilical cord milking increase infection rates in preterm infants with premature prolonged rupture of membranes? Journal of Maternal-Fetal & Neonatal Medicine 2018. [DOI: 10.1080/14767058.2018.1487947]

**NCT03621943 2018**

NCT03621943; Sharp HealthCare, Yes. Umbilical Cord Milking in Non-Vigorous Infants Developmental Followup (MINVIFU). 2018. [DOI: ]

**Nct 2011**

Nct. Effect of Delayed Cord Clamping on Haematological Status in Low Birth Weight Infants. https://clinicaltrials.gov/show/NCT01487980 2011. [DOI: ]

**Nct 2012**

Nct. Delayed Cord Clamping and Infant Brain Study. https://clinicaltrials.gov/show/NCT01620008 2012. [DOI: ]

**Nct 2013**

Nct. Milking the Umbilical Cord Versus Immediate Clamping in Pre-term Infants < 33 Weeks. https://clinicaltrials.gov/show/NCT01819532 2013. [DOI: ]

**Nct 2014a**

Nct. Delayed Cord Clamping in Very Low Birth Weight Infants. https://clinicaltrials.gov/show/NCT02337088 2014. [DOI: ]

**Nct 2015**

Nct. Delayed Cord Clamping in Preterm Neonates. https://clinicaltrials.gov/show/NCT02478684 2015. [DOI: ]

**Nct 2016**

Nct. VentFirst: a Multicenter RCT of Assisted Ventilation During Delayed Cord Clamping for Extremely Preterm Infants. https://clinicaltrials.gov/show/NCT02742454 2016. [DOI: ]

**Nct 2016a**

Nct. Early or Late Cord Clamping in the Depressed Neonate. https://clinicaltrials.gov/show/NCT02727517 2016. [DOI: ]

**Nct 2017a**

Nct,. Effect of Intact Umbilical Cord Milking on Neonatal and First Year Neurodevelopmental Outcomes in Very Preterm Infants. https://clinicaltrials.gov/show/NCT03200301 2017. [DOI: ]

**Nct 2018d**

Nct. Umbilical Cord Milking in Neonates Who Are Depressed at Birth-Developmental Follow Up (MIDAB-FU). https://clinicaltrials.gov/show/NCT03681314 2018. [DOI: ]

**Nct 2018e**

Nct. Umbilical Cord Milking in Non-Vigorous Infants Developmental Followup (MINVIFU). https://clinicaltrials.gov/show/NCT03621943 2018. [DOI: ]

**Nct 2018f**

Nct. Influence of Umbilical Cord Clamping Time in the Newborn. https://clinicaltrials.gov/show/NCT03624335 2018. [DOI: ]

**Nct 2018g**

Nct. Umbilical Cord Milking in Non-Vigorous Infants. https://clinicaltrials.gov/show/NCT03631940 2018. [DOI: ]

**Nct 2019**

Nct. The Effect of Delayed Cord Clamping and Milking on the Amount of Stem Cells In Preterm. https://clinicaltrials.gov/show/NCT04057027 2019. [DOI: ]

**Nct 2019a**

Nct. Aeration, Breathing, Clamping Study 3. https://clinicaltrials.gov/show/NCT03808051 2019. [DOI: ]

**Phillips 2011**

Phillips, Charles; Clifton-Koeppel, Robin; Sills, Jack; Lomax, Jacqueline M.; Rapini, Molly; Huffman, Matt L.; Modanlou, Houchang D.. Capillary blood draws in the nicu: the use of the innovac quick-draw whole blood collection system versus traditional capillary blood draws. Neonatal Network 2011;30(3):175-178. [DOI: 10.1891/0730-0832.30.3.175]

**Popat 2019**

Popat, H.; Robledo, K. P.; Kirby, A.; Sebastian, L.; Evans, N.; Gill, A.; Kluckow, M.; Sinhal, S.; de Waal, K.; Tarnow-Mordi, W.; et al.,. Associations of measures of systemic blood flow used in a randomized trial of delayed cord clamping in preterm infants. Pediatric Research 2019. [DOI: 10.1038/s41390-019-0348-1]

**Pratesi 2018**

Pratesi, S.; Montano, S.; Ghirardello, S.; Mosca, F.; Boni, L.; Tofani, L.; Dani, C.. Placental Circulation Intact Trial (PCI-T)-Resuscitation With the Placental Circulation Intact vs. Cord Milking for Very Preterm Infants: A Feasibility Study. Frontiers in pediatrics 2018;6:364. [DOI: 10.3389/fped.2018.00364]

**Rabe 2009**

Rabe, H.; Holden, D.; Bradley, R.; Jewison, A.; Fernandez Alvarez, J. R.; Stilton, D.; Hogarth, S.. Randomized trial of slightly delayed cord clamping (DCC) versus milking of the cord (MC) in preterm infants. 50th annual meeting of the european society for paediatric research; 2009 october 9-12; hamburg, germany 2009. [DOI: ]

**Rabe 2010**

Rabe, H.; Jewison, A.; Alvarez, R. F.; Stilton, D.; Bradley, R.; Holden, D.. Randomized controlled trial (RCT) on 4 times milking of the cord versus slight delay of cord clamping in very low birth weight infants (VLBW): effects on circulation. Pediatric academic societies annual meeting; 2010 may 1-4; vancouver, canada 2010. [DOI: ]

**Rabe 2010a**

Rabe. Randomized Controlled Trial (RCT) on 4 Times Milking of the Cord Versus Slight Delay of Cord Clamping in Very Low Birth Weight Infants (VLBW): effects on Circulaton. Pediatric academic society 2010;http://www.abstracts2view.com/pas/(1471.204). [DOI: ]

**Rabe 2016**

Rabe, Heike; Sawyer, Alexandra; Amess, Philip; Ayers, Susan. Neurodevelopmental Outcomes at 2 and 3.5 Years for Very Preterm Babies Enrolled in a Randomized Trial of Milking the Umbilical Cord versus Delayed Cord Clamping. Neonatology 2016;109(2):113-9. [DOI: ]

**Ranjit 2015**

Ranjit, Thomas; Nesargi, Saudamini; Rao, P. N. Suman; Sahoo, Jagdish Prasad; Ashok, C.; Chandrakala, B. S.; Bhat, Swarnarekha. Effect of early versus delayed cord clamping on hematological status of preterm infants at 6 wk of age. Indian J Pediatr 2015;82(1):29-34. [DOI: ]

**Ruangkit 2018**

Ruangkit, C.; Bumrungphuet, S.; Panburana, P.; Khositseth, A.; Nuntnarumit, P.. A Randomized Controlled Trial of Immediate versus Delayed Umbilical Cord Clamping in Multiple-Birth Infants Born Preterm. Neonatology 2018;156‐163. [DOI: 10.1159/000494132]

**Sharp 2014**

Sharp, HealthCare; Eunice Kennedy Shriver National Institute of Child, Health; Human, Development. The PREMOD Trial: A Randomized Controlled Trial of Umbilical Cord Milking vs. Delayed Cord Clamping in Premature Infants. 2014. [DOI: ]

**Sharp 2022**

Sharp, HealthCare; Sharp Mary Birch Hospital for, Women; Newborns; Loma Linda, University; University of, Pittsburgh; Providence, Hospital; University of Alabama at, Birmingham; University of, Alberta; University College, Cork; University of, Ulm; Eunice Kennedy Shriver National Institute of Child, Health; Human, Development; Christiana Care Health, Services; Sharp Grossmont, Hospital; University of, Utah. Premature Infants Receiving Milking or Delayed Cord Clamping: PREMOD2. 2022. [DOI: ]

**Sharp 2022a**

Sharp, HealthCare; Sharp Mary Birch Hospital for, Women; Newborns; Loma Linda, University; University of, Pittsburgh; Providence, Hospital; University of Alabama at, Birmingham; University of, Alberta; University College, Cork; University of, Ulm; Christiana Care Health, Services; Thrasher Research, Fund; Sharp Grossmont, Hospital; University of, Utah. Two Year Developmental Follow-up for PREMOD2 Trial (Premature Infants Receiving Milking or Delayed Cord Clamping). 2022. [DOI: ]

**Tctr 2015**

Tctr. Effect of delayed cord clamping versus cord milking in infants born at < 34 weeks? gestation: a randomized controlled trial. http://www.who.int/trialsearch/Trial2.aspx?TrialID=TCTR20150106001 2015. [DOI: ]

**Tobias 2019**

Tobias, Werther; Medical University of, Vienna. Non-Invasive Monitoring of Partial Pressure of Carbon Dioxide in Mechanically Ventilated Preterm Infants. 2019. [DOI: ]

**TriHealth 2018**

TriHealth, Inc. Delayed Clamping and Milking the Umbilical Cord in Preterm Infants. 2018. [DOI: ]

**Universityof 2017**

University of, Sydney; National, Health; Medical Research Council, Australia; Baylor College of, Medicine. The Australian Placental Transfusion Study (APTS): Should Very Pre Term Babies Receive a Placental Blood Transfusion at Birth Via Deferring Cord Clamping Versus Standard Cord Clamping Procedures? 2017. [DOI: ]

**Universityof 2017a**

University of, Chicago. Delayed Cord Clamping in Very Low Birth Weight Infants. 2017. [DOI: ]

**Universityof 2019**

University of, Edinburgh; Lothian, N. H. S.. Measuring Oxygenation of Newborn Infants in Targeted Oxygen Ranges. 2019. [DOI: ]

**UniversityofCalifornia 2018**

University of California, Los Angeles. Cord Milking Impacts Neurodevelopmental Outcomes in Very Low Birth Weight Infants. 2018. [DOI: ]

**WeillMedicalCollegeofCornell 2019**

Weill Medical College of Cornell, University. Delayed Cord Clamping in Preterm Neonates. 2019. [DOI: ]
