# Supplementary material for: Interventions to minimize blood loss in very preterm infants—A systematic review and meta-analysis
Source: PLoS One. 2021 Feb 8;16(2):e0246353. doi: 10.1371/journal.pone.0246353 (PMC7870155; doi:10.1371/journal.pone.0246353)
Supplement: S4 File — (DOCX) [file pone.0246353.s005.docx]

### Ongoing studies

### In total 24 ongoing studies were identified

### Delayed cord clamping or cord milking compared to immediate clamping or no milking: 22 studies

### Blood sampling from the cord or from the placenta versus blood sampling from the infant: 0 studies

### Devices to reintroduce the blood after analysis: 0 studies

### Devices to monitor glucose levels, subcutaneous: 1 study

### Devices to monitor CO2 levels, transcutaneous or end-tidal versus blood sampling: 0 studies

1. Devices to monitor O2 levels, transcutaneous or intra-arterial versus blood sampling: 0 studies
2. Micro-methods for blood analysis: 1 study

**Allison 2019**

| **Study name** | REACT Trial |
| --- | --- |
| **Methods** | Parallel group randomized control trial |
| **Participants** | Inclusion criteria: Neonates weighing< 1200 grams  Exclusion criteria: Major congenital malformation, underlying metabolic disorder, neonates who, in the opinion of the treating clinician, have no realistic prospect of survival |
| **Interventions** | All babies will have CGM for data collection  Intervention group: The real time sensor data will be used for clinical management.  Control group: Will get standard care and the sensor data will be collected blind to the clinical team. |
| **Outcomes** | The primary endpoint for the trial is the difference between the arms in the percentage of time sensor glucose is in the target range of 2.6-10 mmol/l within the first six days of life.  Secondary outcomes include efficacy relating to glucose control, utility including staff acceptability, safety outcomes relating to incidence and prevalence of hypoglycemia and health economic analyses. |
| **Starting date** | July 2016 |
| **Contact information** | REACT@paed.cam.ac.uk or telephone 01223 746414 |

**Ctri 2013**

| **Study name** | Delayed versus early umbilical cord clamping in preterm infants less than 34 weeks of gestation |
| --- | --- |
| **Methods** | Parallel group randomized trial |
| **Participants** | Inclusion criteria: Infants born before 34 weeks of gestation  Exclusion criteria: Known congenital malformations, serious maternal illnesses (severe preeclampsia or eclampsia, third stage PPH, uncompensated heart disease), twins or triplets, babies requiring resuscitation |
| **Interventions** | Intervention group: Umbilical cord clamping delayed for 120 seconds after the birth of baby.  Control group: Umbilical cord clamping performed within 30 seconds after the birth of baby |
| **Outcomes** | Primary outcome: hyperbilirubinemia and polycythemia during initial 7 days of life in infants.  Secondary outcomes: Requirement for resuscitation, skin temperature at 5 minutes and 30 minutes of age, incidence of respiratory distress syndrome, culture positive or culture negative sepsis, hypoperfusion requiring fluid boluses and/or vasopressors, need for blood transfusion, intraventricular hemorrhage, NICU and hospital stay |
| **Starting date** | 15/04/2013 |
| **Contact information** | Anubhuti Rana  anubhuti_rana@yahoo.com |

**Ctri 2016**

| **Study name** | Delayed cord clamping in Rh isoimmunised infants |  |
| --- | --- | --- |
| **Methods** | Parallel group randomized trial |  |
| **Participants** | Inclusion criteria: Rh isoimmunised infants at time of birth from 28wks 0 days to 41 weeks 6 days willing for follow up till 12 weeks postnatal age.  Exclusion criteria: Babies with major congenital anomalies, Rh isoimmunised babies born to Retro positive mother, babies born with significant antepartum hemorrhage due to causes like abruptio placentae (significant antepartum bleed into placenta after separation of decidua), cord prolapsed ( protrusion of cord along with or ahead of delivery of baby) and cord anomalies like true knot all of which are associated with significant peripartum asphyxia and need immediate resuscitation. |  |
| **Interventions** | Intervention group: Delayed cord clamping for 60 seconds after delivery in Rh isoimmunised infants.  Control group: Immediate cord clamping in Rh isoimmunised infants. |  |
| **Outcomes** | Primary outcome: To measure Packed cell volume within 2(±1)hr. of birth, in Rh isoimmunised infants randomized to early cord clamping group or late cord clamping group, with gestation age more than 28weeks and 0 days to 41weeks and 6 days.  Secondary outcomes: Incidence of blood transfusion in initial 12weeks (±2) weeks of age in Rh isoimmunised infants, number of babies undergoing partial exchange transfusion (PET), number of babies undergoing double volume exchange transfusion (DVET), total duration of phototherapy (PT), echocardiographic evidence of fluid overload and hemodynamic compromise. |  |
| **Starting date** | 01/01/2017 |  |
| **Contact information** | Dr Ashok Deorari  ashokdeorari_56@hotmail.com |  |

**Ctri 2017a**

| **Study name** | The effects of umbilical cord milking in neonates requiring resuscitation at birth: a randomized controlled trial |
| --- | --- |
| **Methods** | Randomized, Parallel Group Trial |
| **Participants** | Inclusion criteria: Any neonate > 28 weeks gestation delivered in Obstetrics and Gynecology department, KGMU requiring resuscitation will be enrolled.  Exclusion criteria: Antenatally detected major congenital anomalies, cord prolapse, placenta previa or development of placental abruption, hydrops, umbilical cord abnormalities like true knot, refusal by the obstetrician |
| **Interventions** | Intervention group: Umbilical Cord Milking group (UCM): In the Umbilical cord milking group the cord will be milked thrice towards baby at a speed of 10 cm/s, and then clamped at 2-3 cm from umbilicus.  Control group: Immediate Cord Clamping group (ICC): The cord will be clamped and cut immediately (within 30 sec) |
| **Outcomes** | Primary outcome:   - Hemoglobin and Hct at birth and 6 weeks of age.   Secondary outcome:   - Apgar scores at birth - Chronic lung disease during hospitalization and follow up - Hematocrit at 6 hrs. And 24 hours of life - Hyperkalemia during hospitalization - Mean arterial pressures at 6 hrs. And 24 hrs. Of life - Mortality during hospitalization - Necrotizing enterocolitis during hospitalization - Need for inotropes in the first 48 to 72 hrs. - Need for transfusion during hospitalization and follow up - Neurodevelopmental outcome at 6 and 12 months of corrected age - Patent ductus arteriosus during hospitalization - Peak jaundice during hospitalization - Periventricular leukomalacia during hospitalization and follow up - Requirement of phototherapy and exchange transfusion during hospitalization - Respiratory distress syndrome during hospitalization - Resuscitation required at birth - Retinopathy of prematurity during hospitalization and follow up - Sepsis during hospitalization - Severe intraventricular hemorrhage during hospitalization - Time in days from birth to transfusion during hospitalization and follow up - Total volume of red cells transfused in first 28 days of life - Umbilical cord ph at birth |
| **Starting date** | 31-08-2017 |
| **Contact information** | Arpita Bhriguvanshi, arpime84@gmail.com |

**Ctri 2017b**

| **Study name** | Early cord clamping versus delayed cord clamping in very low birth weight neonates |
| --- | --- |
| **Methods** | Randomized, Parallel Group Trial |
| **Participants** | Inclusion criteria: All the neonates delivered by either mode (Normal Vaginal delivery/LSCS) in Kasturba hospital, Manipal (during study period) with a birth weight less than 1500gms will be included in the study.  Exclusion criteria: Congenital anomalies, MSAF, non vigorous neonates, suspected maternal hemorrhage (APH/PPH), monochorionic monoamniotic twins & twin-twin transfusion syndrome, OA incompatibility (Confirmed hemolysis), severe IUGR |
| **Interventions** | Intervention group: Umbilical cord clamping is delayed for 30 seconds after delivery to promote increased blood flow to neonate.  Control group: Cord clamping before 10 sec of delivery of baby. |
| **Outcomes** | Primary outcome: To study the effect of early vs delayed cord clamping on hemodynamic stability, hematological status, serum ferritin and the requirement for blood transfusion between birth and 6 months of age in very low birth weight neonates.  Secondary outcomes: To study incidence of major morbidities (respiratory distress syndrome, necrotizing enterocolitis, intraventricular hemorrhage, retinopathy of prematurity, late onset sepsis) between early versus delayed cord clamping in very low birth weight neonates. To study adverse outcomes like significant hyperbilirubinemia and polycythemia. |
| **Starting date** | 02/02/2017 |
| **Contact information** | Dr Leslie Edward Lewis  leslielewis1@gmail.com |

**Ctri 2018**

| **Study name** | Early versus Delayed Cord clamping in IUGR Preterms a Randomized controlled study |
| --- | --- |
| **Methods** | Randomized, Parallel Group, Active Controlled Trial |
| **Participants** | Inclusion criteria: SGA/IUGR preterm newborn babies born at <34 weeks either by vaginally or by caesarean section at our hospital and who gave written informed consent for the study. Gestational age will be determined by last menstrual period (LMP). If LMP will not be available then it will be determined by ultrasonography.  Exclusion criteria: Rh immunization, syndromic babies, APH and neonates requiring resuscitation. |
| **Interventions** | Intervention group: Delayed cord clamping  Control group: Early cord clamping |
| **Outcomes** | Primary outcome: To estimate the level of ferritin and PCV at 8 weeks for infants born at less than 34 completed weeks.  Secondary outcomes: Rate of anemia, polycythemia, number or volume of blood transfusions; treatment for hyperbilirubinemia with phototherapy; treatment for hyperbilirubinemia with blood exchange transfusion and number of exchange transfusions between groups. compare the need of Volume (colloid, sodium chloride 0.9 %, blood transfusion) administration for hypotension during the first 24 hours of life and inotropic support for hypotension during the first 24 hours of life between groups. |
| **Starting date** | 21/08/2018 |
| **Contact information** | Dr Sandeep Kadm  drsandeepkadam@gmail.com |

**Ctri 2019**

| **Study name** | Effects of delayed cord clamping in intrauterine growth restricted neonates: A randomized controlled trial |
| --- | --- |
| **Methods** | Randomized, Parallel Group Trial |
| **Participants** | Inclusion criteria: All antenatally diagnosed intra-uterine growth restricted neonates (based on ultrasonographically determined fetal weight below 10th percentile for gestational age) of gestational age 28 weeks or more born in AIIMS, Rishikesh during the study period will be eligible for inclusion.  Exclusion criteria: 1.Neonates born to mother with: a.Hemodynamic instability b.Placental previa/abruption c.Postpartum hemorrhage d.Multiple gestation e.Rh- negative blood group. 2.Neonate requiring resuscitation 3.Major congenital malformation 4.Fetal Hydrops 5.Failure to give consent |
| **Interventions** | Intervention group: Delayed cord clamping, after delivery, cord clamping will be done after 60 seconds  Control group: Early cord clamping, after delivery, cord clamping will be done within 30 seconds |
| **Outcomes** | Primary outcome: Superior vena cava blood flow and hematocrit value in intrauterine growth restricted neonates at 12-24 hours of life.  Secondary outcome: 1.Superior mesenteric blood flow at 12-24 hours of life 2.Morbidity during hospital stay i)Intraventricular hemorrhage ii)Symptomatic polycythemia iii)Respiratory distress requiring respiratory support iv)Unconjugated hyperbilirubinemia v)Feeding intolerance vi)Confirmed NEC (Modified Bell Stage II and beyond) 3.Duration of hospital stay 4.Outcome- discharge/death 5.Serum Ferritin at 3 month of age 6.BERA at 3 month of age 7.Denver II score at 3 months to 9 months follow up |
| **Starting date** | 11/05/2019 |
| **Contact information** | Kanhu Charan Digal  drkanhu.ped@gmail.com |

**Irct20100512003915N 2019**

| **Study name** | Comparison of the short term outcome of preterm infants with three different umbilical cord clamping methods |
| --- | --- |
| **Methods** | Randomized controlled trial |
| **Participants** | Inclusion criteria: Preterm infants with gestation age 28-30 weeks, birth weight less than 1500 grams.  Exclusion criteria: Infants with congenital anomalies, placenta previa, decolman, parental refuse or need to immediate maternal or neonatal resuscitation. |
| **Interventions** | Intervention group 1: Delayed cord clamp is done 30-60 seconds after birth.  Intervention group 2: Cord milking is done from 20 ±2 cm toward the neonate for 20 seconds three times.  Control group: Routine cord clamping is done 10 seconds after birth. |
| **Outcomes** | Intraventricular hemorrhage, bronchopulmonary dysplasia, anemia of prematurity |
| **Starting date** | 2019-03-11 |
| **Contact information** | Manizheh Mostafa Gharehbaghi  peirovifara@tbzmed.ac.ir |

**Irct2014031116936N 2014**

| **Study name** | Comparing the effect of delayed versus immediate cord clamping on the incidence of intraventricular hemorrhage (IVH) in preterm neonates with gestational age ≤34 weeks in Hafez & Zeynab hospitals from September 2012 to December 2013 |
| --- | --- |
| **Methods** | Randomized, double blinded, parallel group trial |
| **Participants** | Inclusion criteria: Premature neonates with gestational age 26-34 weeks born in Shiraz Hafez and Hazrat Zeynab hospitals from September 2012 to December 2013.  Exclusion criteria: Decline to participate, refuse of neonate logiest or obstetrician, severe congenital anomalies, need for immediate resuscitation after birth in neonate or mothers, placenta abruption or placenta previa, umbilical cord clamped in a time other than what is considered. |
| **Interventions** | Intervention group: 30-45 seconds delay in clamping of umbilical cord.  Control group: Umbilical cord clamping in 10 to15 seconds after birth. |
| **Outcomes** | Primary outcome:   - IVH by cranial ultrasonography on 3-4 day and 7-10 day after birth.   Secondary outcomes:   - Hemoglobin the first day after intervention - Hematocrit the first day after intervention - Platelet the first day after intervention and when needed after that - Bilirubin on 2nd, 3nd and fifth days after intervention if needed after that up to the discharge time - Apgar score 1st and 5th minute after intervention. |
| **Starting date** | 2012-08-22 |
| **Contact information** | Dr. Namavar Jahromi Bahia  namavarb@sums.ac.ir |

**Irct20180201038586N 2018**

| **Study name** | Investigation and comparison of neonatal complications of two methods of umbilical cord milking and early cord clamping in neonates |
| --- | --- |
| **Methods** | Parallel group randomized trial |
| **Participants** | Inclusion criteria: The infant's parents should give written informed consent for the infant to enter the study all infants who are not suspected of placental abruption For a gestational age of 28 to 34 weeks, in sonography, there should be no sign of placenta previa, anemia, hydrops and embryonic major abnormalities.  Exclusion criteria: Less than 25 cm length of umbilical cord Thick meconium The true knot of the umbilical cord Major anomalies of umbilical cord In a case that the infant is not hospitalized in NICU or during the study, the parents do not agree with the presence of their infant in the study. |
| **Interventions** | Intervention group: Umbilical cord milking  Control group: Early cord clamping |
| **Outcomes** | Primary outcomes: The amount of transfused blood in the first 120 hours, the amount of bilirubin measured daily.  Secondary outcomes: None reported |
| **Starting date** | 2017-09-23 |
| **Contact information** | Atena Sadat  sadata931@mums.ac.ir |

**Isrctn 2013**

| **Study name** | Randomized study of delayed cord clamping versus early cord clamping in preterm infants born between 24 and 34 weeks |
| --- | --- |
| **Methods** | Randomized controlled trial |
| **Participants** | Inclusion criteria: Women will be eligible for the study if they are likely to have a live birth before 34 weeks gestation, regardless of mode of birth or whether cephalic or breech presentation, singleton or dichorionic pregnancies.  Exclusion criteria: Major fetal abnormalities (defined as those that are lethal or require prenatal or postnatal surgery), fetal growth restriction, monochorionic twins, maternal pathology (pregestational diabetes, severe cardiopathy, infectious disease, hypertension and/or preeclampsia, obstetrics complications (abruptio, etc.). |
| **Interventions** | Intervention group: Delayed cord clamping: clamping of the cord 45-60 seconds after birth  Control group: Early cord clamping: clamping of the cord within ten seconds after birth |
| **Outcomes** | Primary outcome:   - Evaluation of neonatal hemoglobin, hematocrit and bilirubin levels within the first 7 days after birth   Secondary outcome:   - Neonatal hemoglobin, hematocrit and ferritin at six months of life will be evaluated by blood sampling - Neonatal complications (Intraventricular hemorrhage, necrotizing enterocolitis, retinopathy, sepsis, respiratory problems, days on ventilation or oxygen, need for phototherapy, transfusions) and days in the neonatal intensive care will be evaluated by medical history review - Cardiac output in the first week after birth will be measured by echocardiography - Blood loss in the mother (blood test 48 hours after birth) - Neurodevelopmental assessment of newborns at the age of two-three years in both groups of the study will be test by Bayley Scales of Infant Development |
| **Starting date** | 01/02/2011 |
| **Contact information** | Dr. Catalina De Paco Matallana  katy.depaco@gmail.com |

**Nct 2007**

| **Study name** | Delayed Umbilical Cord Clamping in Infants Less Than 32 Weeks: A Randomized Controlled Trial |
| --- | --- |
| **Methods** | Randomized control trial |
| **Participants** | Inclusion criteria: Women in labor or with a plan for delivery at a gestational age between 24 0/7 to 32 0/7 weeks gestation. Singleton pregnancy (Note: if pregnancy was a multiple gestation but demised occurred prior to 13 weeks, these patients can be included in study).  Exclusion criteria: Moderate to life threatening fetal anomalies, multiple live gestations at birth (e.g. twins, triplets, etc.), intrauterine fetal demise, previous participation, stem cell collection |
| **Interventions** | Intervention group: Delayed umbilical cord clamping 30-45 seconds.  Control group: Immediate umbilical cord clamping |
| **Outcomes** | Primary outcome: Composite outcome of Intraventricular Hemorrhage and/or Late Onset Sepsis  Secondary outcome: Lung function, cardiovascular function and anemia. |
| **Starting date** | November 2007 |
| **Contact information** | Kellie E. Murphy, MD MSc  kmurphy@mtsinai.on.ca |

**Nct 2014**

| **Study name** | Effects of Delayed Cord Clamp and/or Indomethacin on Preterm Infant Brain Injury |
| --- | --- |
| **Methods** | Parallel group randomized trial |
| **Participants** | Inclusion criteria: pregnant women admitted >24weeks and <30weeks gestational age, in-hospital birth (allowing for cord clamp randomization).  Exclusion criteria: preterm infant <24weeks or >30weeks at birth, maternal risks identified by obstetrician, fetal risks identified by obstetrician, any congenital abnormality of newborn infant, placental abruption/placental previa, delivery less than 2hrs from consenting to study participation |
| **Interventions** | Placebo Comparator: immediate cord clamp & placebo IV solution  Experimental: delay cord clamp & placebo IV solution  Active Comparator: immediate cord clamp & indomethacin IV  Experimental: indomethacin iv & delayed cord clamp |
| **Outcomes** | Primary outcome: fraction of survivors with no severe IVH (grades 3 or 4) or PVL [ Time Frame: within first 60 days of life]  Secondary outcome: occurrence of renal injury and/or dysfunction [ Time Frame: first 60 days of life],  hematological status, inflammatory stress, circulating progenitor cell subpopulations, neurocognitive assessments at post-NICU follow-up |
| **Starting date** | August 2014 |
| **Contact information** | Vicki Whitehead, RN, UK Section of Neonatology |

**Nct 2017**

| **Study name** | The Study on Umbilical Cord Milking to Prevent and Decrease the Severity of Anemia in preterms, a Multi-center Randomized Controlled Trial |
| --- | --- |
| **Methods** | Parallel group, randomized trial |
| **Participants** | Inclusion criteria: Women in labor or with a plan for delivery at a gestational age less than 34 weeks gestation, singleton pregnancy, informed consent was obtained from the parent  Exclusion criteria:  Multiple gestation,  Diagnosis of any of the following in the current pregnancy: hemorrhage requiring clinic/hospital admission, placental abnormalities, fetal anomalies, Down's syndrome of the fetus, anemia  Diagnosis of pre-eclampsia or eclampsia in current or previous pregnancies  Diagnosis at any time for the mother of any of the following: serious Diabetes, serious hypertension, chronic renal disease  Infant with major congenital malformation  Infant with blood disease  Unwilling to return for follow-up study visits at the hospital |
| **Interventions** | Intervention group: umbilical cord milking  Control group: umbilical cord clamping immediately |
| **Outcomes** | Primary outcome:   - Concentrations of Hemoglobin (Hb) [ Time Frame: 48 hours after birth] - Concentrations of Hematocrit(Hct) [ Time Frame: 48 hours after birth ] - Serum ferritin level [ Time Frame: 48 hours after birth   Secondary outcome:   - Hyperbilirubinemia requiring phototherapy [ Time Frame: during first 2 weeks of age ] - Hyperbilirubinemia requiring phototherapy (as per routine unit practice) - Incidence and numbers of blood transfusions [ Time Frame: until 3 months corrected gestational age ] - Length of admission [ Time Frame: Birth to discharge, expected average of 30 days ] - Complication [ Time Frame: Birth to discharge, expected average of 30 days ] - Composite of bronchopulmonary dysplasia, necrotizing enterocolitis, grade 3 or 4 intraventricular hemorrhage or periventricular leukomalacia, or death prior to discharge home |
| **Starting date** | June 30, 2017 |
| **Contact information** | Lijuan Xie, Principal Investigator, Xinhua Hospital, Shanghai Jiao Tong University School of Medicine |

**Nct 2018a**

| **Study name** | The Influence of Cut-umbilical Cord Milking (C-UCM) on the Cerebral Oxygenation and Perfusion of Preterm and Term Infants |
| --- | --- |
| **Methods** | Parallel group, randomized trial |
| **Participants** | Inclusion criteria:   - Neonates with a gestational age ≥28 weeks - Delivered by caesarean section - Decision to conduct full life support - Written informed consent prior to birth   Exclusion criteria:   - Neonates with a gestational age <28 weeks - No decision to conduct full life support - No written informed consent - Severe congenital malformations |
| **Interventions** | Intervention group: Milking the cut umbilical cord once towards the Infant at a speed of 10cm/second.  Control group: The umbilical cord is cut according to the standard procedure and no C-UCM is performed. |
| **Outcomes** | Primary outcome:  Cerebral blood volume [ Time Frame: within the first 15 minutes after birth. ] changes in CBV (ml/100g brain)  Secondary outcome:  Cerebral tissue oxygenation index [ Time Frame: within the first 15 minutes after birth ], changes in cTOI (%) Peripheral oxygen saturation [ Time Frame: within the first 15 minutes after birth ], changes in SpO2 (%) Heart rate [ Time Frame: within the first 15 minutes after birth ], changes in HR (beats per minute) Stroke volume [ Time Frame: within the first 15 minutes after birth ], changes in SV (ml) Cardiac output [ Time Frame: within the first 15 minutes after birth ], changes in CO (l/min) Mean arterial blood pressure [ Time Frame: within the first 15 minutes after birth ], changes in MABP (mmHg) |
| **Starting date** | November 21, 2018 |
| **Contact information** | Gerhard Pichler, MD, +43 316 385 50520,  gerhard.pichler@medunigraz.at |

**Nct 2018b**

| **Study name** | Impact of Umbilical Cord Milking in Preterm Neonates With Placental Insufficiency |
| --- | --- |
| **Methods** | Parallel group, randomized trial |
| **Participants** | Inclusion criteria: preterm neonates < 34 weeks gestational age  Exclusion criteria:  Vaginal bleeding due to placental abruption or tears, multiple pregnancies, suspected major fetal anomalies, suspected chromosomal aberration, maternal drug abuse, hydrops fetalis, preterm who needed major resuscitative measures |
| **Interventions** | Group A: umbilical cord milking will be done for preterm infants <34 gestational age without placental insufficiency Intervention: Procedure: Umbilical Cord Milking Group B: umbilical cord milking will be done for preterm infant <34 gestational age with placental insufficiency Intervention: Procedure: Umbilical Cord Milking Group C: Immediate cord clamping for preterm infants <34 gestational age with placental insufficiency |
| **Outcomes** | Primary outcome:   - Peripheral venous CD34 at admission   Secondary outcome:   - Admission hemoglobin - Hemoglobin at 2 months - Admission platelets - Admission WBCs - Phototherapy requirements - Polycythemia - Culture proven sepsis - Intraventricular hemorrhage of all grades - Bronchopulmonary dysplasia - Need for packed RBCs transfusion - Retinopathy of prematurity - Need for inotropes - Need for nasal CPAP - Need for mechanical ventilation - Duration of oxygen therapy |
| **Starting date** | December 1, 2017 |
| **Contact information** | Islam Nour, MD, +201003893026, inour2001@gmail.com |

**Nct 2018c**

| **Study name** | Effect of Delayed Cord Clamping in Preterm Neonates With Placental Insufficiency |
| --- | --- |
| **Methods** | Parallel group, randomized trial |
| **Participants** | Inclusion criteria: preterm neonates < 34 weeks gestational age  Exclusion criteria: vaginal bleeding due to placental abruption or tears, multiple pregnancies, suspected major fetal anomalies, suspected chromosomal aberration, maternal drug abuse, hydrops fetalis, preterm who needed major resuscitative measures |
| **Interventions** | Group A:  Placental insufficiency and ICC: Immediate cord clamping after delivery of the fetus in preterm infants with placental insufficiency  Group B:  Placental insufficiency and DCC: Cord clamping 60 seconds after delivery of fetus in preterm infants with placental insufficiency  Group C:  Normal placenta with DCC: Cord clamping 60 seconds after delivery of fetus in preterm infants without placental insufficiency |
| **Outcomes** | Primary outcome:   - Peripheral venous CD34 at admission   Secondary outcome:   - Admission hemoglobin - Hemoglobin at 2 months - Admission platelets - Admission WBCs - Phototherapy requirements - Polycythemia - Culture proven sepsis - Necrotizing enterocolitis - Intraventricular hemorrhage - Bronchopulmonary dysplasia - Need for nasal CPAP - Need for mechanical ventilation - Duration of oxygen therapy - Need for inotropes - Retinopathy of prematurity - Need for packed RBCs transfusion |
| **Starting date** | December 1, 2017 |
| **Contact information** | Islam Nour, MD, +201003893026, inour2001@gmail.com |

**NCT03200301 2017**

| **Study name** | Effect of Intact Umbilical Cord Milking Versus Immediate Cord Clamping on Neonatal Outcomes and First Year Neurodevelopmental Outcomes in Very Preterm Infants - A Randomized Controlled Trial |
| --- | --- |
| **Methods** | Parallel group, randomized trial |
| **Participants** | Inclusion criteria: All the preterm infants of less than 32 weeks of gestation born of consenting parents in the hospital  Exclusion criteria: Neonates depressed at birth, MCMA, MCDA twin pregnancy (DCDA twins are included), severe IUGR in antenatal scans (< 10th centile), known case of hydrops fetalis, known major congenital anomaly, placenta previa and abruptions, bleeding, accreta, nuchal cord, anhydramnios, PROM > 2 weeks before 24 weeks and Refusal to Consent by the Parents |
| **Interventions** | Intervention group: Intact umbilical cord milking  Control group: Early cord clamping |
| **Outcomes** | Primary outcome:   - Hemoglobin levels at birth - Incidence and severity of Intraventricular Hemorrhage in the first week of life   Secondary outcome:   - The resuscitation interventions required with and without umbilical cord milking. - Resuscitation outcomes with and without umbilical cord milking. - Incidence of hypotension - Symptomatic polycythemia - Sepsis (culture positive) - Peak bilirubin levels - Requirement of phototherapy or exchange transfusion - Incidence of Necrotizing Enterocolitis (NEC) - Requirement of Oxygen - Requirement of red blood cell transfusions - Number of days of Hospital Stay after Birth - Death prior to discharge - Serum iron stores - Developmental Assessment Scales for Indian Infants (DASII) |
| **Starting date** | April 1, 2018 |
| **Contact information** | Manoj Varanattu,MD, +919388407588,manojvaranattu@gmail.com |

**Nct 2020**

| **Study name** | A Randomized Controlled Intervention, Multi-centre Study Aiming to Preserve Blood Factors Using Micro-methods to Improve Development in Extremely Preterm Infants - "Less is More" |
| --- | --- |
| **Methods** | Parallel group, randomized control trial |
| **Participants** | Inclusion criteria: Gestational age < 27 weeks at birth  Exclusion criteria: Major malformation |
| **Interventions** | Intervention group: Micro methods for blood sample analysis  Control group: Standard clinical methods for blood sample analysis |
| **Outcomes** | Primary outcome:   - Broncho-pulmonary dysplasia   Secondary outcome:   - Cerebral intraventricular hemorrhage. Stage II, III and IV (Periventricular hemorrhagic infarction) - Necrotizing enterocolitis. Stage 2-3, Bells criteria (X-ray + clinical signs) - Blood transfusions administered during the first two postnatal weeks - Fetal Hemoglobin at 7 and 14 postnatal days |
| **Starting date** | March 15, 2020 |
| **Contact information** | \| David Ley, MD, PhD  david.ley@med.lu.se \|  \| \| --- \| --- \| |

**Tctr 2017**

| **Study name** | The Effect of One-Time Umbilical Cord Milking and Early Cord Clamping in Preterm Infants: A Randomized Controlled Trial (One-Time Umbilical Cord Milking) |
| --- | --- |
| **Methods** | Parallel group, randomized control trial |
| **Participants** | Inclusion criteria: Preterm<32weeks gestation who was born at Ramathibodi Hospital  Exclusion criteria: Placenta previa or accreta and cord problem, major congenital anomalies, hydrops fetalis, twin to twin transfusion syndrome, parents refuse to join the project |
| **Interventions** | Intervention group: One-time umbilical cord milking  Control group: Early cord clamping |
| **Outcomes** | Primary outcome:   - Hb and Hct level at admission   Secondary outcome:   - Hct - BP - Inotropic drugs and fluid - Resuscitation - Urine output, - Total amount of blood transfusion - Morbidity |
| **Starting date** | March 01, 2016 |
| **Contact information** | Sopida Tanthawat  sopida_tanth@hotmail.com |

**Tctr 2018**

| **Study name** | Delayed cord clamping reduced anemic outcome in preterm neonate |
| --- | --- |
| **Methods** | Parallel group, randomized control trial |
| **Participants** | Inclusion criteria: Viable singleton preterm pregnancy at 24-36+6 weeks of gestation and underwent vaginal delivery at the labor room and Cesarean delivery at the operating room  Exclusion criteria: Major severe congenital anomalies or chromosomal abnormalities, multiple gestations, maternal coagulopathy or maternal anemia (Hematocrit < 30%), placenta previa or abruptio placenta, fetal non reassuring or fetal distress, non-vigorous neonate were denied participation. |
| **Interventions** | Intervention group: Delayed cord clamping  Control group: Immediate cord clamping |
| **Outcomes** | Primary outcome:   - Hematocrit at 2 and 48 hrs.   Secondary outcome:   - Jaundice need phototherapy - Apgar score - Retained placenta - Postpartum hemorrhage |
| **Starting date** | 20/08/2018 |
| **Contact information** | Phiset Jomjak, Baddi_nip@hotmail.com |

**Tctr 2019a**

| **Study name** | A Randomized Controlled Trial of Umbilical Cord Milking versus Immediate Umbilical Cord Clamping in Preterm infants of Multiple Births Delivered by Cesarean Section |
| --- | --- |
| **Methods** | Randomized controlled trial |
| **Participants** | Inclusion criteria: Pregnant woman with twin gestations who deliver preterm infants at gestational age of 28-36+6 weeks gestation by cesarean section.  Exclusion criteria: Prenatally diagnosed major congenital anomaly in any infants, twin to twin transfusion syndrome (TTTS), twin anemic polycythemic sequence (TAPS), discordant twin (weight difference more than 20%), hydrops fetalis, antepartum or intrapartum hemorrhage such as placental abruption or uterine rupture. |
| **Interventions** | Intervention group: Umbilical cord milking (UCM)  Control group: Immediate cord clamping (ICC) |
| **Outcomes** | Primary outcome:   - Superior vena cava (SVC) flow within 6-24 hours after birth   Secondary outcome:   - Infants hematocrit level at 6-8 weeks after birth - Infants' hematocrit level at birth, within 6 hours - Infants' serum ferritin at 6-8 weeks after birth - Other maternal and infants' relevance clinical outcomes |
| **Starting date** | 31/05/2019 |
| **Contact information** | Muthita Meesang, muthita_nan@hotmail.com |

**Tctr 2019b**

| **Study name** | Comparison of Three Types of Placental transfusion in Preterm Infants: A Randomized Controlled Trial |
| --- | --- |
| **Methods** | Randomized controlled trial |
| **Participants** | Inclusion criteria: Preterm infants were born at 28 0/7 to 33 6/7 weeks of gestational age.  Exclusion criteria: Twin pregnancy, congenital lethal anomaly or chromosome abnormality e.g. trisomy 13, trisomy 18, hydrops fetalis, required NCPR, maternal history of antepartum hemorrhage, prenatal history of intrauterine growth retardation |
| **Interventions** | Intervention group 1: Delayed cord clamping with umbilical cord milking before cord clamping (DCM-B)  Intervention group 2: Delayed cord clamping with umbilical cord milking after cord clamping (DCM-A)  Control group: Delayed cord clamping (DCC) |
| **Outcomes** | Primary outcome:   - Received red blood cell transfusion at 28 days after end of the intervention   Secondary outcome:   - Superior vena cava blood (SVC) flow within 24 hours after the intervention |
| **Starting date** | 09/02/2016 |
| **Contact information** | Sariya Prachukthum, sariyarpd238@yahoo.com |

**University of 2020**

| **Study name** | Delivery Room Assistance With the Placental Circulation Intact: Effects on Early Postnatal Adaptation and Outcome of Preterm Babies. Study Protocol for a Randomized Control Trial (the PCI-trial) |
| --- | --- |
| **Methods** | Parallel group randomized trial |
| **Participants** | Inclusion criteria: Preterm newborns with gestational age between 23+0 and 29+6 weeks, informed consent available  Exclusion criteria: Major congenital malformation, hydrops fetalis, placental abruption, Rh isoimmunization, twin pregnancy, absence of informed consent |
| **Interventions** | Intervention group: Bedside assistance with placental circulation intact during first 3 minutes of life  Control group: Neonatal assistance in a standard setting after cord milking (milking four times 20 cm of cord) |
| **Outcomes** | Primary outcome:   - Reduction of incidence of severe intraventricular hemorrhage - Chronic lung disease or mortality   Secondary outcome:   - None reported |
| **Starting date** | April 9, 2016 |
| **Contact information** | Simone Pratesi, MD  simone.pratesi@unifi.it |

References:

**Allison 2019**

Allison, A.; Bond, S.; Beardsall, K.; Guy, C.; Pantaleo, B.; Thomson, L.. Real time continuous glucose monitoring in neonatal intensive care (REACT): Statistical challenges from the REACT trial. Trials 2019;20. [DOI: 10.1186/s13063-019-3688-6]

Forman, J.; Bond, S. J.; Guy, C.; Dunger, D.; Beardsall, K.. Methodological challenges in the react study, a randomized controlled trial of real time continuous glucose monitoring in neonatal intensive care. Trials 2017;18. [DOI: 10.1186/s13063-017-1902-y]

**Ctri 2013**

Ctri. Does delaying cutting of umbilical cord benefit the baby? http://www.who.int/trialsearch/Trial2.aspx?TrialID=CTRI/2013/04/003529 2013. [DOI: ]

**Ctri 2016**

Ctri. Delayed cord clamping in Rh-isoimmunised infants: an open labelled randomized control trial. http://www.who.int/trialsearch/Trial2.aspx?TrialID=CTRI/2016/12/007592 2016. [DOI: ]

**Ctri 2017a**

Ctri. The effects of umbilical cord squeezing in newborn babies requiring some form of intervention to help and support breathing at birth. http://www.who.int/trialsearch/Trial2.aspx?TrialID=CTRI/2017/08/009484 2017. [DOI: ]

**Ctri 2017b**

Ctri. COMPARISION OF ADVANTAGES AND DISADVANTAGES OF EARLY AND DELAYED UMBILICAL CORD CLAMPING IN LOW BIRTH WEIGHT NEWBORNS. http://www.who.int/trialsearch/Trial2.aspx?TrialID=CTRI/2017/01/007671 2017. [DOI: ]

**Ctri 2018**

Ctri. Role of delayed cord clamping in growth retarded preterms in increasing iron stores. http://www.who.int/trialsearch/Trial2.aspx?TrialID=CTRI/2018/08/015204 2018. [DOI: ]

**Ctri 2019**

Ctri. Effects of late cord clamping in small babies. http://www.who.int/trialsearch/Trial2.aspx?TrialID=CTRI/2019/05/018904 2019. [DOI: ]

**Irct20100512003915N 2019**

Irct20100512003915N. the short term outcome of three different umbilical cord clamping methods. http://www.who.int/trialsearch/Trial2.aspx?TrialID=IRCT20100512003915N22 2019. [DOI: ]

**Irct2014031116936N 2014**

Irct2014031116936N. The effect of delayed cord clamping on IVH in premature neonate. http://www.who.int/trialsearch/Trial2.aspx?TrialID=IRCT2014031116936N1 2014. [DOI: ]

**Irct20180201038586N 2018**

Irct20180201038586N. Investigation and comparison of neonatal complications of two methods of umbilical cord milking and early cord clamping in neonates. http://www.who.int/trialsearch/Trial2.aspx?TrialID=IRCT20180201038586N1 2018. [DOI: ]

**Isrctn 2013**

Isrctn. Delayed Cord Clamping versus early cord clamping in preterm infants born between 24 and 34 weeks. http://www.who.int/trialsearch/Trial2.aspx?TrialID=ISRCTN66018314 2013. [DOI: ]

**NCT03200301 2017**

NCT03200301; Jubilee Mission Medical, College; Research Institute, Yes. Effect of Intact Umbilical Cord Milking on Neonatal and First Year Neurodevelopmental Outcomes in Very Preterm Infants.. 2017. [DOI: ]

**Nct 2007**

Nct. Delayed Umbilical Cord Clamping in Infants Less Than 32 Weeks. https://clinicaltrials.gov/show/NCT00562536 2007. [DOI: ]

**Nct 2014**

Nct. Effects of Delayed Cord Clamp and/or Indomethacin on Preterm Infant Brain Injury. https://clinicaltrials.gov/show/NCT02221219 2014. [DOI: ]

**Nct 2017**

Nct. The Study on Umbilical Cord Milking to Prevent and Decrease the Severity of Anemia in Preterms. https://clinicaltrials.gov/show/NCT03023917 2017. [DOI: ]

**Nct 2018a**

Nct. C-UCM and Cerebral Oxygenation and Perfusion. https://clinicaltrials.gov/show/NCT03748914 2018. [DOI: ]

**Nct 2018b**

Nct. Impact of Umbilical Cord Milking in Preterm Neonates With Placental Insufficiency. https://clinicaltrials.gov/show/NCT03731611 2018. [DOI: ]

**Nct 2018c**

Nct. Effect of Delayed Cord Clamping in Preterm Neonates With Placental Insufficiency. https://clinicaltrials.gov/show/NCT03731546 2018. [DOI: ]

**Nct 2020**

Nct. Preservation of Blood in Extremely Preterm Infants (“Less Is More”) <https://clinicaltrials.gov/show/NCT04239690> 2020

**Tctr 2017**

Tctr. The Effect of One-Time Umbilical Cord Milking and Early Cord Clamping in Preterm Infants: A Randomized Controlled Trial. http://www.who.int/trialsearch/Trial2.aspx?TrialID=TCTR20170201003 2017. [DOI: ]

**Tctr 2018**

Tctr. Delayed cord clamping reduced anemic outcome in preterm neonate. http://www.who.int/trialsearch/Trial2.aspx?TrialID=TCTR20180817001 2018. [DOI: ]

**Tctr 2019a**

Tctr. A Randomized Controlled Trial of Umbilical Cord Milking versus Immediate Umbilical Cord Clamping in Preterm infants of Multiple Births Delivered by Cesarean Section. http://www.who.int/trialsearch/Trial2.aspx?TrialID=TCTR20190511002 2019. [DOI: ]

**Tctr 2019b**

Tctr. Comparison of Three Types of Placental transfusion in Preterm Infants: A Randomized Controlled Trial. http://www.who.int/trialsearch/Trial2.aspx?TrialID=TCTR20190131002 2019. [DOI: ]

**University of 2020**

University of, Florence. Delivery Room Assistance With the Placental Circulation Intact. 2020. [DOI: ]
