# Supplementary material for: Interventions to minimize blood loss in very preterm infants—A systematic review and meta-analysis
Source: PLoS One. 2021 Feb 8;16(2):e0246353. doi: 10.1371/journal.pone.0246353 (PMC7870155; doi:10.1371/journal.pone.0246353)
Supplement: S5 File — (DOCX) [file pone.0246353.s006.docx]

**Studies awaiting classification**

In total 13 studies awaiting classification were identified (all on cord clamping management).

**Aladangady 2006**

| **Methods** | Randomized control trial |
| --- | --- |
| **Participants** | Inclusion criteria: Singleton deliveries at gestational age 24+0 - 32+6 |
| **Interventions** | Delayed cord clamping vs Early cord clamping |
| **Outcomes** | Blood volume at approximately 4 hours of life |
|  |  |

**Das 2018**

| **Methods** | Randomized, parallel group trial |
| --- | --- |
| **Participants** | Inclusion criteria: Imminently delivering mothers from 30 weeks to 33+6 week, parents have given informed consent.  Exclusion criteria: Major congenital malformation incompatible for life diagnosed or suspected antenatally, multiple pregnancy, conditions where placental transfusion by delaying cord clamping could further compromise the just delivered neonate due to the additional blood volume being transfused such as antenatally diagnosed hydrops fetalis (irrespective of etiology or type of hydrops) |
| **Interventions** | Intervention group: Delayed cord clamping for 60 seconds from the birth of the baby  Control group: Early cord clamping group (10 seconds from birth of the baby) |
| **Outcomes** | Primary outcome: Composite outcome measure of all cause mortality and/or abnormal neurological examination at 40 weeks postnatal age  Secondary outcome: Incidence of following at 40 weeks postnatal age :  1. All cause mortality  2. Intraventricular hemorrhage  3. bronchopulmonary dysplasia  4. Necrotizing enterocolitis  5. Retinopathy of prematurity  6. Hematocrit, number of blood transfusions  7. Significant hyperbilirubinemia  8. Serum ferritin levels at discharge and 3 months postnatal age |
|  |  |

**Hofmeyr 1993**

| **Methods** | Randomized controlled trial |
| --- | --- |
| **Participants** | Women expected to give birth to babies weighing less than 2000 g |
| **Interventions** | Intervention group: Delaying cord clamping for 1 to 2 min.  Control group: clamping the umbilical cord immediately after delivery |
| **Outcomes** | Ultrasound diagnosis of PVH/IVH about 24 h after birth |
|  |  |

**Khalid 2013**

| **Methods** | Randomized controlled trial |
| --- | --- |
| **Participants** | Not reported |
| **Interventions** | Intervention group: Delayed cord clamping  Control group: Immediate cord clamping |
| **Outcomes** | Hemoglobin and serum bilirubin at birth  Serum bilirubin at 6 hours of life. |
|  |  |

**Kinmond 1993**

| **Methods** | A prospective randomized study |
| --- | --- |
| **Participants** | 36 vaginally delivered infants over 27 and under 33 weeks' gestation |
| **Interventions** | Holding the infant 20 cm below the introitus for 30 seconds before clamping the umbilical cord ("regulated" group, 17 patients), or conventional management ("random" group, 19 patients). |
| **Outcomes** | Initial packed cell volume, peak serum bilirubin concentrations, red cell transfusion requirements, and respiratory impairment (assessed by ventilatory requirements, arterial-alveolar oxygen tension ratio over the first day in ventilated infants, and duration of dependence on supplemental oxygen). |
|  |  |

**Li 2020**

| **Methods** | Randomized controlled trial |
| --- | --- |
| **Participants** | Neonates vaginally delivered between 28 and 37 weeks’ gestation and complicated by PPROM before birth |
| **Interventions** | Intervention group: Intact umbilical cord milking before clamping  Control group: Immediate cord clamping |
| **Outcomes** | Hematological parameters (hemoglobin, hematocrit,platelet count, white blood count, neutrophil ratio, and C-reactive protein), neonatal outcomes (probable or certain neonatal infection, respiratory distress syndrome, necrotizing enterocolitis, and intraventricular hemorrhage) |
|  |  |

**McDonnell 1997**

| **Methods** | Randomized controlled trial |
| --- | --- |
| **Participants** | Infants born at 26-33 weeks gestation |
| **Interventions** | Intervention group: Umbilical cord clamped 30 s after birth  Control group: Umbilical cord clamped immediately after birth |
| **Outcomes** | Venous haematocrit at 1 and at 4 h of age. |
|  |  |

**NCT02187874 2014**

| **Methods** | Randomized controlled study |
| --- | --- |
| **Participants** | Inclusion Criteria:   - Deliveries ( either vaginal or by C-section) between 26 and 32.6 weeks of gestation. - Patients must be over 18 years old. - Patient understands and signs informed consent.   Exclusion Criteria:   - Urgent C-section - Gestational age under 22 or over 33 weeks - Major fetal anomalies (requiring surgery or with a high risk of neonatal death or incapacity) - Major uterine malformations - Placenta previa. - Multiple gestations - Fetal hydrops - Severe Iso- Immunization - HIV-positive mother - Severe Intrauterine growth restriction ( Reverse atrial Flow in DV) - Intrauterus Ventricular haemorrhage |
| **Interventions** | Intervention group: Delayed umbilical cord occlusion  Control group: Early umbilical cord occlusion |
| **Outcomes** | Primary outcome:  Number of red blood cell transfusions to the newborn  Intraventricular Haemorrhage incidence  Maternal postpartum haemorrhage incidence  Volume of neonatal red blood cell transfusions  Secondary outcome:  Neonatal mortality  APGAR score  Umbilical cord blood pH  Neonatal intubation  Incidence of intensive reanimation of the newborn Use of vasoactive drugs Incidence of adverse events during hospital stay of the newborn |
|  |  |

**Rana 2018**

| **Methods** | Randomized controlled trial |
| --- | --- |
| **Participants** | Infants born at less than 34 weeks' gestation |
| **Interventions** | Intervention group: Delayed cord clamping (120sec)  Control group: Immediate cord clamping (<30sec) |
| **Outcomes** | Hematocrit measurement at 48 hours and 7 days. Serum bilirubin levels were estimated once the infant had clinically significant jaundice or at 72 hours. |
|  |  |

**Ruangkit 2019**

| **Methods** | Randomized controlled trial |
| --- | --- |
| **Participants** | Multiple gestations who deliver preterm infants at gestational age (GA) of 28-36 weeks |
| **Interventions** | Intervention group: Delayed cord clamping (30-60sec)  Control group: Immediate cord clamping (<10sec) |
| **Outcomes** | Primary outcome:  Infants' hematocrit level at birth  Secondary outcome:  Infants' hematocrit level at 8 weeks after birth Other Maternal and infants' relevance clinical outcomes Superior Vena Cava (SVC) Flow |
|  |  |

**Song 2017**

| **Methods** | Randomized controlled study |
| --- | --- |
| **Participants** | Pregnant women who were expected to deliver at between 24 0/7 and 32 6/7 weeks of gestation |
| **Interventions** | Intervention group: Umbilical cord milking  Control group: Immediate cord clamping |
| **Outcomes** | Apgar scores at both 1 and 5 minutes, initial blood gas analysis results, body temperature at admission, need for early intubation, and maximum bilirubin level, neonatal hemoglobin levels at birth and at 24 hours of age, need for blood transfusion, use of inotropic drugs |
|  |  |

**Strauss 2007**

| **Methods** | Randomized controlled trial |
| --- | --- |
| **Participants** | Preterm neonates (≤36 weeks' gestation) |
| **Interventions** | Intervention group: Delayed cord clamping at 60 sec.  Control group: Immediate cord clamping |
| **Outcomes** | Primary outcome: Circulating RBC volume/mass  Secondary outcome: Multiple clinical and laboratory comparisons over the first 28 days including Score for Neonatal Acute Physiology (SNAP) |
|  |  |

**Zhang 2018**

| **Methods** | Randomized controlled trial |
| --- | --- |
| **Participants** | Neonates born<37 weeks |
| **Interventions** | Intervention group: Umbilical cord milking  Control group: Immediate cord clamping without milking |
| **Outcomes** | Clinical measures of the outcomes were measured by transcutaneous oxygen saturation (TcSO 2 ), hemoglobin at different periods, left heart function within 72 h, cranial ultrasound, transcutaneous bilirubin, the total time of phototherapy. |
|  |  |

References:

**Aladangady 2006**

Aladangady, Narendra; McHugh, Siobhan; Aitchison, Thomas C.; Wardrop, Charles A. J.; Holland, Barbara M. T. I. Infants; blood volume in a controlled trial of placental transfusion at preterm, delivery. Pediatrics 2006;117(1):93-8. [DOI: ]

**Das 2018**

CTRI/2014/02/004414; Pgimer No. Delayed cord clamping in preterm neonates 30 to 33 weeks: A randomized controlled trial. 2014. [DOI: ]

Ctri. Delayed cord clamping in preterm neonates 30 to 33 weeks: a randomized controlled trial. http://www.who.int/trialsearch/Trial2.aspx?TrialID=CTRI/2014/02/004414 2014. [DOI: ]

Das, B.; Sundaram, V.; Kumar, P.; Mordi, W. T.; Dhaliwal, L. K.; Das, R.. Effect of Placental Transfusion on Iron Stores in Moderately Preterm Neonates of 30-33 weeks Gestation. Indian J Pediatr 2018;85(3):172-178. [DOI: 10.1007/s12098-017-2490-2]

Das, B.; Sundaram, V.; Tarnow-Mordi, W.; Ghadge, A.; Dhaliwal, L. K.; Kumar, P.. Placental transfusion in preterm neonates of 30-33 weeks' gestation: a randomized controlled trial. J Perinatol 2018;38(5):496-504. [DOI: 10.1038/s41372-018-0064-4]

**Hofmeyr 1993**

Hofmeyr, G. J.; Gobetz, L.; Bex, P. J.; Van der Griendt, M.; Nikodem, C.; Skapinker, R.; Delahunt, T.. Periventricular/intraventricular hemorrhage following early and delayed umbilical cord clamping. A randomized controlled trial. Online J Curr Clin Trials 1993;Doc No 110:[2002 words; 26 paragraphs]. [DOI: ]

**Khalid 2013**

Khalid, N.; Shami, N.; Ijaz, M.. Role of delay in umbilical cord clamping in reducing frequency of neonatal anaemia. Pakistan Journal of Medical and Health Sciences 2013;7(3):824-825. [DOI: ]

**Kinmond 1993**

Kinmond, S.; Aitchison, T. C.; Holland, B. M.; Jones, J. G.; Turner, T. L.; Wardrop, C. A.. Umbilical cord clamping and preterm infants: a randomised trial. BMJ (Clinical research ed.) 1993;306(6871):172-5. [DOI: 10.1136/bmj.306.6871.172]

**Li 2020**

Li, J.; Yu, B.; Wang, W.; Luo, D.; Dai, Q. L.; Gan, X. Q.. Does intact umbilical cord milking increase infection rates in preterm infants with premature prolonged rupture of membranes? The journal of maternal-fetal & neonatal medicine : the official journal of the European Association of Perinatal Medicine, the Federation of Asia and Oceania Perinatal Societies, the International Society of Perinatal Obstetricians 2020;33(2):184-190. [DOI: 10.1080/14767058.2018.1487947]

**McDonnell 1997**

McDonnell, M.; Henderson-Smart, D. J.. Delayed umbilical cord clamping in preterm infants: a feasibility study. J Paediatr Child Health 1997;33(4):308-10. [DOI: ]

**NCT02187874 2014**

NCT02187874; Hospital Universitari Vall d'Hebron Research Institute, Yes. Timing of Umbilical Cord Occlusion in Premature Babies( &lt;33 w). Delayed vs Early.. 2014. [DOI: ]

**Rana 2018**

Rana, A.; Agarwal, K.; Ramji, S.; Gandhi, G.; Sahu, L.. Safety of delayed umbilical cord clamping in preterm neonates of less than 34 weeks of gestation: a randomized controlled trial. Obstetrics & gynecology science 2018;61(6):655-661. [DOI: 10.5468/ogs.2018.61.6.655]

Rana, Anubhuti; Agarwal, Krishna CY -, <Blank>. Safety of Delayed Umbilical Cord Clamping in Preterm Neonates Less than 34 weeks Gestation. Indian Journal of Pediatrics 2017;84:414-414. [DOI: 10.1007/s12098-016-2289-6]

**Ruangkit 2019**

Ruangkit, C.; Bumrungphuet, S.; Panburana, P.; Khositseth, A.; Nuntnarumit, P.. A Randomized Controlled Trial of Immediate versus Delayed Umbilical Cord Clamping in Multiple-Birth Infants Born Preterm. Neonatology 2019;115(2):156-163. [DOI: 10.1159/000494132]

TCTR20190511002; Faculty of Medicine Ramathibodi Hospital, Yes. A Randomized Controlled Trial of Umbilical Cord Milking versus Immediate Umbilical Cord Clamping in Preterm infants of Multiple Births Delivered by Cesarean Section. 2019. [DOI: ]

Tctr. A Randomized Controlled Trial of Immediate versus Delayed Umbilical Cord Clamping in Preterm Infants of Multiple Births. http://www.who.int/trialsearch/Trial2.aspx?TrialID=TCTR20170125001 2016. [DOI: ]

**Song 2017**

Song, S. Y.; Kim, Y.; Kang, B. H.; Yoo, H. J.; Lee, M.. Safety of umbilical cord milking in very preterm neonates: a randomized controlled study. Obstetrics & gynecology science 2017;60(6):527-534. [DOI: 10.5468/ogs.2017.60.6.527]

**Strauss 2007**

Strauss, R. G.; Mock, D. M.. A randomized clinical trial comparing immediate vs delayed clamping of the umbilical cord in preterm infants. Transfusion 2007;47 Suppl:21A. [DOI: ]

Strauss, Ronald G.; Mock, Donald M.; Johnson, Karen J.; Cress, Gretchen A.; Burmeister, Leon F.; Zimmerman, M. Bridget; Bell, Edward F.; Rijhsinghani, Asha. A randomized clinical trial comparing immediate versus delayed clamping of the umbilical cord in preterm infants: short-term clinical and laboratory endpoints. Transfusion 2008;48(4):658-65. [DOI: ]

**Zhang 2018**

Zhang, L.; Huang, Q. W.; Gong, X. H.; Li, P.. Early effects of umbilical cord milking on hemoglobin, bilirubin and cardiac function in premature infants. Journal of Shanghai Jiaotong University (Medical Science) 2018;38(10):1197-1202. [DOI: 10.3969/j.issn.1674-8115.2018.10.011]
