# Supplementary material for: Interventions to minimize blood loss in very preterm infants—A systematic review and meta-analysis
Source: PLoS One. 2021 Feb 8;16(2):e0246353. doi: 10.1371/journal.pone.0246353 (PMC7870155; doi:10.1371/journal.pone.0246353)
Supplement: Attachment — Submitted filename: Response to Reviewers- PONE-D-20-31636R1.pdf [file pone.0246353.s007.pdf]

15.12.2020

Dear Editor,

We thank the reviewers for their thoughtful comments on the manuscript and have adapted it to address their concerns.

We believe that the publication is now suitable for publication in PLOS ONE. We thank you again for taking our manuscript into consideration.

Best regards,
Matteo Bruschettini

We responded to each point raised by the academic editor and reviewers as follows:

Journal Requirements:
When submitting your revision, we need you to address these additional requirements.

1. Please ensure that your manuscript meets PLOS ONE's style requirements, including those for file naming. The PLOS ONE style templates can be found at https://journals.plos.org/plosone/s/file?id=wjVg/PLOSOne_formatting_sample_main_body.pdf and https://journals.plos.org/plosone/s/file?id=ba62/PLOSOne_formatting_sample_title_authors_affiliations.pdf We have adhered to the style requirements.

2. Please include your tables as part of your main manuscript and remove the individual files. Please note that supplementary tables should be uploaded as separate "supporting information" files. We made sure to include all tables in the manuscript that aren't supplementary.

3. We note that you have included the phrase "data not shown" in your manuscript. Unfortunately, this does not meet our data sharing requirements. PLOS does not permit references to inaccessible data. We require that authors provide all relevant data within the paper, Supporting Information files, or in an acceptable, public repository. Please add a citation to support this phrase or upload the data that corresponds with these findings to a stable repository (such as Figshare or Dryad) and provide and URLs, DOIs, or accession numbers that may be used to access these data. Or, if the data are not a core part of the research being presented in your study, we ask that you remove the phrase that refers to these data. The phrase "data not shown" was a direct quotation from the Widness et al. 2005 paper and was quoted to justify our grading of "unclear" for selective reporting.

4. Please include captions for your Supporting Information files at the end of your manuscript, and update any in-text citations to match accordingly. Please see our Supporting Information guidelines for more information: http://journals.plos.org/plosone/s/supporting-information. We have also adhered to the Supporting Information guidelines and included captions for our Supporting Information files.

Reviewer's Responses to Questions

**Comments to the Author**

1. Is the manuscript technically sound, and do the data support the conclusions?

The manuscript must describe a technically sound piece of scientific research with data that supports the conclusions. Experiments must have been conducted rigorously, with appropriate controls, replication, and sample sizes. The conclusions must be drawn appropriately based on the data presented.

Reviewer #1: Yes

---

2. Has the statistical analysis been performed appropriately and rigorously?
Reviewer #1: Yes

---

3. Have the authors made all data underlying the findings in their manuscript fully available?

The PLOS Data policy requires authors to make all data underlying the findings described in their manuscript fully available without restriction, with rare exception (please refer to the Data Availability Statement in the manuscript PDF file). The data should be provided as part of the manuscript or its supporting information, or deposited to a public repository. For example, in addition to summary statistics, the data points behind means, medians and variance measures should be available. If there are restrictions on publicly sharing data—e.g. participant privacy or use of data from a third party—those must be specified.
Reviewer #1: Yes

---

4. Is the manuscript presented in an intelligible fashion and written in standard English?

PLOS ONE does not copyedit accepted manuscripts, so the language in submitted articles must be clear, correct, and unambiguous. Any typographical or grammatical errors should be corrected at revision, so please note any specific errors here.
Reviewer #1: Yes

---

5. Review Comments to the Author

Please use the space provided to explain your answers to the questions above. You may also include additional comments for the author, including concerns about dual publication, research ethics, or publication ethics. (Please upload your review as an attachment if it exceeds 20,000 characters)
Reviewer #1: The authors present a well written systematic review and meta-analysis manuscript focusing on the potential influence of blood loss on common prematurity co-morbidities and interventions to prevent blood loss in the infants from birth. This is an important review and one of interest in many neonatologists. However, I have some minor issues and comments:

Minor issues:

1. Section "Results", page 17, sentence 414-415 : “Three studies were relevant for the comparison blood sampling from the umbilical cord or from the placenta vs. blood sampling from the infant”: The

studies using umbilical cord or from the placenta vs. blood sampling from the infant are two and not three. There is an inconsistency here, authors must verify it throughout the article. We have fixed the inconsistencies regarding the use of "two" and "three" studies and adjusted it throughout the paper where needed.

2. Section "Results", page 17, sentence 417-418 :, "two studies were relevant for the comparison devices to monitor glucose levels, subcutaneous vs. conventional blood sampling": The studies using u devices to monitor glucose levels, subcutaneous vs. conventional blood sampling are three and not two. There is an inconsistency here, authors must verify it throughout the article. We have fixed the inconsistencies regarding the use of "two" and "three" studies and adjusted it throughout the paper where needed.

Minor comments:

1. Section "Methods": Did the authors think that their search strategy was good enough to cover all the relevant studies? Why pre-specified the specific interventions? They could have missed relevant trials for the systematic review. Add maybe a suggested optimisation for this search strategy in the discussion section. The search strategy was built on search filters developed by Cochrane, which are very sensitive to make sure all possibly relevant studies are retrieved. The search strategy was tested on a set of key reference articles to see if they were captured. The initial search strategy did not capture all of them, so the search strategy was divided into separate searches for each intervention, which captured all the key reference articles. Using key reference articles and dividing the search strategies based on the specific interventions is a method often used for systematic review that include multiple interventions to make sure no relevant articles are missed. For systematic reviews patients, interventions of interest and study design (randomized studies) are the components that are specified in the search strategy. Outcomes are generally not included in the search to make sure it is sensitive enough to retrieve all possibly relevant studies. To further optimize study retrieval, a hand search could have been performed. However, such a complementary strategy is time consuming and the search strategy for this systematic review was considered to be sensitive enough to capture all possibly relevant studies.

2. Section "Methods": Why did the authors decide to apply no language restrictions to the search strategy? How did they handle the translations without any error or bias? It is highly desirable for systematic reviews to avoid language restrictions to make sure all potentially relevant studies are retrieved. If necessary, translation can be arranged, which it was in our case. The translations from Mandarin and Hebrew were made through connections established via Cochrane Task Exchange (more info: https://taskexchange.cochrane.org/), as commonly done in Cochrane reviews. Of note, both translators had experience in performing this task.

3. Section "Methods": Why did the authors decide to use the I2 statistic heterogeneity measure and not others, like Q statistic, H2 statistic, R2 statistic, etc.? We do agree that statistical heterogeneity measure might be explored with different tools and recognize that there is much uncertainty in measures, such as I2 and Tau2, especially when there are few studies. We used I2, however we are open to adding other measures, such as Q statistic, H2 statistic or R2 statistic, if considered preferable by the Reviewer or the Editor.

4. Section "Discussion", page 50, sentence 1216: Concerning the figures 7 and 8, it's not clear in this section to what correspond each figure. Authors must clarify the methods used for these funnel plots. These methods are not presented in the "method" section, including them will improve the

consistency of the manuscript. We have clarified what each figure corresponds to, the methods used for these funnel plots, and have labelled the difference between them.

5. Section "figures", Funnel plots, fig 7 and 8: Authors should label the difference between the two funnel plots in the figures' section as they are almost identical. We have clarified what each figure corresponds to, the methods used for these funnel plots, and have labelled the difference between them.
